# Supplementary material for: Antibiotic use attributable to specific aetiologies of diarrhoea in children under 2 years of age in low-resource settings: a secondary analysis of the MAL-ED birth cohort
Source: BMJ Open. 2022 Apr 1;12(4):e058740. doi: 10.1136/bmjopen-2021-058740 (PMC8977746; doi:10.1136/bmjopen-2021-058740)
Supplement: Supplementary data [file bmjopen-2021-058740supp001.pdf]

Supplementary Online Content

Antibiotic use attributable to etiology-specific diarrhea in children under two years of age in low-resource settings

Stephanie A Brennhofer, James A Platts-Mills, Joseph A Lewnard, Jie Liu, Eric R Houpt, Elizabeth T Rogawski McQuade

Brennhofer SA, *et al. BMJ Open* 2022; 12:e058740. doi: 10.1136/bmjopen-2021-058740

Table S1. Attributable incidence of pathogen-specific antibiotic courses for diarrhea by antibiotic drug class among 1715 children in the MAL-ED cohort.

	Any antibiotic	Cephalosporins	Fluoroquinolones	Macrolides	Metronidazole	Penicillins	Sulfonamides
Shigella	14.77 (13.25, 16.84)	2.14 (1.66, 2.76)	3.09 (2.64, 3.66)	4.59 (3.92, 5.47)	4.96 (4.09, 5.97)	1.69 (1.29, 2.23)	1.66 (1.27, 2.15)
Rotavirus	10.90 (9.75, 12.42)	1.51 (1.11, 2.01)	1.79 (1.37, 2.28)	3.33 (2.78, 4.02)	3.53 (2.86, 4.37)	1.78 (1.32, 2.40)	1.35 (0.92, 1.85)
Sapovirus	10.24 (8.37, 12.55)	1.47 (0.93, 2.10)	1.48 (1.09, 1.90)	2.75 (2.16, 3.41)	3.95 (2.87, 5.28)	1.57 (1.16, 2.09)	1.56 (1.17, 2.07)
Adenovirus 40/41	9.63 (8.27, 11.69)	1.01 (0.73, 1.43)	1.75 (1.42, 2.22)	4.43 (3.74, 5.35)	1.59 (1.03, 2.47)	1.88 (1.49, 2.48)	0.68 (0.46, 1.00)
ST-ETEC	8.56 (7.04, 10.71)	0.85 (0.56, 1.36)	1.63 (1.24, 2.12)	2.74 (2.11, 3.50)	2.51 (1.83, 3.53)	1.40 (0.99, 2.01)	0.99 (0.64, 1.44)
Astrovirus	6.72 (5.22, 8.77)	1.44 (0.98, 2.11)	0.75 (0.48, 1.09)	1.68 (1.17, 2.36)	2.36 (1.60, 3.38)	1.20 (0.86, 1.71)	0.99 (0.70, 1.41)
Norovirus	6.14 (4.85, 8.03)	0.51 (0.26, 0.97)	0.59 (0.37, 0.86)	1.48 (1.08, 1.99)	1.97 (1.26, 3.12)	1.45 (1.07, 2.04)	1.16 (0.85, 1.63)
C. jejuni/C. coli	4.61 (3.14, 7.19)	0.63 (0.32, 1.10)	0.44 (0.21, 0.83)	1.47 (0.95, 2.33)	1.25 (0.48, 2.37)	0.87 (0.52, 1.33)	0.88 (0.58, 1.31)
Cryptosporidium	2.83 (1.97, 4.15)	0.39 (0.19, 0.73)	0.38 (0.18, 0.62)	0.55 (0.27, 0.86)	0.87 (0.52, 1.58)	0.67 (0.42, 1.13)	0.45 (0.24, 0.71)
tEPEC	2.54 (1.27, 4.69)	0.51 (0.13, 1.11)	0.18 (0.08, 0.39)	0.38 (0.14, 0.79)	1.32 (0.43, 2.80)	0.56 (0.26, 1.08)	0.43 (0.20, 0.83)

Data are attributable incidence rates per 100 child years with 95% confidence intervals (CIs). These data also reported in Figure 1A. Abbreviations: *C. jejuni/C. coli* = *Campylobacter jejuni/Campylobacter coli.* ST-ETEC= heat-stable enterotoxigenic *Escherichia coli*. tEPEC = typical enteropathogenic *Escherichia coli*.

Table S2. Pathogen-specific attributable fractions of antibiotic courses for diarrhea by antibiotic drug class among 1715 children in the MAL-ED cohort.

	Any Antibiotic	Cephalosporins	Fluoroquinolones	Macrolides	Metronidazole	Penicillins	Sulfonamides
Shigella	11.7 (10.5, 13.3)	8.9 (6.9, 11.5)	20.9 (17.9, 24.7)	16.2 (13.8, 19.3)	10.3 (8.5, 12.4)	7.0 (5.4, 9.3)	9.8 (7.5, 12.7)
Rotavirus	8.6 (7.7, 9.8)	6.3 (4.6, 8.4)	12.1 (9.3, 15.4)	11.8 (9.8, 14.2)	7.3 (5.9, 9.0)	7.4 (5.5, 10.0)	8.0 (5.4, 10.9)
Sapovirus	8.1 (6.6, 9.9)	6.2 (3.9, 8.8)	10.0 (7.4, 12.9)	9.7 (7.6, 12.0)	8.2 (5.9, 10.9)	6.5 (4.8, 8.7)	9.2 (6.9, 12.2)
Adenovirus 40/41	7.6 (6.5, 9.3)	4.2 (3.0, 6.0)	11.8 (9.6, 15.0)	15.6 (13.2, 18.9)	3.3 (2.1, 5.1)	7.8 (6.2, 10.3)	4.0 (2.7, 5.9)
ST-ETEC	6.8 (5.6, 8.5)	3.6 (2.3, 5.7)	11.0 (8.4, 14.4)	9.7 (7.4, 12.4)	5.2 (3.8, 7.3)	5.8 (4.1, 8.4)	5.8 (3.8, 8.5)
Astrovirus	5.3 (4.1, 6.9)	6.0 (4.1, 8.8)	5.1 (3.3, 7.4)	5.9 (4.1, 8.3)	4.9 (3.3, 7.0)	5.0 (3.6, 7.1)	5.9 (4.2, 8.3)
Norovirus	4.9 (3.8, 6.4)	2.1 (1.1, 4.1)	4.0 (2.5, 5.8)	5.2 (3.8, 7.0)	4.1 (2.6, 6.5)	6.1 (4.4, 8.5)	6.8 (5.0, 9.6)
C. jejuni/C. coli	3.6 (2.5, 5.7)	2.6 (1.3, 4.6)	3.0 (1.4, 5.6)	5.2 (3.4, 8.2)	2.6 (1.0, 4.9)	3.6 (2.2, 5.5)	5.2 (3.4, 7.7)
Cryptosporidium	2.2 (1.6, 3.3)	1.6 (0.8, 3.1)	2.6 (1.2, 4.2)	1.9 (1.0, 3.0)	1.8 (1.1, 3.3)	2.8 (1.7, 4.7)	2.6 (1.4, 4.2)
tEPEC	2.0 (1.0, 3.7)	2.1 (0.5, 4.6)	1.2 (0.5, 2.6)	1.3 (0.5, 2.8)	2.7 (0.9, 5.8)	2.3 (1.1, 4.5)	2.6 (1.2, 4.9)

Data are attributable fraction percentages with 95% confidence intervals (CIs). These data also reported in Figure 2A. Abbreviations: *C. jejuni/C. coli* = *Campylobacter jejuni/Campylobacter coli*. ST-ETEC= heat-stable enterotoxigenic *Escherichia coli*. tEPEC = typical enteropathogenic *Escherichia coli*.

Brennhofer SA, *et al. BMJ Open* 2022; 12:e058740. doi: 10.1136/bmjopen-2021-058740

Table S3. Pathogen-specific attributable fractions of antibiotic courses for diarrhea by site among 1715 children in the MAL-ED cohort.

	Dhaka, Bangladesh	Fortaleza, Brazil	Vellore, India	Bhaktapur, Nepal	Loreto, Peru	Naushero Feroze, Pakistan	Venda, South Africa	Tanzania
Shigella	21.4 (18.6, 25.6)	10.6 (0.0, 29.6)	17.0 (11.8, 23.3)	15.0 (11.1, 19.9)	11.6 (8.7, 14.9)	6.4 (4.8, 8.5)	6.9 (0.0, 15.0)	7.9 (1.0, 16.0)
Rotavirus	16.0 (13.7, 19.1)	0.0 (0.0, 0.0)	12.2 (7.6, 17.4)	7.9 (5.4, 11.0)	5.5 (3.9, 7.5)	5.5 (4.4, 7.1)	3.3 (0.0, 11.6)	12.4 (6.1, 19.9)
Sapovirus	8.5 (5.5, 11.4)	15.1 (0.0, 37.7)	9.9 (5.8, 14.7)	9.6 (6.2, 13.5)	11.6 (9.0, 14.7)	5.8 (3.3, 8.7)	3.6 (0.2, 7.8)	10.9 (4.4, 18.2)
Adenovirus 40/41	23.9 (20.0, 29.1)	6.8 (0.0, 24.3)	7.9 (3.8, 13.0)	2.1 (0.1, 4.6)	6.5 (4.6, 9.0)	1.3 (0.1, 3.3)	1.6 (0.0, 4.6)	7.1 (1.7, 14.5)
ST-ETEC	14.7 (11.2, 18.9)	3.7 (0.0, 15.8)	7.3 (3.7, 11.7)	5.9 (3.5, 9.2)	3.2 (1.5, 5.6)	3.1 (1.7, 5.5)	5.4 (0.0, 15.6)	15.6 (6.6, 25.2)
Astrovirus	5.2 (2.4, 8.2)	0.0 (0.0, 0.0)	6.7 (3.4, 11.3)	3.1 (1.5, 5.0)	8.8 (6.5, 12.2)	4.8 (2.8, 7.2)	6.2 (0.6, 16.1)	2.0 (0.0, 5.8)
Norovirus	5.0 (2.9, 7.1)	6.5 (0.0, 21.8)	2.9 (0.4, 5.9)	5.3 (3.2, 8.4)	8.9 (6.5, 11.8)	2.3 (0.9, 5.1)	4.5 (0.0, 13.2)	12.3 (6.3, 19.9)
C. jejuni/C. coli	2.7 (0.3, 6.8)	0.0 (0.0, 0.0)	4.6 (1.6, 8.2)	3.1 (0.6, 6.9)	9.6 (5.9, 13.4)	1.9 (0.1, 4.5)	2.2 (0.0, 7.3)	4.1 (0.0, 13.3)
Cryptosporidium	1.9 (0.6, 3.3)	3.7 (0.0, 13.5)	4.7 (1.9, 9.8)	2.5 (0.6, 4.7)	3.7 (1.9, 5.6)	1.5 (0.6, 2.9)	0.1 (0.0, 1.2)	2.9 (0.0, 8.7)
tEPEC	1.1 (0.1, 3.0)	0.0 (0.0, 1.5)	1.8 (0.2, 4.8)	1.2 (0.2, 3.3)	1.6 (0.2, 3.5)	2.5 (0.4, 5.8)	0.0 (0.0, 2.1)	4.4 (0.0, 12.9)

Data are attributable fraction percentages with 95% confidence intervals (CIs). Abbreviations: *C. jejuni/C. coli* = *Campylobacter jejuni/Campylobacter coli*. ST-ETEC= heat-stable enterotoxigenic *Escherichia coli*. tEPEC = typical enteropathogenic *Escherichia coli*.

Brennhofer SA, *et al. BMJ Open* 2022; 12:e058740. doi: 10.1136/bmjopen-2021-058740

Table S4. Pathogen-specific attributable fractions of all antibiotic courses by site among 1715 children in the MAL-ED cohort.

	Dhaka, Bangladesh	Fortaleza, Brazil	Vellore, India	Bhaktapur, Nepal	Loreto, Peru	Naushero Feroze, Pakistan	Venda, South Africa	Haydom, Tanzania
Shigella	5.2 (4.5, 6.2)	0.9 (0.0, 2.4)	2.4 (1.6, 3.2)	4.5 (3.3, 6.0)	3.9 (2.9, 5.0)	2.4 (1.8, 3.2)	0.8 (0.0, 1.8)	1.4 (0.2, 2.9)
Rotavirus	3.9 (3.3, 4.6)	0.0 (0.0, 0.0)	1.7 (1.1, 2.4)	2.4 (1.6, 3.3)	1.9 (1.3, 2.5)	2.1 (1.6, 2.6)	0.4 (0.0, 1.4)	2.3 (1.1, 3.7)
Sapovirus	2.1 (1.3, 2.8)	1.2 (0.0, 3.0)	1.4 (0.8, 2.0)	2.9 (1.9, 4.1)	3.9 (3.0, 5.0)	2.2 (1.2, 3.2)	0.4 (0.0, 1.0)	2.0 (0.8, 3.3)
Adenovirus 40/41	5.8 (4.9, 7.1)	0.5 (0.0, 2.0)	1.1 (0.5, 1.8)	0.6 (0.0, 1.4)	2.2 (1.5, 3.1)	0.5 (0.0, 1.2)	0.2 (0.0, 0.6)	1.3 (0.3, 2.7)
ST-ETEC	3.6 (2.7, 4.6)	0.3 (0.0, 1.3)	1.0 (0.5, 1.6)	1.8 (1.0, 2.8)	1.1 (0.5, 1.9)	1.2 (0.6, 2.0)	0.7 (0.0, 1.9)	2.9 (1.2, 4.6)
Astrovirus	1.3 (0.6, 2.0)	0.0 (0.0, 0.0)	0.9 (0.5, 1.6)	0.9 (0.5, 1.5)	3.0 (2.2, 4.1)	1.8 (1.1, 2.7)	0.8 (0.1, 2.0)	0.4 (0.0, 1.1)
Norovirus	1.2 (0.7, 1.7)	0.5 (0.0, 1.8)	0.4 (0.1, 0.8)	1.6 (1.0, 2.5)	3.0 (2.2, 4.0)	0.9 (0.3, 1.9)	0.5 (0.0, 1.6)	2.3 (1.2, 3.7)
C. jejuni/C. coli	0.6 (0.1, 1.7)	0.0 (0.0, 0.0)	0.6 (0.2, 1.1)	0.9 (0.2, 2.1)	3.2 (2.0, 4.5)	0.7 (0.0, 1.7)	0.3 (0.0, 0.9)	0.8 (0.0, 2.4)
Cryptosporidium	0.5 (0.1, 0.8)	0.3 (0.0, 1.1)	0.6 (0.3, 1.4)	0.8 (0.2, 1.4)	1.2 (0.6, 1.9)	0.5 (0.2, 1.1)	0.0 (0.0, 0.2)	0.5 (0.0, 1.6)
tEPEC	0.3 (0.0, 0.7)	0.0 (0.0, 0.1)	0.3 (0.0, 0.7)	0.4 (0.1, 1.0)	0.5 (0.1, 1.2)	0.9 (0.1, 2.2)	0.0 (0.0, 0.3)	0.8 (0.0, 2.4)

Data are attributable fraction percentages with 95% confidence intervals (CIs). Abbreviations: *C. jejuni/C. coli* = *Campylobacter jejuni/Campylobacter coli*. ST-ETEC= heat-stable enterotoxigenic *Escherichia coli*. tEPEC = typical enteropathogenic *Escherichia coli*.

Brennhofer SA, *et al. BMJ Open* 2022; 12:e058740. doi: 10.1136/bmjopen-2021-058740

Table S5. Attributable incidence of pathogen-specific antibiotic courses for diarrhea by site among 1715 children in the MAL-ED cohort.

	Dhaka, Bangladesh	Fortaleza, Brazil	Vellore, India	Bhaktapur, Nepal	Loreto, Peru	Naushero Feroze, Pakistan	Venda, South Africa	Haydom, Tanzania
Shigella	45.79 (39.70, 54.61)	0.58 (0.00, 1.62)	9.08 (6.27, 12.4)	10.57 (7.79, 14.01)	20.54 (15.49, 26.48)	23.93 (17.88, 31.62)	0.90 (0.00, 1.96)	5.12 (0.65, 10.42)
Rotavirus	34.20 (29.21, 40.74)	0.00 (0.00, 0.00)	6.48 (4.02, 9.29)	5.53 (3.79, 7.73)	9.83 (6.85, 13.26)	20.68 (16.33, 26.47)	0.43 (0.00, 1.51)	8.08 (3.94, 12.95)
Sapovirus	18.10 (11.79, 24.44)	0.82 (0.00, 2.06)	5.29 (3.09, 7.82)	6.71 (4.38, 9.49)	20.50 (16.03, 26.07)	21.64 (12.24, 32.38)	0.47 (0.03, 1.02)	7.10 (2.88, 11.86)
Adenovirus 40/41	50.99 (42.72, 62.14)	0.37 (0.00, 1.33)	4.23 (2.04, 6.91)	1.47 (0.10, 3.24)	11.52 (8.13, 16.04)	4.87 (0.32, 12.21)	0.20 (0.00, 0.60)	4.64 (1.14, 9.44)
ST-ETEC	31.42 (23.92, 40.30)	0.20 (0.00, 0.86)	3.90 (1.98, 6.21)	4.18 (2.45, 6.46)	5.67 (2.65, 9.92)	11.52 (6.48, 20.50)	0.70 (0.00, 2.04)	10.14 (4.27, 16.42)
Astrovirus	11.18 (5.02, 17.56)	0.00 (0.00, 0.00)	3.59 (1.81, 6.02)	2.20 (1.08, 3.55)	15.67 (11.49, 21.70)	17.74 (10.62, 27.00)	0.82 (0.07, 2.11)	1.29 (0.00, 3.77)
Norovirus	10.64 (6.13, 15.20)	0.36 (0.00, 1.19)	1.56 (0.22, 3.12)	3.74 (2.23, 5.89)	15.80 (11.53, 20.94)	8.76 (3.24, 19.05)	0.59 (0.00, 1.73)	8.01 (4.08, 12.98)
C. jejuni/C. coli	5.70 (0.68, 14.56)	0.00 (0.00, 0.00)	2.46 (0.83, 4.38)	2.20 (0.45, 4.82)	17.06 (10.47, 23.71)	6.96 (0.41, 16.90)	0.29 (0.00, 0.96)	2.67 (0.00, 8.62)
Cryptosporidium	4.08 (1.19, 7.00)	0.20 (0.00, 0.74)	2.48 (1.02, 5.24)	1.77 (0.41, 3.29)	6.47 (3.39, 9.97)	5.50 (2.43, 10.81)	0.01 (0.00, 0.16)	1.87 (0.00, 5.64)
tEPEC	2.43 (0.29, 6.41)	0.00 (0.00, 0.08)	0.99 (0.08, 2.56)	0.88 (0.14, 2.30)	2.82 (0.37, 6.23)	9.26 (1.49, 21.69)	0.00 (0.00, 0.28)	2.86 (0.00, 8.39)

Data are attributable incidence rates per 100 child years with 95% confidence intervals (CIs). These data also reported in Figure 1B. Abbreviations: *C. jejuni/C. coli* = *Campylobacter jejuni/Campylobacter coli*. ST-ETEC= heat-stable enterotoxigenic *Escherichia coli*. tEPEC = typical enteropathogenic *Escherichia coli*.

Brennhofer SA, *et al. BMJ Open* 2022; 12:e058740. doi: 10.1136/bmjopen-2021-058740

Figure S1. Attributable incidence of pathogen-specific antibiotic courses for diarrhea in the first and second year of life among 1715 children in the MAL-ED cohort. Error bars show 95% CI. *C. jejuni/C. coli* = *Campylobacter jejuni/Campylobacter coli*. ST-ETEC= heat-stable enterotoxigenic *Escherichia coli*. tEPEC = typical enteropathogenic *Escherichia coli*.

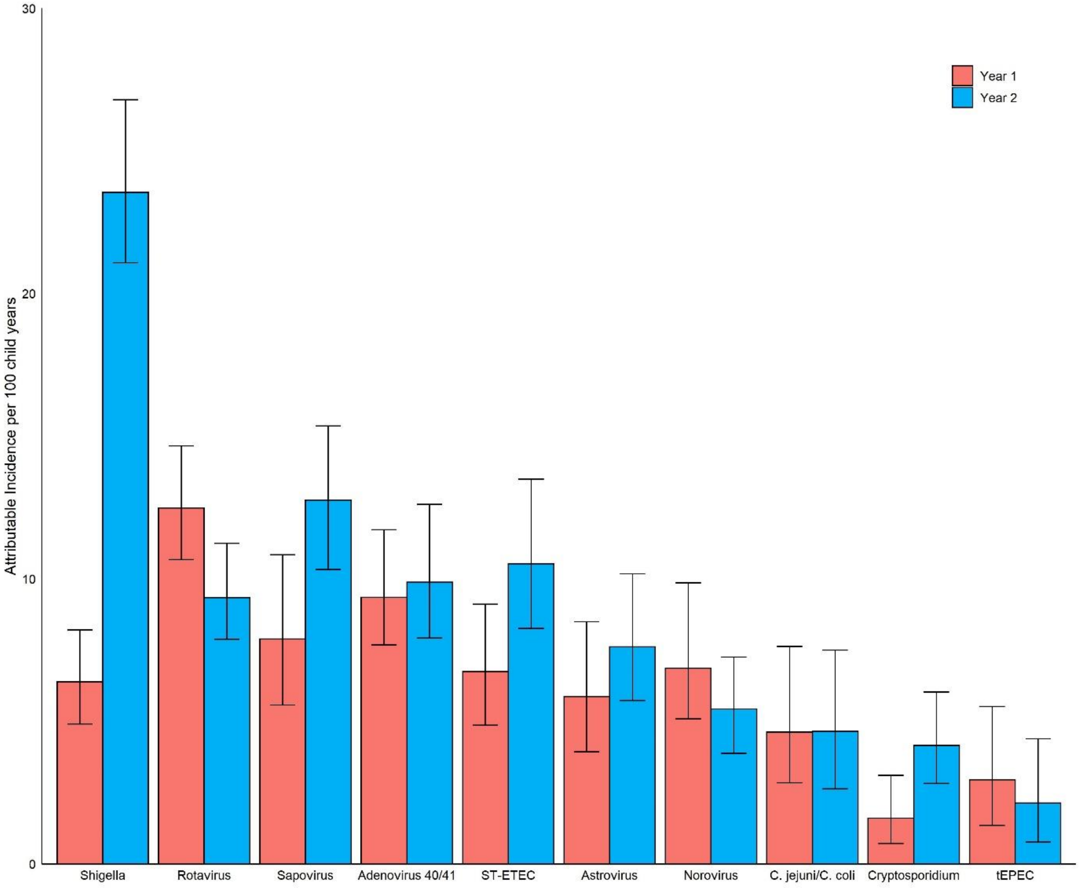

Table S6. Attributable incidence of pathogen-specific antibiotic courses for diarrhea in the first and second year of life among 1715 children in the MAL-ED cohort.

	Year 1 (0-11 months)	Year 2 (12-23 months)
Shigella	6.39 (4.91, 8.21)	23.55 (21.08, 26.79)
Rotavirus	12.48 (10.67, 14.65)	9.33 (7.87, 11.24)
Sapovirus	7.89 (5.58, 10.84)	12.75 (10.32, 15.36)
Adenovirus 40/41	9.35 (7.67, 11.71)	9.87 (7.92, 12.61)
ST-ETEC	6.74 (4.87, 9.10)	10.52 (8.26, 13.49)
Astrovirus	5.87 (3.94, 8.49)	7.61 (5.72, 10.17)
Norovirus	6.86 (5.09, 9.86)	5.43 (3.88, 7.25)
C. jejuni/C. coli	4.62 (2.85, 7.62)	4.65 (2.63, 7.49)
Cryptosporidium	1.60 (0.72, 3.10)	4.15 (2.82, 6.03)
tEPEC	2.95 (1.35, 5.52)	2.13 (0.77, 4.38)

Data are attributable incidence rates per 100 child years with 95% confidence intervals (CIs). These data also reported in Figure S1. Abbreviations: *C. jejuni/C. coli* = *Campylobacter jejuni/Campylobacter coli.* ST-ETEC= heat-stable enterotoxigenic *Escherichia coli*. tEPEC = typical enteropathogenic *Escherichia coli*.

Table S7. Pathogen-specific attributable fractions of all antibiotic courses by antibiotic drug class among 1715 children in the MAL-ED cohort.

	Any Antibiotic	Cephalosporins	Fluoroquinolones	Macrolides	Metronidazole	Penicillins	Sulfonamides
Shigella	3.2 (2.9, 3.7)	2.0 (1.6, 2.6)	12.2 (10.5, 14.5)	5.5 (4.7, 6.6)	6.7 (5.5, 8.0)	0.9 (0.7, 1.2)	3.6 (2.8, 4.7)
Rotavirus	2.4 (2.1, 2.7)	1.4 (1.1, 1.9)	7.1 (5.4, 9.0)	4.0 (3.3, 4.8)	4.7 (3.8, 5.9)	1.0 (0.7, 1.3)	3.0 (2.0, 4.1)
Sapovirus	2.2 (1.8, 2.7)	1.4 (0.9, 2.0)	5.9 (4.3, 7.5)	3.3 (2.6, 4.1)	5.3 (3.9, 7.1)	0.9 (0.6, 1.1)	3.4 (2.6, 4.5)
Adenovirus 40/41	2.1 (1.8, 2.6)	1.0 (0.7, 1.4)	6.9 (5.6, 8.8)	5.3 (4.5, 6.4)	2.1 (1.4, 3.3)	1.0 (0.8, 1.4)	1.5 (1.0, 2.2)
ST-ETEC	1.9 (1.5, 2.3)	0.8 (0.5, 1.3)	6.5 (4.9, 8.4)	3.3 (2.5, 4.2)	3.4 (2.5, 4.7)	0.8 (0.5, 1.1)	2.2 (1.4, 3.2)
Astrovirus	1.5 (1.1, 1.9)	1.4 (0.9, 2.0)	3.0 (1.9, 4.3)	2.0 (1.4, 2.8)	3.2 (2.1, 4.5)	0.7 (0.5, 0.9)	2.2 (1.5, 3.1)
Norovirus	1.3 (1.1, 1.8)	0.5 (0.2, 0.9)	2.3 (1.4, 3.4)	1.8 (1.3, 2.4)	2.6 (1.7, 4.2)	0.8 (0.6, 1.1)	2.5 (1.9, 3.6)
C. jejuni/C. coli	1.0 (0.7, 1.6)	0.6 (0.3, 1.0)	1.7 (0.8, 3.3)	1.8 (1.1, 2.8)	1.7 (0.6, 3.2)	0.5 (0.3, 0.7)	1.9 (1.3, 2.9)
Cryptosporidium	0.6 (0.4, 0.9)	0.4 (0.2, 0.7)	1.5 (0.7, 2.4)	0.7 (0.3, 1.0)	1.2 (0.7, 2.1)	0.4 (0.2, 0.6)	1.0 (0.5, 1.6)
tEPEC	0.6 (0.3, 1.0)	0.5 (0.1, 1.1)	0.7 (0.3, 1.5)	0.5 (0.2, 1.0)	1.8 (0.6, 3.8)	0.3 (0.1, 0.6)	0.9 (0.4, 1.8)

Data are attributable fraction percentages with 95% confidence intervals (CIs). These data also reported in Figure 2B. Abbreviations: *C. jejuni/C. coli* = *Campylobacter jejuni/Campylobacter coli*. ST-ETEC= heat-stable enterotoxigenic *Escherichia coli*. tEPEC = typical enteropathogenic *Escherichia coli*.

Brennhofer SA, *et al. BMJ Open* 2022; 12:e058740. doi: 10.1136/bmjopen-2021-058740

Table S8. Pathogen-specific attributable fractions of antibiotic courses for dysentery and watery diarrhea among 1,715 children in the MAL-ED cohort

	**Attributable fraction of antibiotic courses for dysentery**	**Attributable fraction of antibiotic courses for watery diarrhea**	**Proportion of all attributable antibiotic courses that were for dysentery**
Shigella	27.5% (23.4, 33.1)	10.3% (9.2, 11.8)	18.7% (16.2, 21.4)
Rotavirus	3.8% (2.3, 5.8)	9.1% (8.1, 10.4)	3.5% (2.1, 5.1)
Sapovirus	5.9% (3.8, 8.6)	8.3% (6.8, 10.2)	5.8% (3.9, 7.9)
Adenovirus 40/41	7.4% (5.6, 10.2)	7.6% (6.5, 9.3)	7.8% (5.9, 9.9)
ST-ETEC	5.7% (3.2, 8.4)	6.9% (5.7, 8.7)	6.7% (4.1, 9.1)
Astrovirus	2.6% (1.5, 4.3)	5.6% (4.3, 7.3)	3.9% (2.5, 5.8)
Norovirus	4.0% (2.3, 6.3)	4.9% (3.8, 6.5)	6.6% (4.0, 9.6)
C. jejuni/C. coli	8.5% (5.8, 12.2)	3.2% (2.1, 5.4)	18.6% (12.7, 25.5)
Cryptosporidium	1.3% (0.5, 3.0)	2.3% (1.6, 3.4)	4.7% (1.7, 9.0)
tEPEC	1.5% (0.5, 3.5)	2.1% (1.0, 3.8)	6.0% (2.2, 11.8)

Data are attributable fraction percentages with 95% confidence intervals (CIs). [a] Positive value indicates the pathogen was responsible for a larger proportion of antibiotic-treated dysentery diarrheal episodes compared to antibiotic-treated watery diarrhea episodes. Abbreviations: *C. jejuni/C. coli* = *Campylobacter jejuni/Campylobacter coli*. ST-ETEC= heat-stable enterotoxigenic *Escherichia coli*. tEPEC = typical enteropathogenic *Escherichia coli*.

Brennhofer SA, *et al. BMJ Open* 2022; 12:e058740. doi: 10.1136/bmjopen-2021-058740

Table S9 Risk ratios for antibiotic treatment comparing episode-specific attributable fractions for each pathogen by site among 1715 children in the MAL-ED cohort.

	Dhaka, Bangladesh	Vellore, India	Bhaktapur, Nepal	Loreto, Peru	Naushero Feroze, Pakistan	Haydom, Tanzania
Shigella	1.40 (1.22, 1.61)	1.55 (1.03, 2.33)	2.30 (1.70, 3.11)	1.35 (1.08, 1.68)	1.38 (1.17, 1.62)	0.77 (0.34, 1.72)
Rotavirus	1.10 (0.95, 1.27)	1.67 (1.13, 2.46)	0.70 (0.46, 1.05)	1.37 (1.06, 1.79)	1.48 (1.31, 1.68)	1.04 (0.64, 1.69)
Sapovirus	1.04 (0.83, 1.29)	0.93 (0.53, 1.62)	0.76 (0.49, 1.18)	0.86 (0.68, 1.09)	1.02 (0.84, 1.25)	1.23 (0.77, 1.98)
Adenovirus 40/41	1.01 (0.87, 1.18)	1.85 (0.84, 4.07)	1.07 (0.35, 3.21)	0.73 (0.49, 1.10)	..	1.65 (0.84, 3.26)
ST-ETEC	0.87 (0.74, 1.03)	0.84 (0.48, 1.46)	0.74 (0.48, 1.13)	0.91 (0.61, 1.36)	1.32 (1.01, 1.71)	0.87 (0.56, 1.37)
Astrovirus	1.00 (0.74, 1.34)	1.08 (0.57, 2.02)	0.54 (0.26, 1.11)	0.94 (0.70, 1.26)	1.08 (0.88, 1.34)	0.49 (0.10, 2.37)
Norovirus	1.40 (1.08, 1.80)	0.66 (0.20, 2.14)	0.58 (0.33, 1.04)	1.12 (0.83, 1.50)	1.36 (1.01, 1.83)	1.13 (0.67, 1.88)
C. jejuni/C. coli	0.66 (0.43, 1.02)	0.75 (0.33, 1.69)	..	1.43 (1.15, 1.78)	0.87 (0.58, 1.32)	..
Cryptosporidium	1.03 (0.71, 1.48)	2.11 (1.18, 3.79)	1.31 (0.64, 2.66)	0.72 (0.46, 1.15)	1.19 (0.83, 1.71)	3.18 (1.36, 7.43)
tEPEC	0.44 (0.22, 0.89)	..	0.41 (0.12, 1.38)	0.99 (0.54, 1.81)	0.97 (0.74, 1.28)	1.87 (0.98, 3.57)

Data are risk ratios (RR) with 95% confidence intervals (CIs). The model is adjusted for: all pathogens, age, sex, and the water, assets, maternal education, income (WAMI) index. Site data from Brazil and South Africa were removed as there were not enough diarrheal episodes to model the data. Select pathogen data by site is missing in cases where there were no treated diarrheal episodes. Abbreviations: *C. jejuni/C. coli* = *Campylobacter jejuni/Campylobacter coli*. ST-ETEC= heat-stable enterotoxigenic *Escherichia coli*. tEPEC = typical enteropathogenic *Escherichia coli*.

Brennhofer SA, *et al. BMJ Open* 2022; 12:e058740. doi: 10.1136/bmjopen-2021-058740

Table S10. Mediation analysis assessing whether diarrhea severity and dysentery mediated the relationship between Shigella diarrhea and antibiotic treatment among 1715 children in the MAL-ED cohort.

	Shigella (Mediated by diarrhea severity)		*Shigella* (Mediated by dysentery)	
	Any antibiotic	Fluoroquinolones or macrolides	Any antibiotic	Fluoroquinolones or macrolides
Total Effect Rate Ratio	1.30 (1.21, 1.40)	1.39 (1.21, 1.58)	1.30 (1.21, 1.40)	1.39 (1.21, 1.58)
Pure Natural Direct Effect Rate Ratio	1.29 (1.18, 1.40)	1.33 (1.14, 1.55)	1.25 (1.14, 1.37)	1.23 (1.05, 1.44)
Total Natural Indirect Effect Rate Ratio	1.01 (0.96, 1.06)	1.04 (0.95, 1.14)	1.04 (0.99, 1.10)	1.13 (1.02, 1.25)
Proportion Mediated	0.05 (0.00, 0.27)	0.14 (0.00, 0.47)	0.18 (0.00, 0.43)	0.40 (0.08, 0.82)

Data are risk ratios (RR) with 95% confidence intervals (CIs).

Brennhofer SA, *et al. BMJ Open* 2022; 12:e058740. doi: 10.1136/bmjopen-2021-058740
