# Supplementary material for: Antibiotic use attributable to specific aetiologies of diarrhoea in children under 2 years of age in low-resource settings: a secondary analysis of the MAL-ED birth cohort
Source: BMJ Open. 2022 Apr 1;12(4):e058740. doi: 10.1136/bmjopen-2021-058740 (PMC8977746; doi:10.1136/bmjopen-2021-058740)
Supplement: Author's manuscript [file bmjopen-2021-058740.draft_revisions.pdf]

BMJ Open is committed to open peer review. As part of this commitment we make the peer review history of every article we publish publicly available.

When an article is published we post the peer reviewers' comments and the authors' responses online. We also post the versions of the paper that were used during peer review. These are the versions that the peer review comments apply to.

The versions of the paper that follow are the versions that were submitted during the peer review process. They are not the versions of record or the final published versions. They should not be cited or distributed as the published version of this manuscript.

BMJ Open is an open access journal and the full, final, typeset and author-corrected version of record of the manuscript is available on our site with no access controls, subscription charges or pay-per-view fees (http://bmjopen.bmj.com).

If you have any questions on BMJ Open's open peer review process please email info.bmjopen@bmj.com

BMJ Open

Antibiotic use attributable to specific etiologies of diarrhea in children under two years of age in low-resource settings

Journal:	*BMJ Open*
Manuscript ID	bmjopen-2021-058740
Article Type:	Original research
Date Submitted by the Author:	27-Oct-2021

Complete List of Authors:	Brennhofer, Stephanie; University of Virginia School of Medicine, Division of Infectious Diseases and International Health<br>Platts-Mills, James; University of Virginia School of Medicine, Division of Infectious Diseases and International Health<br>Lewnard, Joseph; University of California Berkeley, Division of Epidemiology<br>Liu, Jie; University of Virginia School of Medicine, Division of Infectious Diseases and International Health<br>Houpt, Eric; University of Virginia School of Medicine, Division of Infectious Diseases and International Health<br>Rogawski McQuade, Elizabeth; University of Virginia School of Medicine, Division of Infectious Diseases and International Health; Emory University, Department of Epidemiology

Keywords:	Gastrointestinal infections < GASTROENTEROLOGY, Community child health < PAEDIATRICS, INFECTIOUS DISEASES, Epidemiology < INFECTIOUS DISEASES, Paediatric infectious disease & immunisation < PAEDIATRICS

SCHOLARONE™
Manuscripts

I, the Submitting Author has the right to grant and does grant on behalf of all authors of the Work (as defined in the below author licence), an exclusive licence and/or a non-exclusive licence for contributions from authors who are: i) UK Crown employees; ii) where BMJ has agreed a CC-BY licence shall apply, and/or iii) in accordance with the terms applicable for US Federal Government officers or employees acting as part of their official duties; on a worldwide, perpetual, irrevocable, royalty-free basis to BMJ Publishing Group Ltd ("BMJ") its licensees and where the relevant Journal is co-owned by BMJ to the co-owners of the Journal, to publish the Work in this journal and any other BMJ products and to exploit all rights, as set out in our licence.

The Submitting Author accepts and understands that any supply made under these terms is made by BMJ to the Submitting Author unless you are acting as an employee on behalf of your employer or a postgraduate student of an affiliated institution which is paying any applicable article publishing charge ("APC") for Open Access articles. Where the Submitting Author wishes to make the Work available on an Open Access basis (and intends to pay the relevant APC), the terms of reuse of such Open Access shall be governed by a Creative Commons licence – details of these licences and which Creative Commons licence will apply to this Work are set out in our licence referred to above.

Other than as permitted in any relevant BMJ Author's Self Archiving Policies, I confirm this Work has not been accepted for publication elsewhere, is not being considered for publication elsewhere and does not duplicate material already published. I confirm all authors consent to publication of this Work and authorise the granting of this licence.

For peer review only

Antibiotic use attributable to specific etiologies of diarrhea in children under two years of age in low-resource settings

Stephanie A Brennhofer, MPH[1]; James A Platts-Mills, MD[1]; Joseph A Lewnard, PhD[2]; Jie Liu, PhD[1]; Eric R Houpt, MD[1]; Elizabeth T Rogawski McQuade, PhD[1,3]

[1] Division of Infectious Diseases & International Health, School of Medicine, University of Virginia, 345 Crispell Drive, Charlottesville, VA, 22908, USA;

[2] Division of Epidemiology, School of Public Health, University of California - Berkeley, 2121 Berkeley Way, Berkeley, California, 94720, USA;

[3] Department of Epidemiology, Rollins School of Public Health, Emory University, 1518 Clifton Road NE, Atlanta, GA, 30322, USA

Correspondence to: Dr. Elizabeth T Rogawski McQuade; erogaws@emory.edu

For peer review only

ABSTRACT

Objective: Antibiotic treatment for diarrhea among children in low-resource settings is common despite guidelines that recommend limiting treatment to dysentery. Enteric vaccines and other pathogen-specific interventions may reduce selection for antimicrobial resistance (AMR) by preventing diarrhea episodes that prompt antibiotic treatment. We quantified the frequency of antibiotic treatments attributable to specific enteric pathogens in the MAL-ED birth cohort study to describe the burden of antibiotic use that could be prevented by pathogen-specific interventions like vaccines.

Design: We analyzed 9,392 reported diarrhea episodes, including 6,677 with molecular diagnostic test results, as well as 31,408 non-diarrheal stools from 1,715 children aged 0-2 years. We estimated incidence rates and the proportions of antibiotic use for diarrhea and for all indications attributable to the top ten etiologies of diarrhea. We estimated associations between specific etiologies and antibiotic treatment, and assessed whether clinical characteristics of the diarrhea episodes mediated these relationships.

Results: *Shigella* and rotavirus were the leading causes of antibiotic treatment, responsible for 11.7% and 8.6% of diarrhea treatments and 14.8 and 10.9 courses per 100 child-years, respectively. *Shigella* and rotavirus-attributable diarrhea episodes were 46% (RR:1.46; CI:1.33-1.60), and 19% (RR:1.19; CI:1.09-1.31) more likely to be treated with antibiotics, respectively, compared to other etiologies. Considering antibiotic uses for all indications, these two pathogens accounted for 5.5% of all antibiotic courses, 19.4% of all fluoroquinolone courses, and 9.6% of all macrolide courses. Among indicated treatments for dysentery, *Shigella* and *C. jejuni/C. coli*

For peer review only - http://bmjopen.bmj.com/site/about/guidelines.xhtml

were responsible for 27.5% and 8.5% of treated episodes, respectively.

Conclusion: The evidence that *Shigella* and rotavirus were disproportionately responsible for antibiotic use due to their high burden and severity further strengthens the value of interventions targeted to these pathogens. Interventions against *Campylobacter* could further prevent a large burden of indicated antibiotic treatment for dysentery, which could not be averted by antibiotic stewardship interventions.

For peer review only

For peer review only - http://bmjopen.bmj.com/site/about/guidelines.xhtml

SUMMARY BOX

Strengths and limitations of this study?

- The multi-site birth cohort design of this study with intensive twice-weekly home visits allowed capture of all antibiotic exposures for any indication including instances where antibiotics were obtained without prescriptions.
- The use of quantitative molecular diagnostics for a broad range of enteric pathogens allowed us to appropriately assign etiology to diarrhea episodes prompting antibiotic treatment.
- A limitation was that the indication for antibiotic use was not known and was therefore inferred by the overlap between treatment and diarrhea symptoms.

For peer review only

For peer review only - http://bmjopen.bmj.com/site/about/guidelines.xhtml

INTRODUCTION

Diarrhea is a major cause of antibiotic treatment among children, especially in low and middle income countries (LMICs), because of both the high incidence of diarrhea and frequency of treatment. In the multi-site Etiology, Risk Factors, and Interactions of Enteric Infections and Malnutrition and the Consequences for Child Health and Development (MAL-ED) birth cohort study, the incidence of diarrhea during the first two years of life was 273.8 episodes per 100 child years,[1] and 46% of episodes were treated with antibiotics.[2] Less than 5% of episodes were dysenteric and therefore met antibiotic treatment guidelines from the World Health Organization (WHO).[3] Nearly half of non-bloody diarrheal episodes were treated, representing a large burden of inappropriate antibiotic use.[2] Similarly, in the Global Enterics Multicenter Study (GEMS), a seven-site case-control study of moderate-to-severe diarrhea, nearly 75% of non-bloody moderate-to-severe diarrhea episodes were treated with antibiotics among children under five.[4] Frequent antibiotic treatment of diarrhea directly contributes to the development of antimicrobial resistance (AMR) for bacterial diarrheal pathogens, particularly *Shigella* and *Campylobacter*, which are on the WHO priority pathogen list for concern about AMR.[5] Treatment of diarrhea also affects AMR more broadly through antibiotic selection pressure to bacteria carried at the time of treatment.

Because there is uncontrolled access to antibiotics in many LMICs, children often receive antibiotics without seeking care.[6] Even if a child presents to care, clinical predictors and point-of-care diagnostics to identify diarrhea episodes that could respond to antibiotics are largely unavailable.[7] Prescribing antibiotics for diarrhea remains the standard of care in many settings despite the recognized need for antibiotic stewardship and guidelines to reserve antibiotic

For peer review only - http://bmjopen.bmj.com/site/about/guidelines.xhtml

treatment for dysentery.[8] Vaccines or other interventions that prevent diarrheal illnesses from occurring and therefore prompting treatment might provide the most effective mechanism for reducing antibiotic use.[9,10]

Influenza and pneumococcal conjugate vaccines have been found to reduce antibiotic use through the prevention of respiratory illnesses.[11] A recent randomized controlled trial demonstrated that maternal respiratory syncytial virus (RSV) vaccination prevented 13% of antibiotic use in the first three months of life.[12] Additionally, rotavirus vaccination was estimated to prevent 13.6 million antibiotic-treated diarrhea episodes annually among children under two years in LMICs.[13] Estimation of the further reductions in antibiotic use that could be achieved by vaccines against enteric pathogens such as *Shigella*, enterotoxigenic *Escherichia coli* (ETEC), *Campylobacter*, and *Cryptosporidium* appropriately broadens the vaccine value proposition and could inform priority-setting for the development, evaluation, and implementation of these interventions.[14]

To estimate the preventable burden of antibiotic use for diarrhea that could be achieved by vaccines or other pathogen-specific interventions, we quantified the amount of antibiotic use that could be attributed to the treatment of specific causes of diarrhea in the MAL-ED birth cohort study.

METHODS

Study design and participants

The study design for MAL-ED has been described elsewhere.[15] Briefly, this study was conducted

For peer review only - http://bmjopen.bmj.com/site/about/guidelines.xhtml

from November 2009 to February 2014, and participants were enrolled at eight sites: Dhaka, Bangladesh; Fortaleza, Brazil; Vellore, India; Bhaktapur, Nepal; Loreto, Peru; Naushero Feroze, Pakistan; Venda, South Africa; and Haydom, Tanzania. Children were followed from birth (<17 days of age) through age 24 months. Fieldworkers conducted twice weekly home visits in which they collected information on antibiotic drug classes given to the child and diarrhea since the last home visit. Diarrhea was defined as three or more loose stools in a 24-hour period or visible blood in at least one stool. Diarrheal episodes were separated by at least two days without diarrhea. Stool samples were collected during diarrhea and monthly in the absence of diarrhea. Episode severity was defined by a modified Vesikari score, previously described.[16] Dysentery was defined as reported presence of blood in at least one stool during a diarrheal episode. Antibiotic courses for diarrhea were identified when antibiotic use was reported on any day during a diarrhea episode. Distinct antibiotic courses not associated with diarrhea were defined if separated by at least two days of no antibiotic use, as previously.[2]

Stool testing

Pathogens were detected among all stool samples collected from children with complete follow-up. To extract total nucleic acid, the QIAmp Fast DNA Stool Mini Kit (Qiagen) was used.[17] Quantitative polymerase chain reaction (qPCR) using AgPath One Step realtime PCR kit (Thermo-Fisher) was used to detect 29 enteropathogens via the TaqMan Array Card (TAC) platform.[1] A quantification cycle (Cq) threshold of 35 was the analytic limit of detection. Ten enteric pathogens that were previously identified as the top causes of diarrhea in MAL-ED[1] were included in these analyses: adenovirus 40/41, astrovirus, *Campylobacter jenjui/Campylobacter coli* (*C. jejuni/C.coli*), *Cryptosporidium*, norovirus, rotavirus, sapovirus, *Shigella*, typical

For peer review only - http://bmjopen.bmj.com/site/about/guidelines.xhtml

enteropathogenic *Escherichia coli* (tEPEC), and heat stable enterotoxigenic *Escherichia coli* (ST-ETEC).

Data analysis

To identify the pathogens responsible for diarrhea treated with antibiotics, we calculated pathogen-specific attributable fractions (AF) of antibiotic-treated diarrhea using generalized linear mixed-effects models (GLMM) that associated pathogen quantity detected with presence in diarrheal versus non-diarrheal stools. The model included sex, test batch, age in quarters, pathogen quantity, pathogen quantity squared, an interaction between pathogen quantity and age, the quantity of the other nine pathogens, a random intercept for individual, and a random slope for site. We calculated episode-specific pathogen attributable fractions as $AFe_i = 1 - (1/\mathrm{OR}e_i)$, where $\mathrm{OR}e$ is the pathogen- and quantity-specific odds ratio from the GLMM. Population-level AFs were calculated by summing the attributable fractions per episode (AFes) across all antibiotic-treated episodes, j, i.e. $\left(\frac{1}{j}\right) * \sum_{i=1}^{j} AFe_i$.

We calculated attributable incidence (AI) rates of antibiotic use for each pathogen per 100 child-years as the product of the AF and the total incidence of antibiotic courses for diarrhea identified by surveillance. We also calculated the proportion of all antibiotic use that was attributable to each pathogen as the product of the AF and the proportion of all antibiotic courses that were given for diarrhea. To quantify appropriate antibiotic use, we calculated the proportion of pathogen-attributable antibiotic use that was for dysentery. All results were stratified by age, site, and antibiotic drug class.

For peer review only - http://bmjopen.bmj.com/site/about/guidelines.xhtml

To assess whether specific pathogens were associated with antibiotic treatment, we estimated risk ratios (RR) for the association between specific pathogens and antibiotic treatment using the pathogen-specific AFe as a continuous exposure. We used the Poisson approximation for log-binomial regression with generalized estimating equations (GEE) to account for repeated episodes within each child. Estimates were scaled to represent the difference between complete attribution (AFe = 1, or the maximum observed AFe for that pathogen if <1) and no attribution. Estimates were adjusted for site, age as a quadratic spline, sex, and the Water, Assets, Maternal Education, Income (WAMI) index, a measure of socioeconomic status.[18]

To further assess whether diarrhea severity mediated the associations with antibiotic treatment, we estimated the total effects of *Shigella* and rotavirus on antibiotic treatment, the pure natural direct effects (PNDE), the total natural indirect effects (TNIE) through the diarrhea severity score and dysentery (*Shigella* only), and the proportions mediated by diarrhea severity and dysentery using the inverse odds ratio weighting approach to mediation analysis with weights truncated at the top 1%.[19,20] The TNIE is the magnitude of the effect of each pathogen on antibiotic use that can be explained by the association of the pathogen with diarrhea severity, while the PNDE describes the remainder of the effect that is not mediated by severity. For the mediation analysis, etiologies were assigned if the pathogen AFe was ≥ 0.5 (i.e. majority attribution). For all analyses, 95% confidence intervals (CI) were estimated by bootstrap with 1,000 resamples.

Research ethics approval statement

This study received approval from the UVA IRB-HSR: 14595.

For peer review only - http://bmjopen.bmj.com/site/about/guidelines.xhtml

Patient and public involvement

It was not possible to involve patients or the public in the design, conduct, reporting, or dissemination plans as this was a secondary data analysis of a study conducted in 2009-2014.

RESULTS

These analyses included 1,715 children with 9,392 reported diarrheal episodes and 38,085 (n=6,677 diarrheal, n=31,408 non-diarrheal) stool samples with valid qPCR results for the ten pathogens included (Table 1). Caregivers reported 15,670 antibiotic courses, among which 4,335 courses were associated with treatment of diarrhea. The overall incidence of antibiotic use due to diarrhea was 126.4 courses per 100 child-years, and incidence was higher during the first year of life (134.46 courses per 100 child-years) than the second (118.31 courses per 100 child-years). Higher incidence in younger children reflects higher diarrhea incidence overall, despite a lower proportion of episodes treated with antibiotics in the first year (n=2199/5015, 44.1%) compared to the second year (n=2136/4377, 48.7%). Episodes of dysentery accounted for a small proportion of diarrhea episodes (n=461, 4.9%) and antibiotic courses for diarrhea (n=345, 8.0%), despite the fact that 75% of dysentery episodes were treated.

Shigella had the highest incidence of antibiotic use of 14.77 (95% CI: 13.25-16.84) courses per 100 child-years, followed by rotavirus (10.90, 95% CI: 9.75-12.42), sapovirus (10.24, 95% CI: 8.37-12.55), adenovirus 40/41 (9.63, 95% CI: 8.27-11.69), and ST-ETEC (8.56, 95% CI: 7.04-10.71) (Figure 1A, Table S1). *Shigella* was the leading cause of all classes of antibiotic use, except for penicillins, for which attribution was more evenly split across pathogens. Proportionally, *Shigella* and rotavirus were responsible for 11.7% (10.5-13.3) and 8.6% (7.7-9.8)

For peer review only - http://bmjopen.bmj.com/site/about/guidelines.xhtml

of antibiotic treatments for diarrheal episodes, respectively (Figure 2A, Table S2). These two pathogens were responsible for an even larger total proportion of fluoroquinolone (33.0%) and macrolide (28.0%) use for diarrhea.

The amount of antibiotic use attributed to specific pathogens varied widely across sites, with more frequent pathogen-attributable use in the South Asian sites compared to African sites. *Shigella* was the leading cause of antibiotic use in India, Nepal, Peru, Pakistan, and South Africa. In contrast, sapovirus was the leading cause in Brazil and Peru, adenovirus 40/41 was the leading cause in Bangladesh, and ST-ETEC was the leading cause in Tanzania (Tables S3 and S4). Bangladesh was an outlier in terms of frequency; adenovirus 40/41 and *Shigella* were responsible for 50.99 (95% CI: 42.72-62.14) and 45.79 (95% CI: 39.70-54.61) courses per 100 child-years at this site alone, respectively (Figure 1B; Table S5). Of note, while Pakistan had a higher incidence of antibiotic use for diarrhea overall (373.37 per 100 child-years) than Bangladesh (213.57 per 100 child-years), many episodes in Pakistan could not be attributed to the pathogens studied. Rotavirus accounted for a lower proportion of pathogen-attributable antibiotic treatments in Brazil, Peru, and South Africa compared to the other sites (Table S3).

Causes of antibiotic treatment also varied by age. In the first year of life, the pathogens responsible for the highest incidence of antibiotic treatment were rotavirus, adenovirus 40/41, sapovirus, and norovirus, despite antibiotic use being inappropriate for the viral pathogens (Figure S1, Table S6). In the second year of life, the incidence of antibiotic use for *Shigella* was nearly twice that of any other single pathogen.

For peer review only - http://bmjopen.bmj.com/site/about/guidelines.xhtml

Diarrhea was the indication for approximately one-quarter (27.7%) of antibiotic treatments overall. Therefore, specific enteric pathogens were responsible for a lower proportion of all antibiotic exposures for any indication. Overall, 3.2% and 2.4% of all antibiotic courses given were attributable to *Shigella* and rotavirus, respectively (Figure 2B; Table S7). Both were responsible for a substantial proportion of treatments with specific antibiotic drug classes. 12.2% and 5.5% of fluoroquinolones and macrolides, respectively, were used for treatment of *Shigella*, and 7.1% and 4.0% of fluoroquinolones and macrolides, respectively, were used for treatment of rotavirus. All other pathogens were each responsible for approximately 2% or less of all antibiotic treatments.

Focusing on indicated antibiotic treatments, the highest proportions of antibiotic use for dysentery were attributed to *Shigella* (27.5%) and *C. jejuni/C. coli* (8.5%), respectively (Table S8). These two pathogens accounted for a larger proportion of antibiotic treated dysentery episodes compared to antibiotic treated watery diarrhea episodes (17.2% and 5.3% more, respectively). However, less than a fifth of all antibiotic treatments attributable to *Shigella* (18.7%) and *C. jejuni/C. coli* (18.6%) were for dysentery. The attributable fractions of antibiotic treatments for dysentery compared to watery diarrhea did not differ for the other pathogens, and less than 10% of antibiotic treatments attributed to the other pathogens were for the treatment of dysentery.

After adjustment for age, site, sex, and socioeconomic status, *Shigella*-attributable diarrhea episodes were 46% more likely to be treated with antibiotics compared to all other episodes (adjusted risk ratio (aRR): 1.46, 95% CI: 1.33-1.60), and rotavirus-attributable episodes were

For peer review only - http://bmjopen.bmj.com/site/about/guidelines.xhtml

19% more likely to be treated (1.19, 1.09-1.31) (Figure 3). The associations were stronger for key drug classes; *Shigella*-attributable diarrhea episodes were 49% more likely to be treated with fluoroquinolones or macrolides compared to other episodes (1.49, 1.28-1.73), and rotavirus-attributable episodes were 21% more likely to be treated (1.21, 1.04-1.41). The associations between *Shigella* and rotavirus and antibiotic treatment were consistent across most sites, excluding Tanzania and Nepal (Table S9). Uniquely, *Cryptosporidium* was strongly associated with antibiotic treatment in Tanzania (aRR: 3.18, 1.36-7.43) and India (aRR: 2.11, 1.18-3.79).

Diarrhea severity and dysentery mediated 5% and 18% of the association between antibiotic treatment and *Shigella*, respectively (Table S10). When considered together, these two factors mediated a total 26% of the antibiotic treatment association and 48% of the fluoroquinolone and macrolide treatment association with *Shigella* (Table 2). Similarly, diarrhea severity mediated 44% of the association between rotavirus and antibiotic treatment and 53% of the association with fluoroquinolone and macrolide treatment.

DISCUSSION

Because diarrhea was responsible for more than a quarter of antibiotic treatments in the MAL-ED study, interventions that target specific enteric pathogens could reduce antibiotic selection pressure and make an important contribution to efforts to combat AMR. We found that *Shigella* and rotavirus were the top causes of antibiotic treatment for diarrhea, with more than two in every ten children on average exposed to antibiotics due to each of these pathogens in the first two years of life. Furthermore, *Shigella* was responsible for the most uses of fluoroquinolones and macrolides, which are first line therapies for *Campylobacter, Shigella,* and diarrheagenic *E.*

For peer review only - http://bmjopen.bmj.com/site/about/guidelines.xhtml

coli. While the frequency of antibiotic treatment varied by an order of magnitude across settings, *Shigella* and rotavirus were among the leading causes at all sites. Notably, rotavirus was a less frequent cause of antibiotic use in the three sites that had introduced rotavirus vaccine prior to the study.

These results are consistent with a similar analysis of facility-ascertained moderate-to-severe diarrhea conducted in GEMS.[4] but have broader implications since they include antibiotic treatments for diarrhea episodes identified in the community and therefore report much higher rates of antibiotic treated diarrhea. In LMICs, where the majority of antibiotic use occurs outside of medically attended care, estimates of antibiotic use from healthcare settings alone are large underestimates of the total burden. This analysis also provides a broader context by considering antibiotic treatments for all indications beyond diarrhea, which is important for LMIC settings which have high burdens of respiratory illnesses and other infections as well.

The contribution of most enteric pathogens to antibiotic use was in proportion to their contribution to diarrhea overall. However, in addition to being the leading causes of diarrhea in the first and second years of life, respectively, rotavirus and *Shigella* were disproportionately more responsible for antibiotic use than would have been expected based on the age-specific incidence of disease. Because point-of-care diagnostics were not available, treatment decisions were not made based on known etiology but were rather likely due to unique features of the clinical syndromes caused by these pathogens. Indeed, we found evidence that the associations between *Shigella* and rotavirus and antibiotic treatment could be explained by the fact that these pathogens cause more severe disease. Unsurprisingly, since *Shigella* is the leading cause of

For peer review only - http://bmjopen.bmj.com/site/about/guidelines.xhtml

dysentery for which treatment is recommended, dysentery also mediated the relationship between *Shigella* and antibiotic use. Because diarrhea severity and dysentery only explained a portion of the relationships, there may be other subjective indicators for treatment that were insufficiently captured by the severity metrics captured.

While the contribution of individual enteric pathogens to total antibiotic use was limited (<5% for each pathogen), reductions of these magnitudes would be comparable or larger than the effect of most existing antibiotic stewardship interventions.[21] Furthermore, the attributable proportions increased considerably for fluoroquinolones and macrolides, which are the first-line classes for diarrhea treatment and important oral antibiotic options for a broad range of community-acquired infections. For example, *Shigella* was responsible for approximately 1 in 8 uses of fluoroquinolones and 1 in 18 uses of macrolides. *Shigella* vaccines in development[22,23] could provide an opportunity to reduce this use. Importantly, enteric viruses accounted for a quarter of all fluoroquinolone use and 16% of macrolide use. These treatment courses were not indicated and represent the burden of antibiotic overuse that could be potentially prevented by vaccines or other pathogen-specific interventions.

Interventions that reduce the incidence of bacterial diarrhea episodes requiring antibiotics, particularly due to *Shigella* and *Campylobacter,* would also have the direct benefit of potentially preventing antibiotic-resistant disease. *Shigella* and *Campylobacter* are on the WHO priority pathogens list for research and development of new antibiotics due to increasing AMR [24]. While antibiotic resistance testing was not conducted in MAL-ED, some of the treated episodes may have been resistant to fluoroquinolones and/or macrolides, as has been reported particularly in

For peer review only - http://bmjopen.bmj.com/site/about/guidelines.xhtml

Asia.[24,25]

Finally, because subclinical carriage of these and other bacterial enteropathogens is highly common among young children in LMICs,[26] reductions in antibiotic use overall, including treatments of viral diarrhea, would have the important ancillary benefit of preventing antibiotic exposure to bacteria present as subclinical infections. This type of antibiotic exposure has been described as "bystander selection," or the selective pressure for resistance on pathogens that are not the target of treatment.[27] *Shigella* and *Campylobacter* were detected in 10% and 28% of all non-diarrheal stools collected in MAL-ED[26], respectively, suggesting that these pathogens were likely frequently exposed to antibiotics due to diarrhea treatment.

Because prescriptions and/or caregiver-reported indications for treatment were unavailable, this analysis was limited by attributing antibiotic use to diarrhea based on the temporal overlap of symptoms. Furthermore, information on specific drug given and dosing were not available, and antibiotic courses were defined based on antibiotic-free days rather than the intended duration.

The evidence that *Shigella* and rotavirus were disproportionately responsible for antibiotic use due to their high burden and severity strengthens the value proposition for rotavirus and *Shigella* vaccines[10] and other pathogen-specific interventions. Prevention of diarrheal disease offers an important opportunity to reduce both antibiotic use and overuse.

For peer review only - http://bmjopen.bmj.com/site/about/guidelines.xhtml

DECLARATIONS

Author affiliations

[1] Division of Infectious Diseases & International Health, School of Medicine, University of Virginia, 345 Crispell Drive, Charlottesville, VA, 22908, USA;

[2] Division of Epidemiology, School of Public Health, University of California - Berkeley, 2121 Berkeley Way, Berkeley, California, 94720, USA;

[3] Department of Epidemiology, Rollins School of Public Health, Emory University, 1518 Clifton Road NE, Atlanta, GA, 30322, USA

For peer review only

Contributors

SAB led data analysis, interpretation, visualization, and writing of the report. JAP-M led and conceptualized the data analysis and contributed to the interpretation, and reviewing/editing the report. JAL contributed to interpretation, and reviewing/editing the report. JL led the development of the laboratory assays and contributed to reviewing/editing the report. ERH led funding acquisition and administration of the parent study, and contributed to reviewing/editing the report. ETRM led the conceptualization, methodology, funding acquisition, writing of the report and contributed to data analysis, interpretation, and visualization. All authors read and approved the final manuscript.

Funding

This work was supported by Wellcome (219741/Z/19/Z to ETRM). The Etiology, Risk Factors and Interactions of Enteric Infections and Malnutrition and the Consequences for Child Health and Development Project (MAL-ED) was a collaborative project supported by the Bill &

For peer review only - http://bmjopen.bmj.com/site/about/guidelines.xhtml

Melinda Gates Foundation (OPP1131125), the Foundation for the NIH, the National Institutes of Health, and the Fogarty International Center.

Competing interests

The authors declare that they have no competing interests.

Patient consent for publication

Not required.

Data availability statement

De-identified participant data from the MAL-ED study is publicly available at ClinEpiDB.org after approval of a proposal by the study PIs.

For peer review only

REFERENCES

1. Platts-Mills JA, Liu J, Rogawski ET, et al. Use of quantitative molecular diagnostic methods to assess the aetiology, burden, and clinical characteristics of diarrhoea in children in low-resource settings: a reanalysis of the MAL-ED cohort study. *The Lancet Global Health*. 2018;6(12):e1309-e1318. doi:10.1016/S2214-109X(18)30349-8

2. Rogawski ET, Platts-Mills JA, Seidman JC, et al. Use of antibiotics in children younger than two years in eight countries: a prospective cohort study. *Bulletin of the World Health Organization*. 2017;95(1):49-61.

3. World Health Organization. *WHO Recommendations on the Management of Diarrhoea and Pneumonia in HIV-Infected Infants and Children: Integrated Management of Childhood Illness (IMCI)*. World Health Organization; 2010. Accessed May 4, 2021. https://www.ncbi.nlm.nih.gov/books/NBK305340/

4. Lewnard JA, McQuade ETR, Platts-Mills JA, Kotloff KL, Laxminarayan R. Incidence and etiology of clinically-attended, antibiotic-treated diarrhea among children under five years of age in low- and middle-income countries: Evidence from the Global Enteric Multicenter Study. *PLOS Neglected Tropical Diseases*. 2020;14(8):e0008520. doi:10.1371/journal.pntd.0008520

5. World Health Organization. WHO publishes list of bacteria for which new antibiotics are urgently needed. Published February 27, 2017. Accessed June 4, 2021. https://www.who.int/news/item/27-02-2017-who-publishes-list-of-bacteria-for-which-new-antibiotics-are-urgently-needed

6. Abdulah R. Antibiotic Abuse in Developing Countries. *Pharmaceutical Regulatory Affairs: Open Access*. 2012;1:e106. doi:10.4172/2167-7689.1000e106

7. Bebell LM, Muiru AN. Antibiotic Use and Emerging Resistance: How Can Resource-Limited Countries Turn the Tide? *Global Heart*. 2014;9(3):347-358. doi:10.1016/j.gheart.2014.08.009

8. Mittal SK, Mathew JL. Regulating the Use of Drugs in Diarrhea. *Journal of Pediatric Gastroenterology and Nutrition*. 2001;33:S26.

9. Jansen KU, Knirsch C, Anderson AS. The role of vaccines in preventing bacterial antimicrobial resistance. *Nat Med*. 2018;24(1):10-19. doi:10.1038/nm.4465

10. Lipsitch M, Siber GR. How Can Vaccines Contribute to Solving the Antimicrobial Resistance Problem? *mBio*. 2016;7(3):e00428-16. doi:10.1128/mBio.00428-16

11. Buckley BS, Henschke N, Bergman H, et al. Impact of vaccination on antibiotic usage: a systematic review and meta-analysis. *Clinical Microbiology and Infection*. 2019;25(10):1213-1225. doi:10.1016/j.cmi.2019.06.030

For peer review only - http://bmjopen.bmj.com/site/about/guidelines.xhtml

12. Lewnard JA, Fries LF, Cho I, Chen J, Laxminarayan R. Prevention of antimicrobial prescribing among infants following maternal vaccination against respiratory syncytial virus. *medRxiv*. Published online in clearance 2021.

13. Lewnard JA, Lo NC, Arinaminpathy N, Frost I, Laxminarayan R. Childhood vaccines and antibiotic use in low- and middle-income countries. *Nature*. 2020;581(7806):94-99. doi:10.1038/s41586-020-2238-4

14. Cohen D, Muhsen K. Vaccines for enteric diseases. *Human Vaccines & Immunotherapeutics*. 2019;15(6):1205-1214. doi:10.1080/21645515.2019.1611200

15. The MAL-ED Network Investigators. The MAL-ED Study: A Multinational and Multidisciplinary Approach to Understand the Relationship Between Enteric Pathogens, Malnutrition, Gut Physiology, Physical Growth, Cognitive Development, and Immune Responses in Infants and Children Up to 2 Years of Age in Resource-Poor Environments. *Clinical Infectious Diseases*. 2014;59(suppl_4):S193-S206. doi:10.1093/cid/ciu653

16. Platts-Mills JA, Babji S, Bodhidatta L, et al. Pathogen-specific burdens of community diarrhoea in developing countries: a multisite birth cohort study (MAL-ED). *The Lancet Global Health*. 2015;3(9):e564-e575.

17. Liu J, Kabir F, Manneh J, et al. Development and assessment of molecular diagnostic tests for 15 enteropathogens causing childhood diarrhoea: a multicentre study. *The Lancet Infectious Diseases*. 2014;14(8):716-724. doi:10.1016/S1473-3099(14)70808-4

18. Psaki SR, Seidman JC, Miller M, et al. Measuring socioeconomic status in multicountry studies: results from the eight-country MAL-ED study. *Popul Health Metr*. 2014;12(1):8. doi:10.1186/1478-7954-12-8

19. Shi B, Choirat C, Valeri L. CMAverse: a suite of functions for causal mediation analysis. https://bs1125.github.io/CMAverse/

20. Nguyen QC, Osypuk TL, Schmidt NM, Glymour MM, Tchetgen Tchetgen EJ. Practical guidance for conducting mediation analysis with multiple mediators using inverse odds ratio weighting. *Am J Epidemiol*. 2015;181(5):349-356. doi:10.1093/aje/kwu278

21. Hallsworth M, Chadborn T, Sallis A, et al. Provision of social norm feedback to high prescribers of antibiotics in general practice: a pragmatic national randomised controlled trial. *The Lancet*. 2016;387(10029):1743-1752. doi:10.1016/S0140-6736(16)00215-4

22. Riddle MS, Chen WH, Kirkwood CD, MacLennan CA. Update on vaccines for enteric pathogens. *Clinical Microbiology and Infection*. 2018;24(10):1039-1045. doi:10.1016/j.cmi.2018.06.023

23. Talaat KR, Alaimo C, Martin P, et al. Human challenge study with a Shigella bioconjugate vaccine: Analyses of clinical efficacy and correlate of protection. *EBioMedicine*. 2021;66:103310. doi:10.1016/j.ebiom.2021.103310

For peer review only - http://bmjopen.bmj.com/site/about/guidelines.xhtml

24. Tacconelli E, Magrini N. Global priority list of antibiotic-resistant bacteria to guide research, discovery, and development of new antibiotics. Published April 8, 2021. https://www.who.int/medicines/publications/WHO-PPL-Short_Summary_25Feb-ET_NM_WHO.pdf

25. Gu B, Cao Y, Pan S, et al. Comparison of the prevalence and changing resistance to nalidixic acid and ciprofloxacin of Shigella between Europe-America and Asia-Africa from 1998 to 2009. *Int J Antimicrob Agents*. 2012;40(1):9-17. doi:10.1016/j.ijantimicag.2012.02.005

26. Rogawski ET, Liu J, Platts-Mills JA, et al. Use of quantitative molecular diagnostic methods to investigate the effect of enteropathogen infections on linear growth in children in low-resource settings: longitudinal analysis of results from the MAL-ED cohort study. *The Lancet Global Health*. 2018;6(12):e1319-e1328. doi:10.1016/S2214-109X(18)30351-6

27. Tedijanto C, Olesen SW, Grad YH, Lipsitch M. Estimating the proportion of bystander selection for antibiotic resistance among potentially pathogenic bacterial flora. *PNAS*. 2018;115(51):E11988-E11995.

For peer review only

For peer review only - http://bmjopen.bmj.com/site/about/guidelines.xhtml

Table 1: Antibiotic use, treatment of diarrhea, and stool sample collection among 1715 children enrolled in the MAL-ED cohort

	Dhaka, Bangladesh	Fortaleza, Brazil	Vellore, India	Bhaktapur, Nepal	Loreto, Peru	Naushero Feroze, Pakistan	Venda, South Africa	Haydom, Tanzania	Overall
Children included[a]	210	165	227	227	194	246	237	209	1715
Total antibiotic courses	3695	224	1740	1059	2041	4922	508	1481	15670
Surveilled diarrheal episodes	1520	168	960	1060	1742	3110	295	537	9392
Antibiotic treatments for diarrhea episodes[b]	897 (59.0%)	18 (10.7%)	242 (25.2%)	319 (30.1%)	688 (39.5%)	1837 (59.1%)	62 (21.0%)	272 (50.7%)	4335 (46.2%)
Penicillin treatment[b]	133 (8.8%)	7 (4.2%)	55 (5.7%)	60 (5.7%)	150 (8.6%)	287 (9.2%)	32 (10.8%)	99 (18.4%)	823 (8.8%)
Sulfonamide treatment[b]	2 (0.1%)	9 (5.4%)	25 (2.6%)	69 (6.5%)	195 (11.2%)	210 (6.8%)	19 (6.4%)	52 (9.7%)	581 (6.2%)
Macrolides treatment[b]	537 (35.3%)	0 (0.0%)	11 (1.1%)	31 (2.9%)	295 (16.9%)	83 (2.7%)	2 (0.7%)	13 (2.4%)	972 (10.3%)
Metronidazole treatment[b]	74 (4.9%)	2 (1.2%)	74 (7.7%)	161 (15.2%)	31 (1.8%)	1185 (38.1%)	6 (2.0%)	125 (23.3%)	1658 (17.7%)
Cephalosporin treatment[b]	77 (5.1%)	1 (0.6%)	88 (9.2%)	45 (4.2%)	33 (1.9%)	575 (18.5%)	1 (0.3%)	2 (0.4%)	822 (8.8%)
Fluoroquinolone treatment[b]	252 (16.6%)	0 (0.0%)	67 (7.0%)	30 (2.8%)	72 (4.1%)	84 (2.7%)	0 (0.0%)	2 (0.4%)	507 (5.4%)
Other antibiotic treatment[b,c]	24 (1.6%)	0 (0.0%)	46 (4.8%)	6 (0.6%)	61 (3.5%)	792 (25.5%)	8 (2.7%)	23 (4.3%)	960 (10.2%)
Surveilled dysentery episodes[b]	65 (4.3%)	4 (2.4%)	60 (6.2%)	48 (4.5%)	101 (5.8%)	101 (3.2%)	11 (3.7%)	71 (13.2%)	461 (4.9%)
Antibiotic treatments for dysentery[d]	51 (5.7%)	2 (11.1%)	27 (11.2%)	41 (12.9%)	86 (12.5%)	82 (4.5%)	4 (6.5%)	52 (19.1%)	345 (8.0%)
Diarrheal stools included in the attribution analysis[b]	1379 (90.7%)	90 (53.6%)	631 (65.7%)	904 (85.3%)	1585 (91.0%)	1815 (58.4%)	115 (39.0%)	158 (29.4%)	6677 (71.1%)
Non-diarrheal stools included in the attribution analysis[e]	3813 (84.2%)	2800 (86.4%)	4498 (88.9%)	4533 (87.8%)	3504 (81.5%)	3896 (80.0%)	4355 (80.7%)	4009 (86.1%)	31408 (84.4%)

Data are n or n (%). Diarrheal and non-diarrheal stools included in this analysis were those that were collected and validly tested for each of the 10 pathogens. [a] Children were included if they had two complete years of follow-up with qPCR data. [b] N=9392. [c] Includes reported tetracyclines, other, and unknown antibiotic use. [d] N=4335. [e] N=37216.

For peer review only

Table 2. Assessment of whether diarrhea severity and dysentery mediated the relationship between *Shigella* and rotavirus diarrhea and antibiotic treatment among 1715 children in the MAL-ED cohort

	Shigella (Mediated by diarrhea severity and dysentery)		Rotavirus (Mediated by diarrhea severity)	
	Any antibiotic	Fluoroquinolones or macrolides	Any antibiotic	Fluoroquinolones or macrolides
Total Effect Rate Ratio	1.30 (1.21, 1.40)	1.39 (1.21, 1.58)	1.10 (1.01, 1.19)	1.17 (1.01, 1.33)
Pure Natural Direct Effect Rate Ratio	1.22 (1.12, 1.34)	1.20 (1.02, 1.40)	1.06 (0.97, 1.15)	1.08 (0.89, 1.27)
Total Natural Indirect Effect Rate Ratio	1.07 (1.00, 1.12)	1.16 (1.05, 1.28)	1.04 (0.98, 1.10)	1.08 (0.96, 1.24)
Proportion Mediated	0.26 (0.02, 0.50)	0.48 (0.16, 0.90)	0.44 (0.00, 1.00)	0.53 (0.00, 1.00)

Data are risk ratios (RR) with 95% confidence intervals (CIs). Note: The Total Effect Rate Ratio for *Shigella* and rotavirus do not equal the total effects in Figure 3 as the attributable fractions per episode (AFe) were dichotomized > 0.5 for the mediation models, but left continuous in Figure 3.

For peer review only

For peer review only - http://bmjopen.bmj.com/site/about/guidelines.xhtml

Figure 1. Attributable incidence of pathogen-specific antibiotic courses for diarrhea by antibiotic drug class (A) and by site (B) among 1715 children in the MAL-ED cohort. Error bars show 95% CI. *C. jejuni/C. coli = Campylobacter jejuni/Campylobacter coli*. ST-ETEC= heat-stable enterotoxigenic *Escherichia coli*. tEPEC = typical enteropathogenic *Escherichia coli*.

Figure 2. Pathogen-specific attributable fractions of antibiotic courses for diarrhea (A) and for all indications (B) by antibiotic drug class among 1715 children in the MAL-ED cohort. Error bars show 95% CI. *C. jejuni/C. coli = Campylobacter jejuni/Campylobacter coli*. ST-ETEC= heat-stable enterotoxigenic *Escherichia coli*. tEPEC = typical enteropathogenic *Escherichia coli*.

Figure 3. Associations between specific diarrhea etiologies and treatment with any antibiotics and fluoroquinolones or macrolides among 1715 children in the MAL-ED cohort. Estimates are risk ratios adjusted for age, sex, socioeconomic status, and site. Error bars show 95% CI. *C. jejuni/C. coli = Campylobacter jejuni/Campylobacter coli*. ST-ETEC= heat-stable enterotoxigenic *Escherichia coli*. tEPEC = typical enteropathogenic *Escherichia coli*.

For peer review only

For peer review only - http://bmjopen.bmj.com/site/about/guidelines.xhtml

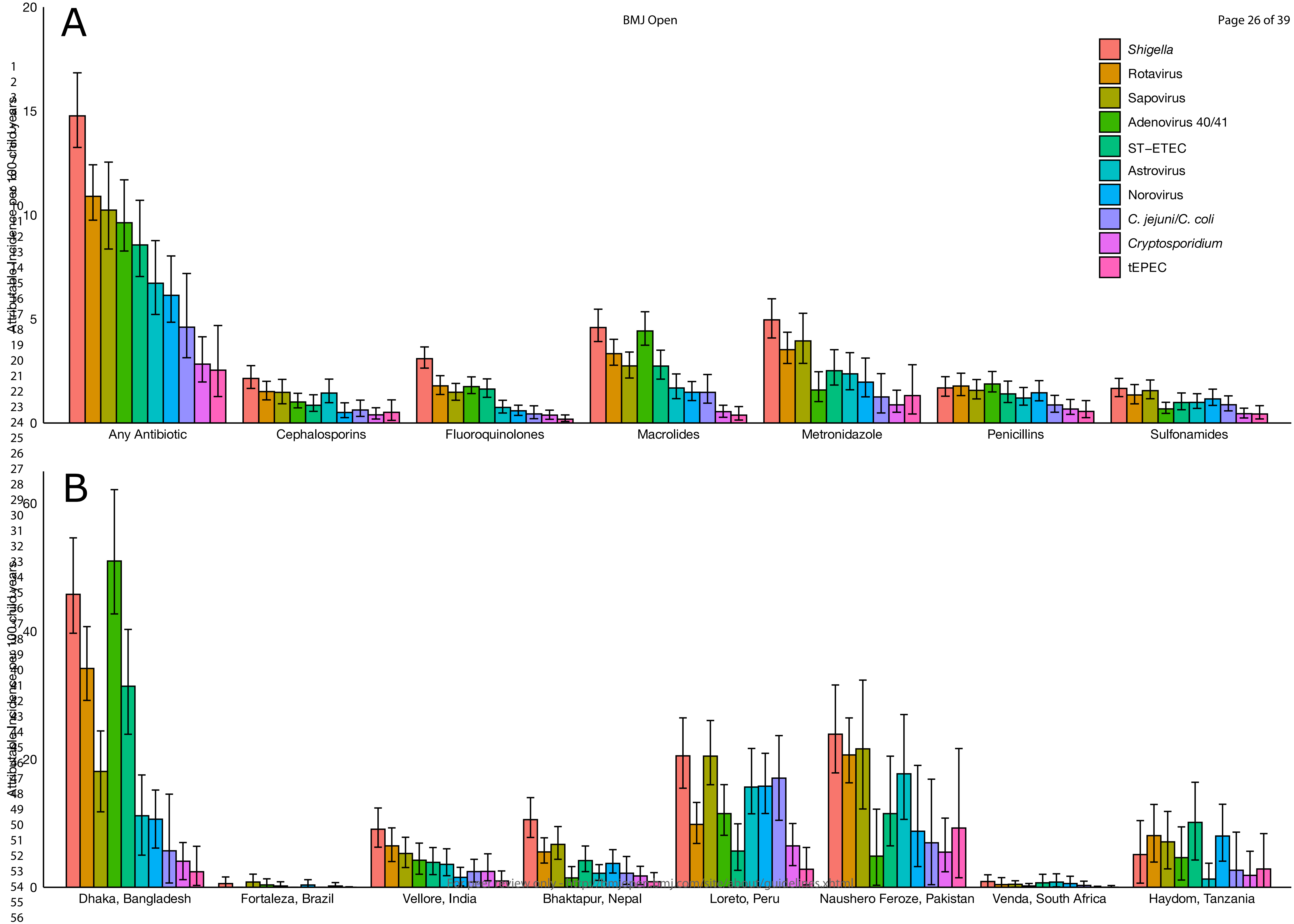

For peer review only - http://bmjopen.bmj.com/site/about/guidelines.xhtml

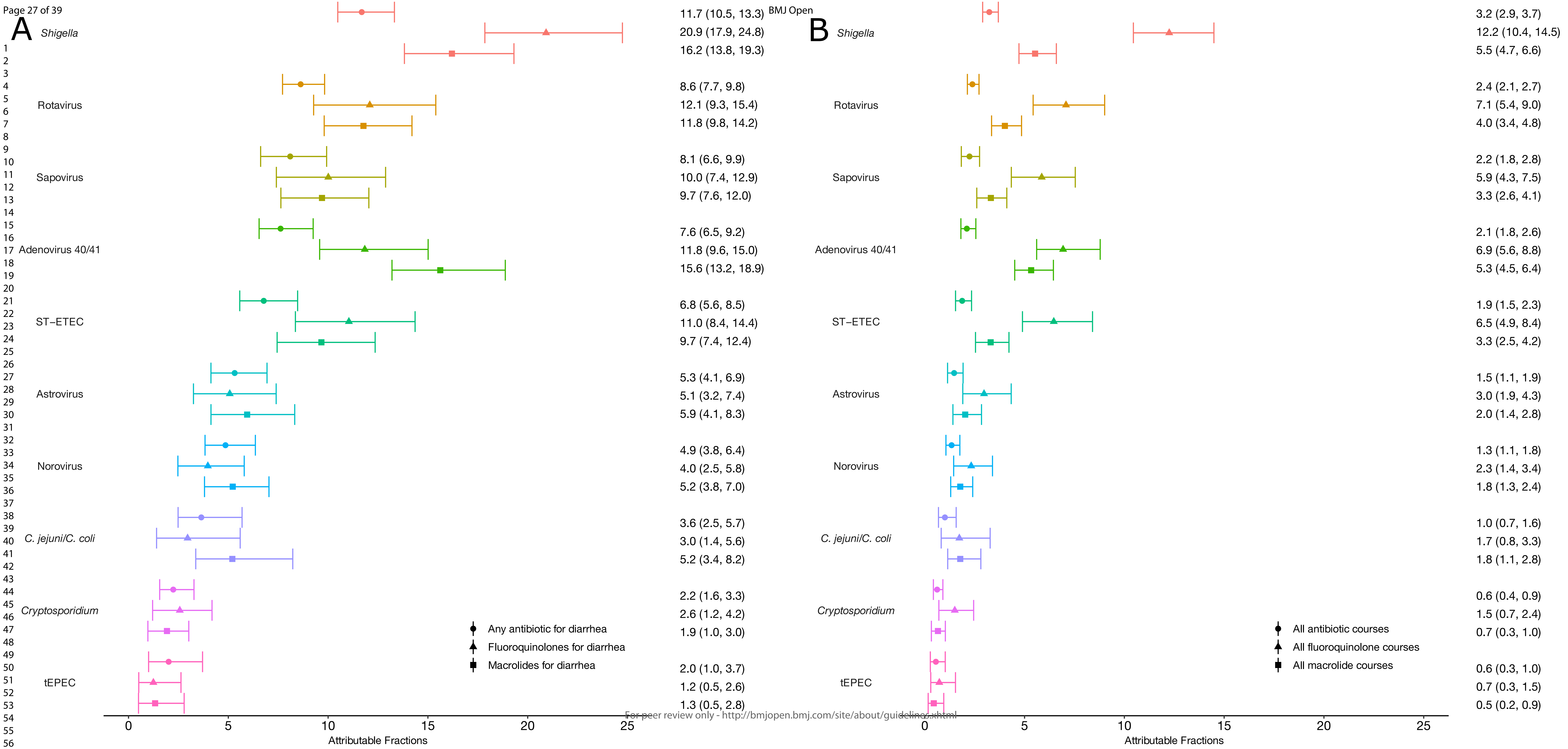
A
Shigella
11.7 (10.5, 13.3)
20.9 (17.9, 24.8)
16.2 (13.8, 19.3)
Rotavirus
8.6 (7.7, 9.8)
12.1 (9.3, 15.4)
11.8 (9.8, 14.2)
Sapovirus
8.1 (6.6, 9.9)
10.0 (7.4, 12.9)
9.7 (7.6, 12.0)
Adenovirus 40/41
7.6 (6.5, 9.2)
11.8 (9.6, 15.0)
15.6 (13.2, 18.9)
ST−ETEC
6.8 (5.6, 8.5)
11.0 (8.4, 14.4)
9.7 (7.4, 12.4)
Astrovirus
5.3 (4.1, 6.9)
5.1 (3.2, 7.4)
5.9 (4.1, 8.3)
Norovirus
4.9 (3.8, 6.4)
4.0 (2.5, 5.8)
5.2 (3.8, 7.0)
C. jejuni/C. coli
3.6 (2.5, 5.7)
3.0 (1.4, 5.6)
5.2 (3.4, 8.2)
Cryptosporidium
2.2 (1.6, 3.3)
2.6 (1.2, 4.2)
1.9 (1.0, 3.0)
tEPEC
2.0 (1.0, 3.7)
1.2 (0.5, 2.6)
1.3 (0.5, 2.8)
Any antibiotic for diarrhea
Fluoroquinolones for diarrhea
Macrolides for diarrhea
0
5
10
15
20
25
Attributable Fractions
B
Shigella
3.2 (2.9, 3.7)
12.2 (10.4, 14.5)
5.5 (4.7, 6.6)
Rotavirus
2.4 (2.1, 2.7)
7.1 (5.4, 9.0)
4.0 (3.4, 4.8)
Sapovirus
2.2 (1.8, 2.8)
5.9 (4.3, 7.5)
3.3 (2.6, 4.1)
Adenovirus 40/41
2.1 (1.8, 2.6)
6.9 (5.6, 8.8)
5.3 (4.5, 6.4)
ST−ETEC
1.9 (1.5, 2.3)
6.5 (4.9, 8.4)
3.3 (2.5, 4.2)
Astrovirus
1.5 (1.1, 1.9)
3.0 (1.9, 4.3)
2.0 (1.4, 2.8)
Norovirus
1.3 (1.1, 1.8)
2.3 (1.4, 3.4)
1.8 (1.3, 2.4)
C. jejuni/C. coli
1.0 (0.7, 1.6)
1.7 (0.8, 3.3)
1.8 (1.1, 2.8)
Cryptosporidium
0.6 (0.4, 0.9)
1.5 (0.7, 2.4)
0.7 (0.3, 1.0)
tEPEC
0.6 (0.3, 1.0)
0.7 (0.3, 1.5)
0.5 (0.2, 0.9)
All antibiotic courses
All fluoroquinolone courses
All macrolide courses
0
5
10
15
20
25
Attributable Fractions

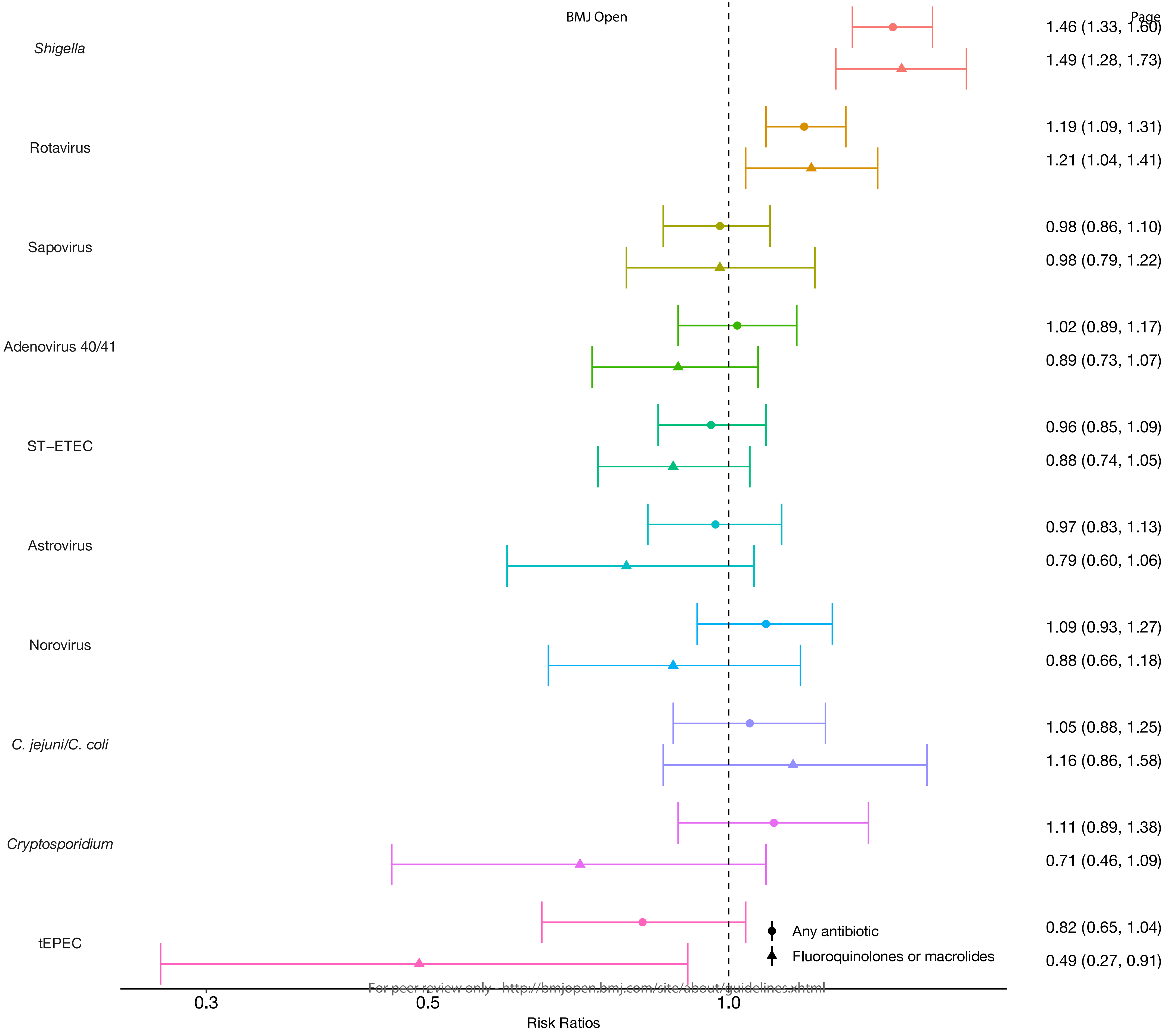

For peer review only - http://bmjopen.bmj.com/site/about/guidelines.xhtml

Supplementary Online Content

Antibiotic use attributable to etiology-specific diarrhea in children under two years of age in low-resource settings

Stephanie A Brennhofer, James A Platts-Mills, Joseph A Lewnard, Jie Liu, Eric R Houpt, Elizabeth T Rogawski McQuade

For peer review only

Table S1. Attributable incidence of pathogen-specific antibiotic courses for diarrhea by antibiotic drug class among 1715 children in the MAL-ED cohort.

	Any antibiotic	Cephalosporins	Fluoroquinolones	Macrolides	Metronidazole	Penicillins	Sulfonamides
Shigella	14.77 (13.25, 16.84)	2.14 (1.66, 2.76)	3.09 (2.64, 3.66)	4.59 (3.92, 5.47)	4.96 (4.09, 5.97)	1.69 (1.29, 2.23)	1.66 (1.27, 2.15)
Rotavirus	10.90 (9.75, 12.42)	1.51 (1.11, 2.01)	1.79 (1.37, 2.28)	3.33 (2.78, 4.02)	3.53 (2.86, 4.37)	1.78 (1.32, 2.40)	1.35 (0.92, 1.85)
Sapovirus	10.24 (8.37, 12.55)	1.47 (0.93, 2.10)	1.48 (1.09, 1.90)	2.75 (2.16, 3.41)	3.95 (2.87, 5.28)	1.57 (1.16, 2.09)	1.56 (1.17, 2.07)
Adenovirus 40/41	9.63 (8.27, 11.69)	1.01 (0.73, 1.43)	1.75 (1.42, 2.22)	4.43 (3.74, 5.35)	1.59 (1.03, 2.47)	1.88 (1.49, 2.48)	0.68 (0.46, 1.00)
ST-ETEC	8.56 (7.04, 10.71)	0.85 (0.56, 1.36)	1.63 (1.24, 2.12)	2.74 (2.11, 3.50)	2.51 (1.83, 3.53)	1.40 (0.99, 2.01)	0.99 (0.64, 1.44)
Astrovirus	6.72 (5.22, 8.77)	1.44 (0.98, 2.11)	0.75 (0.48, 1.09)	1.68 (1.17, 2.36)	2.36 (1.60, 3.38)	1.20 (0.86, 1.71)	0.99 (0.70, 1.41)
Norovirus	6.14 (4.85, 8.03)	0.51 (0.26, 0.97)	0.59 (0.37, 0.86)	1.48 (1.08, 1.99)	1.97 (1.26, 3.12)	1.45 (1.07, 2.04)	1.16 (0.85, 1.63)
C. jejuni/C. coli	4.61 (3.14, 7.19)	0.63 (0.32, 1.10)	0.44 (0.21, 0.83)	1.47 (0.95, 2.33)	1.25 (0.48, 2.37)	0.87 (0.52, 1.33)	0.88 (0.58, 1.31)
Cryptosporidium	2.83 (1.97, 4.15)	0.39 (0.19, 0.73)	0.38 (0.18, 0.62)	0.55 (0.27, 0.86)	0.87 (0.52, 1.58)	0.67 (0.42, 1.13)	0.45 (0.24, 0.71)
tEPEC	2.54 (1.27, 4.69)	0.51 (0.13, 1.11)	0.18 (0.08, 0.39)	0.38 (0.14, 0.79)	1.32 (0.43, 2.80)	0.56 (0.26, 1.08)	0.43 (0.20, 0.83)

Data are attributable incidence rates per 100 child years with 95% confidence intervals (CIs). These data also reported in Figure 1A. Abbreviations: *C. jejuni/C. coli = Campylobacter jejuni/Campylobacter coli*. ST-ETEC= heat-stable enterotoxigenic *Escherichia coli*. tEPEC = typical enteropathogenic *Escherichia coli*.

For peer review only

Table S2. Pathogen-specific attributable fractions of antibiotic courses for diarrhea by antibiotic drug class among 1715 children in the MAL-ED cohort.

	Any Antibiotic	Cephalosporins	Fluoroquinolones	Macrolides	Metronidazole	Penicillins	Sulfonamides
Shigella	11.7 (10.5, 13.3)	8.9 (6.9, 11.5)	20.9 (17.9, 24.7)	16.2 (13.8, 19.3)	10.3 (8.5, 12.4)	7.0 (5.4, 9.3)	9.8 (7.5, 12.7)
Rotavirus	8.6 (7.7, 9.8)	6.3 (4.6, 8.4)	12.1 (9.3, 15.4)	11.8 (9.8, 14.2)	7.3 (5.9, 9.0)	7.4 (5.5, 10.0)	8.0 (5.4, 10.9)
Sapovirus	8.1 (6.6, 9.9)	6.2 (3.9, 8.8)	10.0 (7.4, 12.9)	9.7 (7.6, 12.0)	8.2 (5.9, 10.9)	6.5 (4.8, 8.7)	9.2 (6.9, 12.2)
Adenovirus 40/41	7.6 (6.5, 9.3)	4.2 (3.0, 6.0)	11.8 (9.6, 15.0)	15.6 (13.2, 18.9)	3.3 (2.1, 5.1)	7.8 (6.2, 10.3)	4.0 (2.7, 5.9)
ST-ETEC	6.8 (5.6, 8.5)	3.6 (2.3, 5.7)	11.0 (8.4, 14.4)	9.7 (7.4, 12.4)	5.2 (3.8, 7.3)	5.8 (4.1, 8.4)	5.8 (3.8, 8.5)
Astrovirus	5.3 (4.1, 6.9)	6.0 (4.1, 8.8)	5.1 (3.3, 7.4)	5.9 (4.1, 8.3)	4.9 (3.3, 7.0)	5.0 (3.6, 7.1)	5.9 (4.2, 8.3)
Norovirus	4.9 (3.8, 6.4)	2.1 (1.1, 4.1)	4.0 (2.5, 5.8)	5.2 (3.8, 7.0)	4.1 (2.6, 6.5)	6.1 (4.4, 8.5)	6.8 (5.0, 9.6)
C. jejuni/C. coli	3.6 (2.5, 5.7)	2.6 (1.3, 4.6)	3.0 (1.4, 5.6)	5.2 (3.4, 8.2)	2.6 (1.0, 4.9)	3.6 (2.2, 5.5)	5.2 (3.4, 7.7)
Cryptosporidium	2.2 (1.6, 3.3)	1.6 (0.8, 3.1)	2.6 (1.2, 4.2)	1.9 (1.0, 3.0)	1.8 (1.1, 3.3)	2.8 (1.7, 4.7)	2.6 (1.4, 4.2)
tEPEC	2.0 (1.0, 3.7)	2.1 (0.5, 4.6)	1.2 (0.5, 2.6)	1.3 (0.5, 2.8)	2.7 (0.9, 5.8)	2.3 (1.1, 4.5)	2.6 (1.2, 4.9)

Data are attributable fraction percentages with 95% confidence intervals (CIs). These data also reported in Figure 2A. Abbreviations: *C. jejuni/C. coli* = *Campylobacter jejuni/Campylobacter coli*. ST-ETEC= heat-stable enterotoxigenic *Escherichia coli*. tEPEC = typical enteropathogenic *Escherichia coli*.

For peer review only

Table S3. Pathogen-specific attributable fractions of antibiotic courses for diarrhea by site among 1715 children in the MAL-ED cohort.

	Dhaka, Bangladesh	Fortaleza, Brazil	Vellore, India	Bhaktapur, Nepal	Loreto, Peru	Naushero Feroze, Pakistan	Venda, South Africa	Tanzania
Shigella	21.4 (18.6, 25.6)	10.6 (0.0, 29.6)	17.0 (11.8, 23.3)	15.0 (11.1, 19.9)	11.6 (8.7, 14.9)	6.4 (4.8, 8.5)	6.9 (0.0, 15.0)	7.9 (1.0, 16.0)
Rotavirus	16.0 (13.7, 19.1)	0.0 (0.0, 0.0)	12.2 (7.6, 17.4)	7.9 (5.4, 11.0)	5.5 (3.9, 7.5)	5.5 (4.4, 7.1)	3.3 (0.0, 11.6)	12.4 (6.1, 19.9)
Sapovirus	8.5 (5.5, 11.4)	15.1 (0.0, 37.7)	9.9 (5.8, 14.7)	9.6 (6.2, 13.5)	11.6 (9.0, 14.7)	5.8 (3.3, 8.7)	3.6 (0.2, 7.8)	10.9 (4.4, 18.2)
Adenovirus 40/41	23.9 (20.0, 29.1)	6.8 (0.0, 24.3)	7.9 (3.8, 13.0)	2.1 (0.1, 4.6)	6.5 (4.6, 9.0)	1.3 (0.1, 3.3)	1.6 (0.0, 4.6)	7.1 (1.7, 14.5)
ST-ETEC	14.7 (11.2, 18.9)	3.7 (0.0, 15.8)	7.3 (3.7, 11.7)	5.9 (3.5, 9.2)	3.2 (1.5, 5.6)	3.1 (1.7, 5.5)	5.4 (0.0, 15.6)	15.6 (6.6, 25.2)
Astrovirus	5.2 (2.4, 8.2)	0.0 (0.0, 0.0)	6.7 (3.4, 11.3)	3.1 (1.5, 5.0)	8.8 (6.5, 12.2)	4.8 (2.8, 7.2)	6.2 (0.6, 16.1)	2.0 (0.0, 5.8)
Norovirus	5.0 (2.9, 7.1)	6.5 (0.0, 21.8)	2.9 (0.4, 5.9)	5.3 (3.2, 8.4)	8.9 (6.5, 11.8)	2.3 (0.9, 5.1)	4.5 (0.0, 13.2)	12.3 (6.3, 19.9)
C. jejuni/C. coli	2.7 (0.3, 6.8)	0.0 (0.0, 0.0)	4.6 (1.6, 8.2)	3.1 (0.6, 6.9)	9.6 (5.9, 13.4)	1.9 (0.1, 4.5)	2.2 (0.0, 7.3)	4.1 (0.0, 13.3)
Cryptosporidium	1.9 (0.6, 3.3)	3.7 (0.0, 13.5)	4.7 (1.9, 9.8)	2.5 (0.6, 4.7)	3.7 (1.9, 5.6)	1.5 (0.6, 2.9)	0.1 (0.0, 1.2)	2.9 (0.0, 8.7)
tEPEC	1.1 (0.1, 3.0)	0.0 (0.0, 1.5)	1.8 (0.2, 4.8)	1.2 (0.2, 3.3)	1.6 (0.2, 3.5)	2.5 (0.4, 5.8)	0.0 (0.0, 2.1)	4.4 (0.0, 12.9)

Data are attributable fraction percentages with 95% confidence intervals (CIs). Abbreviations: *C. jejuni/C. coli* = *Campylobacter jejuni/Campylobacter coli*. ST-ETEC= heat-stable enterotoxigenic *Escherichia coli*. tEPEC = typical enteropathogenic *Escherichia coli*.

For peer review only

For peer review only - http://bmjopen.bmj.com/site/about/guidelines.xhtml

Table S4. Pathogen-specific attributable fractions of all antibiotic courses by site among 1715 children in the MAL-ED cohort.

	Dhaka, Bangladesh	Fortaleza, Brazil	Vellore, India	Bhaktapur, Nepal	Loreto, Peru	Naushero Feroze, Pakistan	Venda, South Africa	Haydom, Tanzania
Shigella	5.2 (4.5, 6.2)	0.9 (0.0, 2.4)	2.4 (1.6, 3.2)	4.5 (3.3, 6.0)	3.9 (2.9, 5.0)	2.4 (1.8, 3.2)	0.8 (0.0, 1.8)	1.4 (0.2, 2.9)
Rotavirus	3.9 (3.3, 4.6)	0.0 (0.0, 0.0)	1.7 (1.1, 2.4)	2.4 (1.6, 3.3)	1.9 (1.3, 2.5)	2.1 (1.6, 2.6)	0.4 (0.0, 1.4)	2.3 (1.1, 3.7)
Sapovirus	2.1 (1.3, 2.8)	1.2 (0.0, 3.0)	1.4 (0.8, 2.0)	2.9 (1.9, 4.1)	3.9 (3.0, 5.0)	2.2 (1.2, 3.2)	0.4 (0.0, 1.0)	2.0 (0.8, 3.3)
Adenovirus 40/41	5.8 (4.9, 7.1)	0.5 (0.0, 2.0)	1.1 (0.5, 1.8)	0.6 (0.0, 1.4)	2.2 (1.5, 3.1)	0.5 (0.0, 1.2)	0.2 (0.0, 0.6)	1.3 (0.3, 2.7)
ST-ETEC	3.6 (2.7, 4.6)	0.3 (0.0, 1.3)	1.0 (0.5, 1.6)	1.8 (1.0, 2.8)	1.1 (0.5, 1.9)	1.2 (0.6, 2.0)	0.7 (0.0, 1.9)	2.9 (1.2, 4.6)
Astrovirus	1.3 (0.6, 2.0)	0.0 (0.0, 0.0)	0.9 (0.5, 1.6)	0.9 (0.5, 1.5)	3.0 (2.2, 4.1)	1.8 (1.1, 2.7)	0.8 (0.1, 2.0)	0.4 (0.0, 1.1)
Norovirus	1.2 (0.7, 1.7)	0.5 (0.0, 1.8)	0.4 (0.1, 0.8)	1.6 (1.0, 2.5)	3.0 (2.2, 4.0)	0.9 (0.3, 1.9)	0.5 (0.0, 1.6)	2.3 (1.2, 3.7)
C. jejuni/C. coli	0.6 (0.1, 1.7)	0.0 (0.0, 0.0)	0.6 (0.2, 1.1)	0.9 (0.2, 2.1)	3.2 (2.0, 4.5)	0.7 (0.0, 1.7)	0.3 (0.0, 0.9)	0.8 (0.0, 2.4)
Cryptosporidium	0.5 (0.1, 0.8)	0.3 (0.0, 1.1)	0.6 (0.3, 1.4)	0.8 (0.2, 1.4)	1.2 (0.6, 1.9)	0.5 (0.2, 1.1)	0.0 (0.0, 0.2)	0.5 (0.0, 1.6)
tEPEC	0.3 (0.0, 0.7)	0.0 (0.0, 0.1)	0.3 (0.0, 0.7)	0.4 (0.1, 1.0)	0.5 (0.1, 1.2)	0.9 (0.1, 2.2)	0.0 (0.0, 0.3)	0.8 (0.0, 2.4)

Data are attributable fraction percentages with 95% confidence intervals (CIs). Abbreviations: *C. jejuni/C. coli* = *Campylobacter jejuni/Campylobacter coli*. ST-ETEC= heat-stable enterotoxigenic *Escherichia coli*. tEPEC = typical enteropathogenic *Escherichia coli*.

For peer review only

For peer review only - http://bmjopen.bmj.com/site/about/guidelines.xhtml

Table S5. Attributable incidence of pathogen-specific antibiotic courses for diarrhea by site among 1715 children in the MAL-ED cohort.

	Dhaka, Bangladesh	Fortaleza, Brazil	Vellore, India	Bhaktapur, Nepal	Loreto, Peru	Naushero Feroze, Pakistan	Venda, South Africa	Haydom, Tanzania
Shigella	45.79 (39.70, 54.61)	0.58 (0.00, 1.62)	9.08 (6.27, 12.4)	10.57 (7.79, 14.01)	20.54 (15.49, 26.48)	23.93 (17.88, 31.62)	0.90 (0.00, 1.96)	5.12 (0.65, 10.42)
Rotavirus	34.20 (29.21, 40.74)	0.00 (0.00, 0.00)	6.48 (4.02, 9.29)	5.53 (3.79, 7.73)	9.83 (6.85, 13.26)	20.68 (16.33, 26.47)	0.43 (0.00, 1.51)	8.08 (3.94, 12.95)
Sapovirus	18.10 (11.79, 24.44)	0.82 (0.00, 2.06)	5.29 (3.09, 7.82)	6.71 (4.38, 9.49)	20.50 (16.03, 26.07)	21.64 (12.24, 32.38)	0.47 (0.03, 1.02)	7.10 (2.88, 11.86)
Adenovirus 40/41	50.99 (42.72, 62.14)	0.37 (0.00, 1.33)	4.23 (2.04, 6.91)	1.47 (0.10, 3.24)	11.52 (8.13, 16.04)	4.87 (0.32, 12.21)	0.20 (0.00, 0.60)	4.64 (1.14, 9.44)
ST-ETEC	31.42 (23.92, 40.30)	0.20 (0.00, 0.86)	3.90 (1.98, 6.21)	4.18 (2.45, 6.46)	5.67 (2.65, 9.92)	11.52 (6.48, 20.50)	0.70 (0.00, 2.04)	10.14 (4.27, 16.42)
Astrovirus	11.18 (5.02, 17.56)	0.00 (0.00, 0.00)	3.59 (1.81, 6.02)	2.20 (1.08, 3.55)	15.67 (11.49, 21.70)	17.74 (10.62, 27.00)	0.82 (0.07, 2.11)	1.29 (0.00, 3.77)
Norovirus	10.64 (6.13, 15.20)	0.36 (0.00, 1.19)	1.56 (0.22, 3.12)	3.74 (2.23, 5.89)	15.80 (11.53, 20.94)	8.76 (3.24, 19.05)	0.59 (0.00, 1.73)	8.01 (4.08, 12.98)
C. jejuni/C. coli	5.70 (0.68, 14.56)	0.00 (0.00, 0.00)	2.46 (0.83, 4.38)	2.20 (0.45, 4.82)	17.06 (10.47, 23.71)	6.96 (0.41, 16.90)	0.29 (0.00, 0.96)	2.67 (0.00, 8.62)
Cryptosporidium	4.08 (1.19, 7.00)	0.20 (0.00, 0.74)	2.48 (1.02, 5.24)	1.77 (0.41, 3.29)	6.47 (3.39, 9.97)	5.50 (2.43, 10.81)	0.01 (0.00, 0.16)	1.87 (0.00, 5.64)
tEPEC	2.43 (0.29, 6.41)	0.00 (0.00, 0.08)	0.99 (0.08, 2.56)	0.88 (0.14, 2.30)	2.82 (0.37, 6.23)	9.26 (1.49, 21.69)	0.00 (0.00, 0.28)	2.86 (0.00, 8.39)

Data are attributable incidence rates per 100 child years with 95% confidence intervals (CIs). These data also reported in Figure 1B. Abbreviations: *C. jejuni/C. coli* = *Campylobacter jejuni/Campylobacter coli*. ST-ETEC= heat-stable enterotoxigenic *Escherichia coli*. tEPEC = typical enteropathogenic *Escherichia coli*.

For peer review only

For peer review only - http://bmjopen.bmj.com/site/about/guidelines.xhtml

Figure S1. Attributable incidence of pathogen-specific antibiotic courses for diarrhea in the first and second year of life among 1715 children in the MAL-ED cohort. Error bars show 95% CI. *C. jejuni/C. coli* = *Campylobacter jejuni/Campylobacter coli*. ST-ETEC= heat-stable enterotoxigenic *Escherichia coli*. tEPEC = typical enteropathogenic *Escherichia coli*.

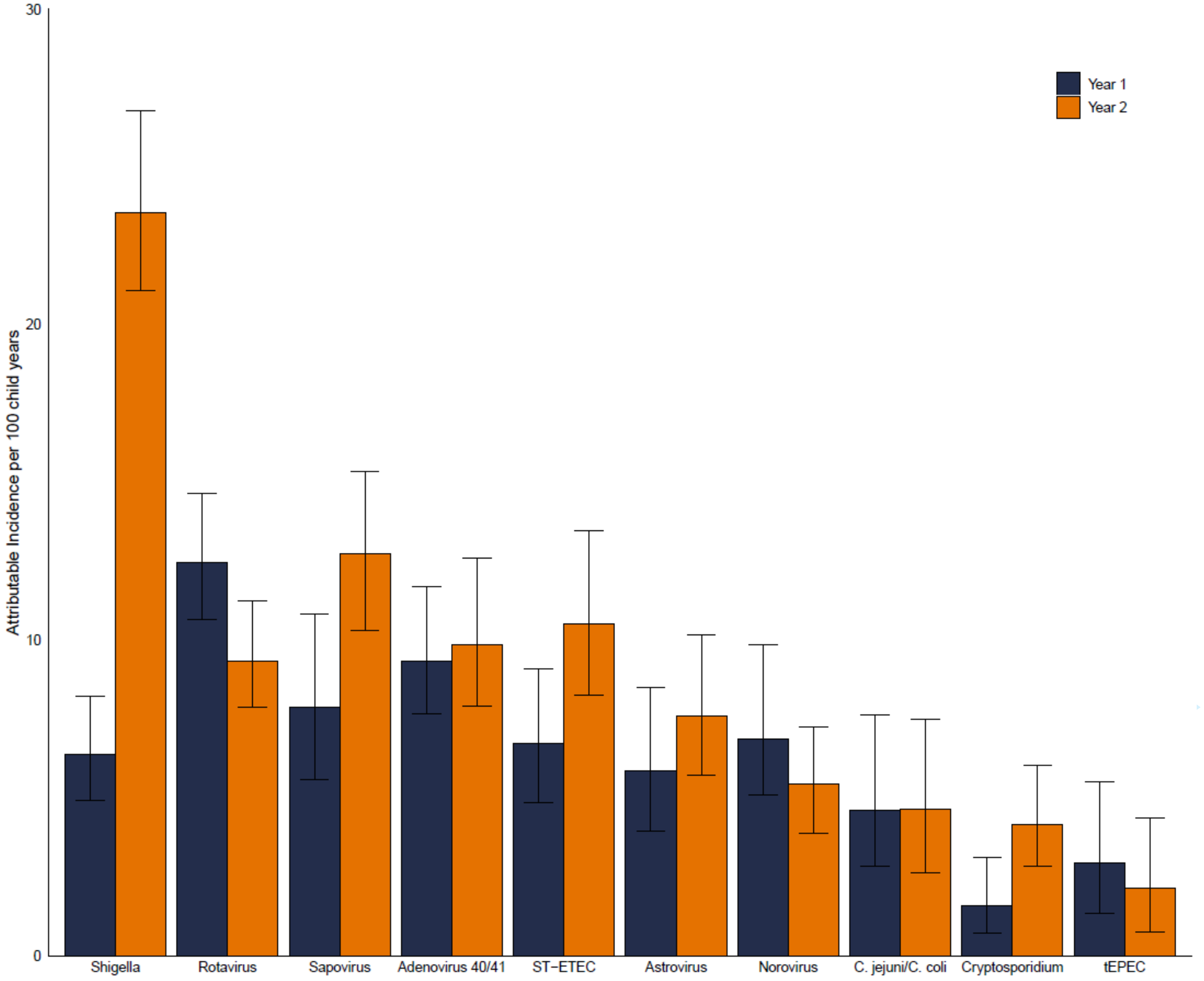

Table S6. Attributable incidence of pathogen-specific antibiotic courses for diarrhea in the first and second year of life among 1715 children in the MAL-ED cohort.

	Year 1 (0-11 months)	Year 2 (12-23 months)
Shigella	6.39 (4.91, 8.21)	23.55 (21.08, 26.79)
Rotavirus	12.48 (10.67, 14.65)	9.33 (7.87, 11.24)
Sapovirus	7.89 (5.58, 10.84)	12.75 (10.32, 15.36)
Adenovirus 40/41	9.35 (7.67, 11.71)	9.87 (7.92, 12.61)
ST-ETEC	6.74 (4.87, 9.10)	10.52 (8.26, 13.49)
Astrovirus	5.87 (3.94, 8.49)	7.61 (5.72, 10.17)
Norovirus	6.86 (5.09, 9.86)	5.43 (3.88, 7.25)
C. jejuni/C. coli	4.62 (2.85, 7.62)	4.65 (2.63, 7.49)
Cryptosporidium	1.60 (0.72, 3.10)	4.15 (2.82, 6.03)
tEPEC	2.95 (1.35, 5.52)	2.13 (0.77, 4.38)

Data are attributable incidence rates per 100 child years with 95% confidence intervals (CIs). These data also reported in Figure S1. Abbreviations: *C. jejuni/C. coli* = *Campylobacter jejuni/Campylobacter coli*. ST-ETEC= heat-stable enterotoxigenic *Escherichia coli*. tEPEC = typical enteropathogenic *Escherichia coli*.

For peer review only

Table S7. Pathogen-specific attributable fractions of all antibiotic courses by antibiotic drug class among 1715 children in the MAL-ED cohort.

	Any Antibiotic	Cephalosporins	Fluoroquinolones	Macrolides	Metronidazole	Penicillins	Sulfonamides
Shigella	3.2 (2.9, 3.7)	2.0 (1.6, 2.6)	12.2 (10.5, 14.5)	5.5 (4.7, 6.6)	6.7 (5.5, 8.0)	0.9 (0.7, 1.2)	3.6 (2.8, 4.7)
Rotavirus	2.4 (2.1, 2.7)	1.4 (1.1, 1.9)	7.1 (5.4, 9.0)	4.0 (3.3, 4.8)	4.7 (3.8, 5.9)	1.0 (0.7, 1.3)	3.0 (2.0, 4.1)
Sapovirus	2.2 (1.8, 2.7)	1.4 (0.9, 2.0)	5.9 (4.3, 7.5)	3.3 (2.6, 4.1)	5.3 (3.9, 7.1)	0.9 (0.6, 1.1)	3.4 (2.6, 4.5)
Adenovirus 40/41	2.1 (1.8, 2.6)	1.0 (0.7, 1.4)	6.9 (5.6, 8.8)	5.3 (4.5, 6.4)	2.1 (1.4, 3.3)	1.0 (0.8, 1.4)	1.5 (1.0, 2.2)
ST-ETEC	1.9 (1.5, 2.3)	0.8 (0.5, 1.3)	6.5 (4.9, 8.4)	3.3 (2.5, 4.2)	3.4 (2.5, 4.7)	0.8 (0.5, 1.1)	2.2 (1.4, 3.2)
Astrovirus	1.5 (1.1, 1.9)	1.4 (0.9, 2.0)	3.0 (1.9, 4.3)	2.0 (1.4, 2.8)	3.2 (2.1, 4.5)	0.7 (0.5, 0.9)	2.2 (1.5, 3.1)
Norovirus	1.3 (1.1, 1.8)	0.5 (0.2, 0.9)	2.3 (1.4, 3.4)	1.8 (1.3, 2.4)	2.6 (1.7, 4.2)	0.8 (0.6, 1.1)	2.5 (1.9, 3.6)
C. jejuni/C. coli	1.0 (0.7, 1.6)	0.6 (0.3, 1.0)	1.7 (0.8, 3.3)	1.8 (1.1, 2.8)	1.7 (0.6, 3.2)	0.5 (0.3, 0.7)	1.9 (1.3, 2.9)
Cryptosporidium	0.6 (0.4, 0.9)	0.4 (0.2, 0.7)	1.5 (0.7, 2.4)	0.7 (0.3, 1.0)	1.2 (0.7, 2.1)	0.4 (0.2, 0.6)	1.0 (0.5, 1.6)
tEPEC	0.6 (0.3, 1.0)	0.5 (0.1, 1.1)	0.7 (0.3, 1.5)	0.5 (0.2, 1.0)	1.8 (0.6, 3.8)	0.3 (0.1, 0.6)	0.9 (0.4, 1.8)

Data are attributable fraction percentages with 95% confidence intervals (CIs). These data also reported in Figure 2B. Abbreviations: *C. jejuni/C. coli* = *Campylobacter jejuni/Campylobacter coli*. ST-ETEC= heat-stable enterotoxigenic *Escherichia coli*. tEPEC = typical enteropathogenic *Escherichia coli*.

For peer review only

Table S8. Pathogen-specific attributable fractions of antibiotic courses for dysentery and watery diarrhea among 1,715 children in the MAL-ED cohort

	**Attributable fraction of antibiotic courses for dysentery**	**Attributable fraction of antibiotic courses for watery diarrhea**	**Proportion of all attributable antibiotic courses that were for dysentery**
Shigella	27.5% (23.4, 33.1)	10.3% (9.2, 11.8)	18.7% (16.2, 21.4)
Rotavirus	3.8% (2.3, 5.8)	9.1% (8.1, 10.4)	3.5% (2.1, 5.1)
Sapovirus	5.9% (3.8, 8.6)	8.3% (6.8, 10.2)	5.8% (3.9, 7.9)
Adenovirus 40/41	7.4% (5.6, 10.2)	7.6% (6.5, 9.3)	7.8% (5.9, 9.9)
ST-ETEC	5.7% (3.2, 8.4)	6.9% (5.7, 8.7)	6.7% (4.1, 9.1)
Astrovirus	2.6% (1.5, 4.3)	5.6% (4.3, 7.3)	3.9% (2.5, 5.8)
Norovirus	4.0% (2.3, 6.3)	4.9% (3.8, 6.5)	6.6% (4.0, 9.6)
C. jejuni/C. coli	8.5% (5.8, 12.2)	3.2% (2.1, 5.4)	18.6% (12.7, 25.5)
Cryptosporidium	1.3% (0.5, 3.0)	2.3% (1.6, 3.4)	4.7% (1.7, 9.0)
tEPEC	1.5% (0.5, 3.5)	2.1% (1.0, 3.8)	6.0% (2.2, 11.8)

Data are attributable fraction percentages with 95% confidence intervals (CIs). [a] Positive value indicates the pathogen was responsible for a larger proportion of antibiotic-treated dysentery diarrheal episodes compared to antibiotic-treated watery diarrhea episodes. Abbreviations: *C. jejuni/C. coli* = *Campylobacter jejuni/Campylobacter coli*. ST-ETEC= heat-stable enterotoxigenic *Escherichia coli*. tEPEC = typical enteropathogenic *Escherichia coli*.

For peer review only - http://bmjopen.bmj.com/site/about/guidelines.xhtml

Table S9 Risk ratios for antibiotic treatment comparing episode-specific attributable fractions for each pathogen by site among 1715 children in the MAL-ED cohort.

	Dhaka, Bangladesh	Vellore, India	Bhaktapur, Nepal	Loreto, Peru	Naushero Feroze, Pakistan	Haydom, Tanzania
Shigella	1.40 (1.22, 1.61)	1.55 (1.03, 2.33)	2.30 (1.70, 3.11)	1.35 (1.08, 1.68)	1.38 (1.17, 1.62)	0.77 (0.34, 1.72)
Rotavirus	1.10 (0.95, 1.27)	1.67 (1.13, 2.46)	0.70 (0.46, 1.05)	1.37 (1.06, 1.79)	1.48 (1.31, 1.68)	1.04 (0.64, 1.69)
Sapovirus	1.04 (0.83, 1.29)	0.93 (0.53, 1.62)	0.76 (0.49, 1.18)	0.86 (0.68, 1.09)	1.02 (0.84, 1.25)	1.23 (0.77, 1.98)
Adenovirus 40/41	1.01 (0.87, 1.18)	1.85 (0.84, 4.07)	1.07 (0.35, 3.21)	0.73 (0.49, 1.10)	..	1.65 (0.84, 3.26)
ST-ETEC	0.87 (0.74, 1.03)	0.84 (0.48, 1.46)	0.74 (0.48, 1.13)	0.91 (0.61, 1.36)	1.32 (1.01, 1.71)	0.87 (0.56, 1.37)
Astrovirus	1.00 (0.74, 1.34)	1.08 (0.57, 2.02)	0.54 (0.26, 1.11)	0.94 (0.70, 1.26)	1.08 (0.88, 1.34)	0.49 (0.10, 2.37)
Norovirus	1.40 (1.08, 1.80)	0.66 (0.20, 2.14)	0.58 (0.33, 1.04)	1.12 (0.83, 1.50)	1.36 (1.01, 1.83)	1.13 (0.67, 1.88)
C. jejuni/C. coli	0.66 (0.43, 1.02)	0.75 (0.33, 1.69)	..	1.43 (1.15, 1.78)	0.87 (0.58, 1.32)	..
Cryptosporidium	1.03 (0.71, 1.48)	2.11 (1.18, 3.79)	1.31 (0.64, 2.66)	0.72 (0.46, 1.15)	1.19 (0.83, 1.71)	3.18 (1.36, 7.43)
tEPEC	0.44 (0.22, 0.89)	..	0.41 (0.12, 1.38)	0.99 (0.54, 1.81)	0.97 (0.74, 1.28)	1.87 (0.98, 3.57)

Data are risk ratios (RR) with 95% confidence intervals (CIs). The model is adjusted for: all pathogens, age, sex, and the water, assets, maternal education, income (WAMI) index. Site data from Brazil and South Africa were removed as there were not enough diarrheal episodes to model the data. Select pathogen data by site is missing in cases where there were no treated diarrheal episodes. Abbreviations: *C. jejuni/C. coli* = *Campylobacter jejuni/Campylobacter coli*. ST-ETEC= heat-stable enterotoxigenic *Escherichia coli*. tEPEC = typical enteropathogenic *Escherichia coli*.

Table S10. Mediation analysis assessing whether diarrhea severity and dysentery mediated the relationship between Shigella diarrhea and antibiotic treatment among 1715 children in the MAL-ED cohort.

	Shigella (Mediated by diarrhea severity)		*Shigella* (Mediated by dysentery)	
	Any antibiotic	Fluoroquinolones or macrolides	Any antibiotic	Fluoroquinolones or macrolides
Total Effect Rate Ratio	1.30 (1.21, 1.40)	1.39 (1.21, 1.58)	1.30 (1.21, 1.40)	1.39 (1.21, 1.58)
Pure Natural Direct Effect Rate Ratio	1.29 (1.18, 1.40)	1.33 (1.14, 1.55)	1.25 (1.14, 1.37)	1.23 (1.05, 1.44)
Total Natural Indirect Effect Rate Ratio	1.01 (0.96, 1.06)	1.04 (0.95, 1.14)	1.04 (0.99, 1.10)	1.13 (1.02, 1.25)
Proportion Mediated	0.05 (0.00, 0.27)	0.14 (0.00, 0.47)	0.18 (0.00, 0.43)	0.40 (0.08, 0.82)

Data are risk ratios (RR) with 95% confidence intervals (CIs).

For peer review only

For peer review only - http://bmjopen.bmj.com/site/about/guidelines.xhtml

BMJ Open

Antibiotic use attributable to specific etiologies of diarrhea in children under two years of age in low-resource settings: a secondary analysis of the MAL-ED birth cohort

Journal:	*BMJ Open*
Manuscript ID	bmjopen-2021-058740.R1
Article Type:	Original research
Date Submitted by the Author:	07-Mar-2022
Complete List of Authors:	Brennhofer, Stephanie; University of Virginia School of Medicine, Division of Infectious Diseases and International Health<br>Platts-Mills, James; University of Virginia School of Medicine, Division of Infectious Diseases and International Health<br>Lewnard, Joseph; University of California Berkeley, Division of Epidemiology<br>Liu, Jie; University of Virginia School of Medicine, Division of Infectious Diseases and International Health; Qingdao University, School of Public Health<br>Houpt, Eric; University of Virginia School of Medicine, Division of Infectious Diseases and International Health<br>Rogawski McQuade, Elizabeth; University of Virginia School of Medicine, Division of Infectious Diseases and International Health; Emory University, Department of Epidemiology
**Primary Subject Heading**:	Infectious diseases
Secondary Subject Heading:	Epidemiology, Global health, Paediatrics
Keywords:	Gastrointestinal infections < GASTROENTEROLOGY, Community child health < PAEDIATRICS, INFECTIOUS DISEASES, Epidemiology < INFECTIOUS DISEASES, Paediatric infectious disease & immunisation < PAEDIATRICS, BACTERIOLOGY

SCHOLARONE™
Manuscripts

I, the Submitting Author has the right to grant and does grant on behalf of all authors of the Work (as defined in the below author licence), an exclusive licence and/or a non-exclusive licence for contributions from authors who are: i) UK Crown employees; ii) where BMJ has agreed a CC-BY licence shall apply, and/or iii) in accordance with the terms applicable for US Federal Government officers or employees acting as part of their official duties; on a worldwide, perpetual, irrevocable, royalty-free basis to BMJ Publishing Group Ltd ("BMJ") its licensees and where the relevant Journal is co-owned by BMJ to the co-owners of the Journal, to publish the Work in this journal and any other BMJ products and to exploit all rights, as set out in our licence.

The Submitting Author accepts and understands that any supply made under these terms is made by BMJ to the Submitting Author unless you are acting as an employee on behalf of your employer or a postgraduate student of an affiliated institution which is paying any applicable article publishing charge ("APC") for Open Access articles. Where the Submitting Author wishes to make the Work available on an Open Access basis (and intends to pay the relevant APC), the terms of reuse of such Open Access shall be governed by a Creative Commons licence – details of these licences and which Creative Commons licence will apply to this Work are set out in our licence referred to above.

Other than as permitted in any relevant BMJ Author's Self Archiving Policies, I confirm this Work has not been accepted for publication elsewhere, is not being considered for publication elsewhere and does not duplicate material already published. I confirm all authors consent to publication of this Work and authorise the granting of this licence.

For peer review only

Antibiotic use attributable to specific etiologies of diarrhea in children under two years of age in low-resource settings: a secondary analysis of the MAL-ED birth cohort

Stephanie A Brennhofer, MPH[1]; James A Platts-Mills, MD[1]; Joseph A Lewnard, PhD[2]; Jie Liu, PhD[1,3]; Eric R Houpt, MD[1]; Elizabeth T Rogawski McQuade, PhD[1,4]

[1] Division of Infectious Diseases & International Health, School of Medicine, University of Virginia, 345 Crispell Drive, Charlottesville, VA, 22908, USA;

[2] Division of Epidemiology, School of Public Health, University of California - Berkeley, 2121 Berkeley Way, Berkeley, California, 94720, USA;

[3] School of Public Health, Qingdao University, 1 Ningde Road, Qingdao, Shandong Province, 266073, China;

[4] Department of Epidemiology, Rollins School of Public Health, Emory University, 1518 Clifton Road NE, Atlanta, GA, 30322, USA

Correspondence to: Dr. Elizabeth T Rogawski McQuade; erogaws@emory.edu

For peer review only

For peer review only - http://bmjopen.bmj.com/site/about/guidelines.xhtml

ABSTRACT

Objective: To quantify the frequency of antibiotic treatments attributable to specific enteric pathogens due to the treatment of diarrhea among children in the first two years of life in low-resource settings.

Design: Secondary analysis of a longitudinal birth cohort study, MAL-ED.

Setting: This study was conducted at eight sites in Bangladesh, Brazil, India, Nepal, Peru, Pakistan, South Africa, and Tanzania.

Participants: We analyzed 9,392 reported diarrhea episodes, including 6,677 with molecular diagnostic test results, as well as 31,408 non-diarrheal stools from 1,715 children aged 0-2 years with two years of complete follow-up data.

Primary and secondary outcome measures: We estimated incidence rates and the proportions of antibiotic use for diarrhea and for all indications attributable to the top ten etiologies of diarrhea. We estimated associations between specific etiologies and antibiotic treatment, and assessed whether clinical characteristics of the diarrhea episodes mediated these relationships.

Results: *Shigella* and rotavirus were the leading causes of antibiotic treatment, responsible for 11.7% and 8.6% of diarrhea treatments and 14.8 and 10.9 courses per 100 child-years, respectively. *Shigella* and rotavirus-attributable diarrhea episodes were 46% (RR:1.46; 95% CI:1.33-1.60), and 19% (RR:1.19; 95% CI:1.09-1.31) more likely to be treated with antibiotics,

For peer review only - http://bmjopen.bmj.com/site/about/guidelines.xhtml

respectively, compared to other etiologies. Considering antibiotic uses for all indications, these two pathogens accounted for 5.5% of all antibiotic courses, 19.4% of all fluoroquinolone courses, and 9.6% of all macrolide courses. Among indicated treatments for dysentery, *Shigella* and *C. jejuni/C. coli* were responsible for 27.5% and 8.5% of treated episodes, respectively.

Conclusions: The evidence that *Shigella* and rotavirus were disproportionately responsible for antibiotic use due to their high burden and severity further strengthens the value of interventions targeted to these pathogens. Interventions against *Campylobacter* could further prevent a large burden of indicated antibiotic treatment for dysentery, which could not be averted by antibiotic stewardship interventions.

For peer review only

SUMMARY BOX

Strengths and limitations of this study?

- The multi-site birth cohort design of this study with intensive twice-weekly home visits allowed capture of all antibiotic exposures for any indication including instances where antibiotics were obtained without prescriptions.
- The use of quantitative molecular diagnostics for a broad range of enteric pathogens allowed us to appropriately assign etiology to diarrhea episodes prompting antibiotic treatment.
- A limitation was that the indication for antibiotic use was not known and was therefore inferred by the overlap between treatment and diarrhea symptoms.

For peer review only

For peer review only - http://bmjopen.bmj.com/site/about/guidelines.xhtml

INTRODUCTION

Diarrhea is a major cause of antibiotic treatment among children, especially in low and middle income countries (LMICs), because of both the high incidence of diarrhea and frequency of treatment. In the multi-site Etiology, Risk Factors, and Interactions of Enteric Infections and Malnutrition and the Consequences for Child Health and Development (MAL-ED) birth cohort study, the incidence of diarrhea during the first two years of life was 273.8 episodes per 100 child years,[1] and 46% of episodes were treated with antibiotics.[2] Less than 5% of episodes were dysenteric and therefore met antibiotic treatment guidelines from the World Health Organization (WHO).[3] Nearly half of non-bloody diarrheal episodes were treated, representing a large burden of inappropriate antibiotic use.[2] Similarly, in the Global Enterics Multicenter Study (GEMS), a seven-site case-control study of moderate-to-severe diarrhea, nearly 75% of non-bloody moderate-to-severe diarrhea episodes were treated with antibiotics among children under five.[4] Frequent antibiotic treatment of diarrhea directly contributes to the development of antimicrobial resistance (AMR) for bacterial diarrheal pathogens, particularly *Shigella* and *Campylobacter*, which are on the WHO priority pathogen list for concern about AMR.[5] Treatment of diarrhea also affects AMR more broadly through antibiotic selection pressure to bacteria carried at the time of treatment.

Because there is uncontrolled access to antibiotics in many LMICs, children often receive antibiotics without seeking care.[6] Even if a child presents to care, clinical predictors and point-of-care diagnostics to identify diarrhea episodes that could respond to antibiotics are largely unavailable.[7] Prescribing antibiotics for diarrhea remains the standard of care in many settings despite the recognized need for antibiotic stewardship and guidelines to reserve antibiotic

For peer review only - http://bmjopen.bmj.com/site/about/guidelines.xhtml

treatment for dysentery.[8] Vaccines or other interventions that prevent diarrheal illnesses from occurring and therefore prompting treatment might provide the most effective mechanism for reducing antibiotic use.[9,10]

Influenza and pneumococcal conjugate vaccines have been found to reduce antibiotic use through the prevention of respiratory illnesses.[11] A recent randomized controlled trial demonstrated that maternal respiratory syncytial virus (RSV) vaccination prevented 13% of antibiotic use in the first three months of life.[12] Additionally, rotavirus vaccination was estimated to prevent 13.6 million antibiotic-treated diarrhea episodes annually among children under two years in LMICs.[13] Estimation of the further reductions in antibiotic use that could be achieved by vaccines against enteric pathogens such as *Shigella*, enterotoxigenic *Escherichia coli* (ETEC), *Campylobacter*, and *Cryptosporidium* appropriately broadens the vaccine value proposition and could inform priority-setting for the development, evaluation, and implementation of these interventions.[14]

To estimate the preventable burden of antibiotic use for diarrhea that could be achieved by vaccines or other pathogen-specific interventions, we quantified the amount of antibiotic use that could be attributed to the treatment of specific causes of diarrhea in the MAL-ED birth cohort study.

METHODS

Study design and participants

The study design for MAL-ED has been described elsewhere.[15] Briefly, this study was conducted

For peer review only - http://bmjopen.bmj.com/site/about/guidelines.xhtml

from November 2009 to February 2014, and participants were enrolled at eight sites: Dhaka, Bangladesh; Fortaleza, Brazil; Vellore, India; Bhaktapur, Nepal; Loreto, Peru; Naushero Feroze, Pakistan; Venda, South Africa; and Haydom, Tanzania. Children were followed from birth (<17 days of age) through age 24 months. Fieldworkers conducted twice weekly home visits in which they collected information on antibiotic drug classes given to the child and diarrhea since the last home visit. Diarrhea was defined as three or more loose stools in a 24-hour period or visible blood in at least one stool. Diarrheal episodes were separated by at least two days without diarrhea. Stool samples were collected during diarrhea and monthly in the absence of diarrhea. Episode severity was defined by a modified Vesikari score, previously described.[16] Dysentery was defined as reported presence of blood in at least one stool during a diarrheal episode. Antibiotic courses for diarrhea were identified when antibiotic use was reported on any day during a diarrhea episode. Distinct antibiotic courses not associated with diarrhea were defined if separated by at least two days of no antibiotic use, as previously.[2]

Stool testing

Pathogens were detected among all stool samples collected from children with complete follow-up. To extract total nucleic acid, the QIAmp Fast DNA Stool Mini Kit (Qiagen) was used.[17] Quantitative polymerase chain reaction (qPCR) using AgPath One Step realtime PCR kit (Thermo-Fisher) was used to detect 29 enteropathogens via the TaqMan Array Card (TAC) platform.[1] A quantification cycle (Cq) threshold of 35 was the analytic limit of detection. Ten enteric pathogens that were previously identified as the top causes of diarrhea in MAL-ED[1] were included in these analyses: adenovirus 40/41, astrovirus, *Campylobacter jenjui/Campylobacter coli* (*C. jejuni/C.coli*), *Cryptosporidium*, norovirus, rotavirus, sapovirus, *Shigella*, typical

For peer review only - http://bmjopen.bmj.com/site/about/guidelines.xhtml

enteropathogenic *Escherichia coli* (tEPEC), and heat stable enterotoxigenic *Escherichia coli* (ST-ETEC).

Data analysis

Because multiple pathogens were frequently detected in stool during antibiotic-treated diarrhea episodes, detection of a pathogen alone was not sufficient to assign etiology and attribute antibiotic use. To identify the pathogens responsible for diarrhea treated with antibiotics, we calculated pathogen-specific attributable fractions (AF) of antibiotic-treated diarrhea using generalized linear mixed-effects models (GLMM) that associated pathogen quantity detected with presence in diarrheal versus non-diarrheal stools, as previously.[1] This method leverages the quantity of pathogen detected to identify which is the most likely cause of the diarrhea requiring treatment. The model included sex, test batch, age in quarters, pathogen quantity, pathogen quantity squared, an interaction between pathogen quantity and age, the quantity of the other nine pathogens, a random intercept for individual, and a random slope for site. We calculated episode-specific pathogen attributable fractions as $AFe_i = 1–(1/\mathrm{OR}e_i)$, where $\mathrm{OR}e$ is the pathogen- and quantity-specific odds ratio from the GLMM. Population-level AFs were calculated by summing the attributable fractions per episode (AFes) across all antibiotic-treated episodes, j, i.e. $\left(\frac{1}{j}\right) * \sum_{i=1}^{j} AFe_i$.

We calculated attributable incidence (AI) rates of antibiotic use for each pathogen per 100 child-years as the product of the AF and the total incidence of antibiotic courses for diarrhea identified by surveillance. We also calculated the proportion of all antibiotic use that was attributable to each pathogen as the product of the AF and the proportion of all antibiotic courses that were

For peer review only - http://bmjopen.bmj.com/site/about/guidelines.xhtml

given for diarrhea. To quantify appropriate antibiotic use, we calculated the proportion of pathogen-attributable antibiotic use that was for dysentery. All results were stratified by age, site, and antibiotic drug class.

To assess whether specific pathogens were associated with antibiotic treatment, we estimated risk ratios (RR) for the association between specific pathogens and antibiotic treatment using the pathogen-specific AFe as a continuous exposure. We used the Poisson approximation for log-binomial regression with generalized estimating equations (GEE) to account for repeated episodes within each child. Estimates were scaled to represent the difference between complete attribution (AFe = 1, or the maximum observed AFe for that pathogen if <1) and no attribution. Estimates were adjusted for site, age as a quadratic spline, sex, and the Water, Assets, Maternal Education, Income (WAMI) index, a measure of socioeconomic status.[18]

To further assess whether diarrhea severity mediated the associations with antibiotic treatment, we estimated the total effects of *Shigella* and rotavirus on antibiotic treatment, the pure natural direct effects (PNDE), the total natural indirect effects (TNIE) through the diarrhea severity score and dysentery (*Shigella* only), and the proportions mediated by diarrhea severity and dysentery using the inverse odds ratio weighting approach to mediation analysis with weights truncated at the top 1%.[19,20] The TNIE is the magnitude of the effect of each pathogen on antibiotic use that can be explained by the association of the pathogen with diarrhea severity, while the PNDE describes the remainder of the effect that is not mediated by severity. For the mediation analysis, etiologies were assigned if the pathogen AFe was ≥ 0.5 (i.e. majority attribution). For all analyses, 95% confidence intervals (CI) were estimated by bootstrap with

For peer review only - http://bmjopen.bmj.com/site/about/guidelines.xhtml

1,000 resamples.

Research ethics approval statement

For the parent study, ethical approval was obtained from the Institutional Review Boards at each of the participating research sites and at the University of Virginia School of Medicine (Charlottesville, USA) (14595). For the current study, we obtained ethical approval at the University of Virginia School of Medicine (Charlottesville, USA) (22398) and Emory University (Atlanta, USA) (STUDY00003285).

Patient and public involvement

It was not possible to involve patients or the public in the design, conduct, reporting, or dissemination plans as this was a secondary data analysis of a study conducted in 2009-2014.

RESULTS

These analyses included 1,715 children with 9,392 reported diarrheal episodes and 38,085 (n=6,677 diarrheal, n=31,408 non-diarrheal) stool samples with valid qPCR results for the ten pathogens included (Table 1). Caregivers reported 15,670 antibiotic courses, among which 4,335 courses were associated with treatment of diarrhea. The overall incidence of antibiotic use due to diarrhea was 126.4 courses per 100 child-years, and incidence was higher during the first year of life (134.46 courses per 100 child-years) than the second (118.31 courses per 100 child-years). Higher incidence in younger children reflects higher diarrhea incidence overall, despite a lower proportion of episodes treated with antibiotics in the first year (n=2199/5015, 44.1%) compared

For peer review only - http://bmjopen.bmj.com/site/about/guidelines.xhtml

to the second year (n=2136/4377, 48.7%). Episodes of dysentery accounted for a small proportion of diarrhea episodes (n=461, 4.9%) and antibiotic courses for diarrhea (n=345, 8.0%), despite the fact that 75% of dysentery episodes were treated.

Table 1: Antibiotic use, treatment of diarrhea, and stool sample collection among 1715 children enrolled in the MAL-ED cohort

	**Dhaka, Bangladesh**	**Fortaleza, Brazil**	**Vellore, India**	**Bhaktapur, Nepal**	**Loreto, Peru**	**Naushero Feroze, Pakistan**	**Venda, South Africa**	**Haydom, Tanzania**	**Overall**
Children included[a]	210	165	227	227	194	246	237	209	1715
Total antibiotic courses	3695	224	1740	1059	2041	4922	508	1481	15670
Surveilled diarrheal episodes	1520	168	960	1060	1742	3110	295	537	9392
Antibiotic treatments for diarrhea episodes[b]	897 (59.0%)	18 (10.7%)	242 (25.2%)	319 (30.1%)	688 (39.5%)	1837 (59.1%)	62 (21.0%)	272 (50.7%)	4335 (46.2%)
Penicillin treatment[b]	133 (8.8%)	7 (4.2%)	55 (5.7%)	60 (5.7%)	150 (8.6%)	287 (9.2%)	32 (10.8%)	99 (18.4%)	823 (8.8%)
Sulfonamide treatment[b]	2 (0.1%)	9 (5.4%)	25 (2.6%)	69 (6.5%)	195 (11.2%)	210 (6.8%)	19 (6.4%)	52 (9.7%)	581 (6.2%)
Macrolides treatment[b]	537 (35.3%)	0 (0.0%)	11 (1.1%)	31 (2.9%)	295 (16.9%)	83 (2.7%)	2 (0.7%)	13 (2.4%)	972 (10.3%)
Metronidazole treatment[b]	74 (4.9%)	2 (1.2%)	74 (7.7%)	161 (15.2%)	31 (1.8%)	1185 (38.1%)	6 (2.0%)	125 (23.3%)	1658 (17.7%)
Cephalosporin treatment[b]	77 (5.1%)	1 (0.6%)	88 (9.2%)	45 (4.2%)	33 (1.9%)	575 (18.5%)	1 (0.3%)	2 (0.4%)	822 (8.8%)
Fluoroquinolone treatment[b]	252 (16.6%)	0 (0.0%)	67 (7.0%)	30 (2.8%)	72 (4.1%)	84 (2.7%)	0 (0.0%)	2 (0.4%)	507 (5.4%)
Other antibiotic treatment[b,c]	24 (1.6%)	0 (0.0%)	46 (4.8%)	6 (0.6%)	61 (3.5%)	792 (25.5%)	8 (2.7%)	23 (4.3%)	960 (10.2%)
Surveilled dysentery episodes[b]	65 (4.3%)	4 (2.4%)	60 (6.2%)	48 (4.5%)	101 (5.8%)	101 (3.2%)	11 (3.7%)	71 (13.2%)	461 (4.9%)
Antibiotic treatments for dysentery[d]	51 (5.7%)	2 (11.1%)	27 (11.2%)	41 (12.9%)	86 (12.5%)	82 (4.5%)	4 (6.5%)	52 (19.1%)	345 (8.0%)
Diarrheal stools included in the attribution analysis[b]	1379 (90.7%)	90 (53.6%)	631 (65.7%)	904 (85.3%)	1585 (91.0%)	1815 (58.4%)	115 (39.0%)	158 (29.4%)	6677 (71.1%)
Non-diarrheal stools included in the attribution analysis[e]	3813 (84.2%)	2800 (86.4%)	4498 (88.9%)	4533 (87.8%)	3504 (81.5%)	3896 (80.0%)	4355 (80.7%)	4009 (86.1%)	31408 (84.4%)

Data are n or n (%). Diarrheal and non-diarrheal stools included in this analysis were those that were collected and validly tested for each of the 10 pathogens. [a] Children were included if they had two complete years of follow-up with qPCR data. [b] N=9392. [c] Includes reported tetracyclines, other, and unknown antibiotic use. [d] N=4335. [e] N=37216.

Shigella had the highest incidence of antibiotic use of 14.77 (95% CI: 13.25-16.84) courses per 100 child-years, followed by rotavirus (10.90, 95% CI: 9.75-12.42), sapovirus (10.24, 95% CI: 8.37-12.55), adenovirus 40/41 (9.63, 95% CI: 8.27-11.69), and ST-ETEC (8.56, 95% CI: 7.04-10.71) (Figure 1A, Table S1). *Shigella* was the leading cause of all classes of antibiotic use, except for penicillins, for which attribution was more evenly split across pathogens. Proportionally, *Shigella* and rotavirus were responsible for 11.7% (95% CI: 10.5-13.3) and 8.6% (95% CI: 7.7-9.8) of antibiotic treatments for diarrheal episodes, respectively (Figure 2A, Table S2). These two pathogens were responsible for an even larger total proportion of fluoroquinolone (33.0%) and macrolide (28.0%) use for diarrhea.

For peer review only - http://bmjopen.bmj.com/site/about/guidelines.xhtml

The amount of antibiotic use attributed to specific pathogens varied widely across sites, with more frequent pathogen-attributable use in the South Asian sites compared to African sites. *Shigella* was the leading cause of antibiotic use in India, Nepal, Peru, Pakistan, and South Africa. In contrast, sapovirus was the leading cause in Brazil and Peru, adenovirus 40/41 was the leading cause in Bangladesh, and ST-ETEC was the leading cause in Tanzania (Table S3 and Table S4). Bangladesh was an outlier in terms of frequency; adenovirus 40/41 and *Shigella* were responsible for 50.99 (95% CI: 42.72-62.14) and 45.79 (95% CI: 39.70-54.61) courses per 100 child-years at this site alone, respectively (Figure 1B; Table S5). Of note, while Pakistan had a higher incidence of antibiotic use for diarrhea overall (373.37 per 100 child-years) than Bangladesh (213.57 per 100 child-years), many episodes in Pakistan could not be attributed to the pathogens studied. Rotavirus accounted for a lower proportion of pathogen-attributable antibiotic treatments in Brazil, Peru, and South Africa compared to the other sites (Table S3).

Causes of antibiotic treatment also varied by age. In the first year of life, the pathogens responsible for the highest incidence of antibiotic treatment were rotavirus, adenovirus 40/41, sapovirus, and norovirus, despite antibiotic use being inappropriate for the viral pathogens (Figure S1, Table S6). In the second year of life, the incidence of antibiotic use for *Shigella* was nearly twice that of any other single pathogen.

Diarrhea was the indication for approximately one-quarter (27.7%) of antibiotic treatments overall. Therefore, specific enteric pathogens were responsible for a lower proportion of all antibiotic exposures for any indication. Overall, 3.2% and 2.4% of all antibiotic courses given were attributable to *Shigella* and rotavirus, respectively (Figure 2B; Table S7). Both were

For peer review only - http://bmjopen.bmj.com/site/about/guidelines.xhtml

responsible for a substantial proportion of treatments with specific antibiotic drug classes. 12.2% and 5.5% of fluoroquinolones and macrolides, respectively, were used for treatment of *Shigella*, and 7.1% and 4.0% of fluoroquinolones and macrolides, respectively, were used for treatment of rotavirus. All other pathogens were each responsible for approximately 2% or less of all antibiotic treatments.

Focusing on indicated antibiotic treatments, the highest proportions of antibiotic use for dysentery were attributed to *Shigella* (27.5%) and *C. jejuni/C. coli* (8.5%), respectively (Table S8). These two pathogens accounted for a larger proportion of antibiotic treated dysentery episodes compared to antibiotic treated watery diarrhea episodes (17.2% and 5.3% more, respectively). However, less than a fifth of all antibiotic treatments attributable to *Shigella* (18.7%) and *C. jejuni/C. coli* (18.6%) were for dysentery. The attributable fractions of antibiotic treatments for dysentery compared to watery diarrhea did not differ for the other pathogens, and less than 10% of antibiotic treatments attributed to the other pathogens were for the treatment of dysentery.

After adjustment for age, site, sex, and socioeconomic status, *Shigella*-attributable diarrhea episodes were 46% more likely to be treated with antibiotics compared to all other episodes (adjusted risk ratio (aRR): 1.46, 95% CI: 1.33-1.60), and rotavirus-attributable episodes were 19% more likely to be treated (1.19, 95% CI: 1.09-1.31) (Figure 3). The associations were stronger for key drug classes; *Shigella*-attributable diarrhea episodes were 49% more likely to be treated with fluoroquinolones or macrolides compared to other episodes (1.49, 95% CI: 1.28-1.73), and rotavirus-attributable episodes were 21% more likely to be treated (1.21, 95% CI:

For peer review only - http://bmjopen.bmj.com/site/about/guidelines.xhtml

1.04-1.41). The associations between *Shigella* and rotavirus and antibiotic treatment were consistent across most sites, excluding Tanzania and Nepal (Table S9). Uniquely, *Cryptosporidium* was strongly associated with antibiotic treatment in Tanzania (aRR: 3.18, 95% CI: 1.36-7.43) and India (aRR: 2.11, 95% CI: 1.18-3.79).

Diarrhea severity and dysentery mediated 5% and 18% of the association between antibiotic treatment and *Shigella*, respectively (Table S10). When considered together, these two factors mediated a total 26% of the antibiotic treatment association and 48% of the fluoroquinolone and macrolide treatment association with *Shigella* (Table 2). Similarly, diarrhea severity mediated 44% of the association between rotavirus and antibiotic treatment and 53% of the association with fluoroquinolone and macrolide treatment.

Table 2. Assessment of whether diarrhea severity and dysentery mediated the relationship between *Shigella* and rotavirus diarrhea and antibiotic treatment among 1715 children in the MAL-ED cohort

	Shigella (Mediated by diarrhea severity and dysentery)		Rotavirus (Mediated by diarrhea severity)	
	Any antibiotic	Fluoroquinolones or macrolides	Any antibiotic	Fluoroquinolones or macrolides
Total Effect Rate Ratio	1.30 (1.21, 1.40)	1.39 (1.21, 1.58)	1.10 (1.01, 1.19)	1.17 (1.01, 1.33)
Pure Natural Direct Effect Rate Ratio	1.22 (1.12, 1.34)	1.20 (1.02, 1.40)	1.06 (0.97, 1.15)	1.08 (0.89, 1.27)
Total Natural Indirect Effect Rate Ratio	1.07 (1.00, 1.12)	1.16 (1.05, 1.28)	1.04 (0.98, 1.10)	1.08 (0.96, 1.24)
Proportion Mediated	0.26 (0.02, 0.50)	0.48 (0.16, 0.90)	0.44 (0.00, 1.00)	0.53 (0.00, 1.00)

Data are risk ratios (RR) with 95% confidence intervals (CIs). Note: The Total Effect Rate Ratio for *Shigella* and rotavirus do not equal the total effects in Figure 3 as the attributable fractions per episode (AFe) were dichotomized > 0.5 for the mediation models, but left continuous in Figure 3.

DISCUSSION

Because diarrhea was responsible for more than a quarter of antibiotic treatments in the MAL-ED study, interventions that target specific enteric pathogens could reduce antibiotic selection pressure and make an important contribution to efforts to combat AMR. We found that *Shigella* and rotavirus were the top causes of antibiotic treatment for diarrhea, with more than two in every ten children on average exposed to antibiotics due to each of these pathogens in the first

For peer review only - http://bmjopen.bmj.com/site/about/guidelines.xhtml

two years of life. Furthermore, *Shigella* was responsible for the most uses of fluoroquinolones and macrolides, which are first line therapies for *Campylobacter, Shigella,* and diarrheagenic *E. coli*. While the frequency of antibiotic treatment varied by an order of magnitude across settings, *Shigella* and rotavirus were among the leading causes at all sites. Notably, rotavirus was a less frequent cause of antibiotic use in the three sites (Brazil, Peru, and South Africa) that had introduced rotavirus vaccine prior to the study. Rotavirus vaccine coverage is high (>70%) and availability has expanded to all countries included in the MAL-ED study (excluding Bangladesh),[21,22] suggesting rotavirus vaccine could substantially reduce unnecessary use of antibiotics.

These results are consistent with a similar analysis of facility-ascertained moderate-to-severe diarrhea conducted in GEMS,[4] but have broader implications since they include antibiotic treatments for diarrhea episodes identified in the community and therefore report much higher rates of antibiotic treated diarrhea. In LMICs, where the majority of antibiotic use occurs outside of medically attended care, estimates of antibiotic use from healthcare settings alone are large underestimates of the total burden. This analysis also provides a broader context by considering antibiotic treatments for all indications beyond diarrhea, which is important for LMIC settings which have high burdens of respiratory illnesses and other infections as well.

The contribution of most enteric pathogens to antibiotic use was in proportion to their contribution to diarrhea overall. However, in addition to being the leading causes of diarrhea in the first and second years of life, respectively, rotavirus and *Shigella* were disproportionately more responsible for antibiotic use than would have been expected based on the age-specific

For peer review only - http://bmjopen.bmj.com/site/about/guidelines.xhtml

incidence of disease. Because point-of-care diagnostics were not available, treatment decisions were not made based on known etiology but were rather likely due to unique features of the clinical syndromes caused by these pathogens. Indeed, we found evidence that the associations between *Shigella* and rotavirus and antibiotic treatment could be explained by the fact that these pathogens cause more severe disease. Unsurprisingly, since *Shigella* is the leading cause of dysentery for which treatment is recommended, dysentery also mediated the relationship between *Shigella* and antibiotic use. Because diarrhea severity and dysentery only explained a portion of the relationships, there may be other subjective indicators for treatment that were insufficiently captured by the severity metrics captured.

While the contribution of individual enteric pathogens to total antibiotic use was limited (<5% for each pathogen), reductions of these magnitudes would be comparable or larger than the effect of most existing antibiotic stewardship interventions.[23] Furthermore, the attributable proportions increased considerably for fluoroquinolones and macrolides, which are the first-line classes for diarrhea treatment and important oral antibiotic options for a broad range of community-acquired infections. For example, *Shigella* was responsible for approximately 1 in 8 uses of fluoroquinolones and 1 in 18 uses of macrolides. *Shigella* vaccines in development[24,25] could provide an opportunity to reduce this use. Importantly, enteric viruses accounted for a quarter of all fluoroquinolone use and 16% of macrolide use. These treatment courses were not indicated and represent the burden of antibiotic overuse that could be potentially prevented by vaccines or other pathogen-specific interventions.

Interventions that reduce the incidence of bacterial diarrhea episodes requiring antibiotics,

For peer review only - http://bmjopen.bmj.com/site/about/guidelines.xhtml

particularly due to *Shigella* and *Campylobacter,* would also have the direct benefit of potentially preventing antibiotic-resistant disease. *Shigella* and *Campylobacter* are on the WHO priority pathogens list for research and development of new antibiotics due to increasing AMR.[26] While antibiotic resistance testing was not conducted in MAL-ED, some of the treated episodes may have been resistant to fluoroquinolones and/or macrolides, as has been reported particularly in Asia and Africa.[27–29] Specifically, a review by Gu and colleagues found that resistance to nalidixic acid and ciprofloxacin in *Shigella* spp. was 65% and 29%, respectively, in Asia and Africa in 2007-2009. Moreover, resistance rates were higher amongst children with diarrheal illnesses than adults (33.0% vs. 14.3% resistance to nalidixic acid and 7.5% vs. 3.6% resistance to ciprofloxacin).[27] Ghunaim et al. found similar results regarding resistance to ciprofloxacin (fluoroquinolone) and erythromycin (macrolide) in *Campylobacter* in individuals from Asia and Africa who presented to care in Qatar. Nearly three-quarters and two-thirds of individuals from Asia and Africa, respectively, were infected with *Campylobacter* isolates resistant to ciprofloxacin, while a smaller percentage were resistant to erythromycin (7.1% in Asia vs. 14.3% in Africa).[28]

Finally, because subclinical carriage of these and other bacterial enteropathogens is highly common among young children in LMICs,[30] reductions in antibiotic use overall, including treatments of viral diarrhea, would have the important ancillary benefit of preventing antibiotic exposure to bacteria present as subclinical infections. This type of antibiotic exposure has been described as "bystander selection," or the selective pressure for resistance on pathogens that are not the target of treatment.[31] *Shigella* and *Campylobacter* were detected in 10% and 28% of all non-diarrheal stools collected in MAL-ED[30], respectively, suggesting that these pathogens were

For peer review only - http://bmjopen.bmj.com/site/about/guidelines.xhtml

likely frequently exposed to antibiotics due to diarrhea treatment.

Because prescriptions and/or caregiver-reported indications for treatment were unavailable, this analysis was limited by attributing antibiotic use to diarrhea based on the temporal overlap of symptoms. Furthermore, information on specific drug given and dosing were not available, and antibiotic courses were defined based on antibiotic-free days rather than the intended duration.

The evidence that *Shigella* and rotavirus were disproportionately responsible for antibiotic use due to their high burden and severity strengthens the value proposition for rotavirus and *Shigella* vaccines[10] and other pathogen-specific interventions. These strategies could complement more generalized interventions such as educational campaigns focused on antibiotic stewardship. Prevention of diarrheal disease offers an important opportunity to reduce both antibiotic use and overuse.

For peer review only

DECLARATIONS

Author affiliations

[1] Division of Infectious Diseases & International Health, School of Medicine, University of Virginia, 345 Crispell Drive, Charlottesville, VA, 22908, USA;

[2] Division of Epidemiology, School of Public Health, University of California - Berkeley, 2121 Berkeley Way, Berkeley, California, 94720, USA;

[3] Department of Epidemiology, Rollins School of Public Health, Emory University, 1518 Clifton Road NE, Atlanta, GA, 30322, USA

For peer review only

Contributors

SAB led data analysis, interpretation, visualization, and writing of the report. JAP-M led and conceptualized the data analysis and contributed to the interpretation, and reviewing/editing the report. JAL contributed to interpretation, and reviewing/editing the report. JL led the development of the laboratory assays and contributed to reviewing/editing the report. ERH led funding acquisition and administration of the parent study, and contributed to reviewing/editing the report. ETRM led the conceptualization, methodology, funding acquisition, writing of the report and contributed to data analysis, interpretation, and visualization. All authors read and approved the final manuscript.

Funding

This work was supported by Wellcome (219741/Z/19/Z to ETRM). The Etiology, Risk Factors and Interactions of Enteric Infections and Malnutrition and the Consequences for Child Health and Development Project (MAL-ED) was a collaborative project supported by the Bill &

For peer review only - http://bmjopen.bmj.com/site/about/guidelines.xhtml

Melinda Gates Foundation (OPP1131125), the Foundation for the NIH, the National Institutes of Health, and the Fogarty International Center.

Competing interests

The authors declare that they have no competing interests.

Patient consent for publication

Patient consent was not required for this secondary data analysis. In the parent study, informed and signed consent was obtained from the guardian of each child.

Data availability statement

De-identified participant data from the MAL-ED study is publicly available at ClinEpiDB.org after approval of a proposal by the study PIs.

For peer review only

For peer review only - http://bmjopen.bmj.com/site/about/guidelines.xhtml

REFERENCES

1. Platts-Mills JA, Liu J, Rogawski ET, et al. Use of quantitative molecular diagnostic methods to assess the aetiology, burden, and clinical characteristics of diarrhoea in children in low-resource settings: a reanalysis of the MAL-ED cohort study. *The Lancet Global Health*. 2018;6(12):e1309-e1318. doi:10.1016/S2214-109X(18)30349-8

2. Rogawski ET, Platts-Mills JA, Seidman JC, et al. Use of antibiotics in children younger than two years in eight countries: a prospective cohort study. *Bulletin of the World Health Organization*. 2017;95(1):49-61.

3. World Health Organization. *WHO Recommendations on the Management of Diarrhoea and Pneumonia in HIV-Infected Infants and Children: Integrated Management of Childhood Illness (IMCI)*. World Health Organization; 2010. Accessed May 4, 2021. https://www.ncbi.nlm.nih.gov/books/NBK305340/

4. Lewnard JA, McQuade ETR, Platts-Mills JA, Kotloff KL, Laxminarayan R. Incidence and etiology of clinically-attended, antibiotic-treated diarrhea among children under five years of age in low- and middle-income countries: Evidence from the Global Enteric Multicenter Study. *PLOS Neglected Tropical Diseases*. 2020;14(8):e0008520. doi:10.1371/journal.pntd.0008520

5. World Health Organization. WHO publishes list of bacteria for which new antibiotics are urgently needed. Published February 27, 2017. Accessed June 4, 2021. https://www.who.int/news/item/27-02-2017-who-publishes-list-of-bacteria-for-which-new-antibiotics-are-urgently-needed

6. Abdulah R. Antibiotic Abuse in Developing Countries. *Pharmaceutical Regulatory Affairs: Open Access*. 2012;1:e106. doi:10.4172/2167-7689.1000e106

7. Bebell LM, Muiru AN. Antibiotic Use and Emerging Resistance: How Can Resource-Limited Countries Turn the Tide? *Global Heart*. 2014;9(3):347-358. doi:10.1016/j.gheart.2014.08.009

8. Mittal SK, Mathew JL. Regulating the Use of Drugs in Diarrhea. *Journal of Pediatric Gastroenterology and Nutrition*. 2001;33:S26.

9. Jansen KU, Knirsch C, Anderson AS. The role of vaccines in preventing bacterial antimicrobial resistance. *Nat Med*. 2018;24(1):10-19. doi:10.1038/nm.4465

10. Lipsitch M, Siber GR. How Can Vaccines Contribute to Solving the Antimicrobial Resistance Problem? *mBio*. 2016;7(3):e00428-16. doi:10.1128/mBio.00428-16

11. Buckley BS, Henschke N, Bergman H, et al. Impact of vaccination on antibiotic usage: a systematic review and meta-analysis. *Clinical Microbiology and Infection*. 2019;25(10):1213-1225. doi:10.1016/j.cmi.2019.06.030

For peer review only - http://bmjopen.bmj.com/site/about/guidelines.xhtml

12. Lewnard JA, Fries LF, Cho I, Chen J, Laxminarayan R. Prevention of antimicrobial prescribing among infants following maternal vaccination against respiratory syncytial virus. *medRxiv*. Published online in clearance 2021.

13. Lewnard JA, Lo NC, Arinaminpathy N, Frost I, Laxminarayan R. Childhood vaccines and antibiotic use in low- and middle-income countries. *Nature*. 2020;581(7806):94-99. doi:10.1038/s41586-020-2238-4

14. Cohen D, Muhsen K. Vaccines for enteric diseases. *Human Vaccines & Immunotherapeutics*. 2019;15(6):1205-1214. doi:10.1080/21645515.2019.1611200

15. The MAL-ED Network Investigators. The MAL-ED Study: A Multinational and Multidisciplinary Approach to Understand the Relationship Between Enteric Pathogens, Malnutrition, Gut Physiology, Physical Growth, Cognitive Development, and Immune Responses in Infants and Children Up to 2 Years of Age in Resource-Poor Environments. *Clinical Infectious Diseases*. 2014;59(suppl_4):S193-S206. doi:10.1093/cid/ciu653

16. Platts-Mills JA, Babji S, Bodhidatta L, et al. Pathogen-specific burdens of community diarrhoea in developing countries: a multisite birth cohort study (MAL-ED). *The Lancet Global Health*. 2015;3(9):e564-e575.

17. Liu J, Kabir F, Manneh J, et al. Development and assessment of molecular diagnostic tests for 15 enteropathogens causing childhood diarrhoea: a multicentre study. *The Lancet Infectious Diseases*. 2014;14(8):716-724. doi:10.1016/S1473-3099(14)70808-4

18. Psaki SR, Seidman JC, Miller M, et al. Measuring socioeconomic status in multicountry studies: results from the eight-country MAL-ED study. *Popul Health Metr*. 2014;12(1):8. doi:10.1186/1478-7954-12-8

19. Shi B, Choirat C, Valeri L. CMAverse: a suite of functions for causal mediation analysis. https://bs1125.github.io/CMAverse/

20. Nguyen QC, Osypuk TL, Schmidt NM, Glymour MM, Tchetgen Tchetgen EJ. Practical guidance for conducting mediation analysis with multiple mediators using inverse odds ratio weighting. *Am J Epidemiol*. 2015;181(5):349-356. doi:10.1093/aje/kwu278

21. GHO | By category | Rotavirus - Immunization coverage estimates by country. WHO. Accessed February 23, 2022. https://apps.who.int/gho/data/node.main.ROTACn

22. Nepal introduces Rota virus vaccine against diarrhoea in children: National Immunization Programme achieves new milestone. Accessed February 23, 2022. https://www.who.int/nepal/news/detail/02-07-2020-nepal-introduces-rota-virus-vaccine-against-diarrhoea-in-children-national-immunization-programme-achieves-new-milestone

23. Hallsworth M, Chadborn T, Sallis A, et al. Provision of social norm feedback to high prescribers of antibiotics in general practice: a pragmatic national randomised controlled trial. *The Lancet*. 2016;387(10029):1743-1752. doi:10.1016/S0140-6736(16)00215-4

For peer review only - http://bmjopen.bmj.com/site/about/guidelines.xhtml

24. Riddle MS, Chen WH, Kirkwood CD, MacLennan CA. Update on vaccines for enteric pathogens. *Clinical Microbiology and Infection*. 2018;24(10):1039-1045. doi:10.1016/j.cmi.2018.06.023

25. Talaat KR, Alaimo C, Martin P, et al. Human challenge study with a Shigella bioconjugate vaccine: Analyses of clinical efficacy and correlate of protection. *EBioMedicine*. 2021;66:103310. doi:10.1016/j.ebiom.2021.103310

26. Tacconelli E, Magrini N. Global priority list of antibiotic-resistant bacteria to guide research, discovery, and development of new antibiotics. Published April 8, 2021. https://www.who.int/medicines/publications/WHO-PPL-Short_Summary_25Feb-ET_NM_WHO.pdf

27. Gu B, Cao Y, Pan S, et al. Comparison of the prevalence and changing resistance to nalidixic acid and ciprofloxacin of Shigella between Europe-America and Asia-Africa from 1998 to 2009. *Int J Antimicrob Agents*. 2012;40(1):9-17. doi:10.1016/j.ijantimicag.2012.02.005

28. Ghunaim H, Behnke JM, Aigha I, et al. Analysis of Resistance to Antimicrobials and Presence of Virulence/Stress Response Genes in Campylobacter Isolates from Patients with Severe Diarrhoea. *PLOS ONE*. 2015;10(3):e0119268. doi:10.1371/journal.pone.0119268

29. Mahbubur R, Shoma S, Rashid H, et al. Increasing Spectrum in Antimicrobial Resistance of Shigella Isolates in Bangladesh: Resistance to Azithromycin and Ceftriaxone and Decreased Susceptibility to Ciprofloxacin. *J Health Popul Nutr*. 2007;25(2):158-167.

30. Rogawski ET, Liu J, Platts-Mills JA, et al. Use of quantitative molecular diagnostic methods to investigate the effect of enteropathogen infections on linear growth in children in low-resource settings: longitudinal analysis of results from the MAL-ED cohort study. *The Lancet Global Health*. 2018;6(12):e1319-e1328. doi:10.1016/S2214-109X(18)30351-6

31. Tedijanto C, Olesen SW, Grad YH, Lipsitch M. Estimating the proportion of bystander selection for antibiotic resistance among potentially pathogenic bacterial flora. *PNAS*. 2018;115(51):E11988-E11995.

For peer review only - http://bmjopen.bmj.com/site/about/guidelines.xhtml

Figure 1. Attributable incidence of pathogen-specific antibiotic courses for diarrhea by antibiotic drug class (A) and by site (B) among 1715 children in the MAL-ED cohort. Error bars show 95% CI. *C. jejuni/C. coli* = *Campylobacter jejuni/Campylobacter coli*. ST-ETEC= heat-stable enterotoxigenic *Escherichia coli*. tEPEC = typical enteropathogenic *Escherichia coli*.

Figure 2. Pathogen-specific attributable fractions of antibiotic courses for diarrhea (A) and for all indications (B) by antibiotic drug class among 1715 children in the MAL-ED cohort. Error bars show 95% CI. *C. jejuni/C. coli* = *Campylobacter jejuni/Campylobacter coli*. ST-ETEC= heat-stable enterotoxigenic *Escherichia coli*. tEPEC = typical enteropathogenic *Escherichia coli*.

Figure 3. Associations between specific diarrhea etiologies and treatment with any antibiotics and fluoroquinolones or macrolides among 1715 children in the MAL-ED cohort. Estimates are risk ratios adjusted for age, sex, socioeconomic status, and site. Error bars show 95% CI. *C. jejuni/C. coli* = *Campylobacter jejuni/Campylobacter coli*. ST-ETEC= heat-stable enterotoxigenic *Escherichia coli*. tEPEC = typical enteropathogenic *Escherichia coli*.

For peer review only

For peer review only - http://bmjopen.bmj.com/site/about/guidelines.xhtml

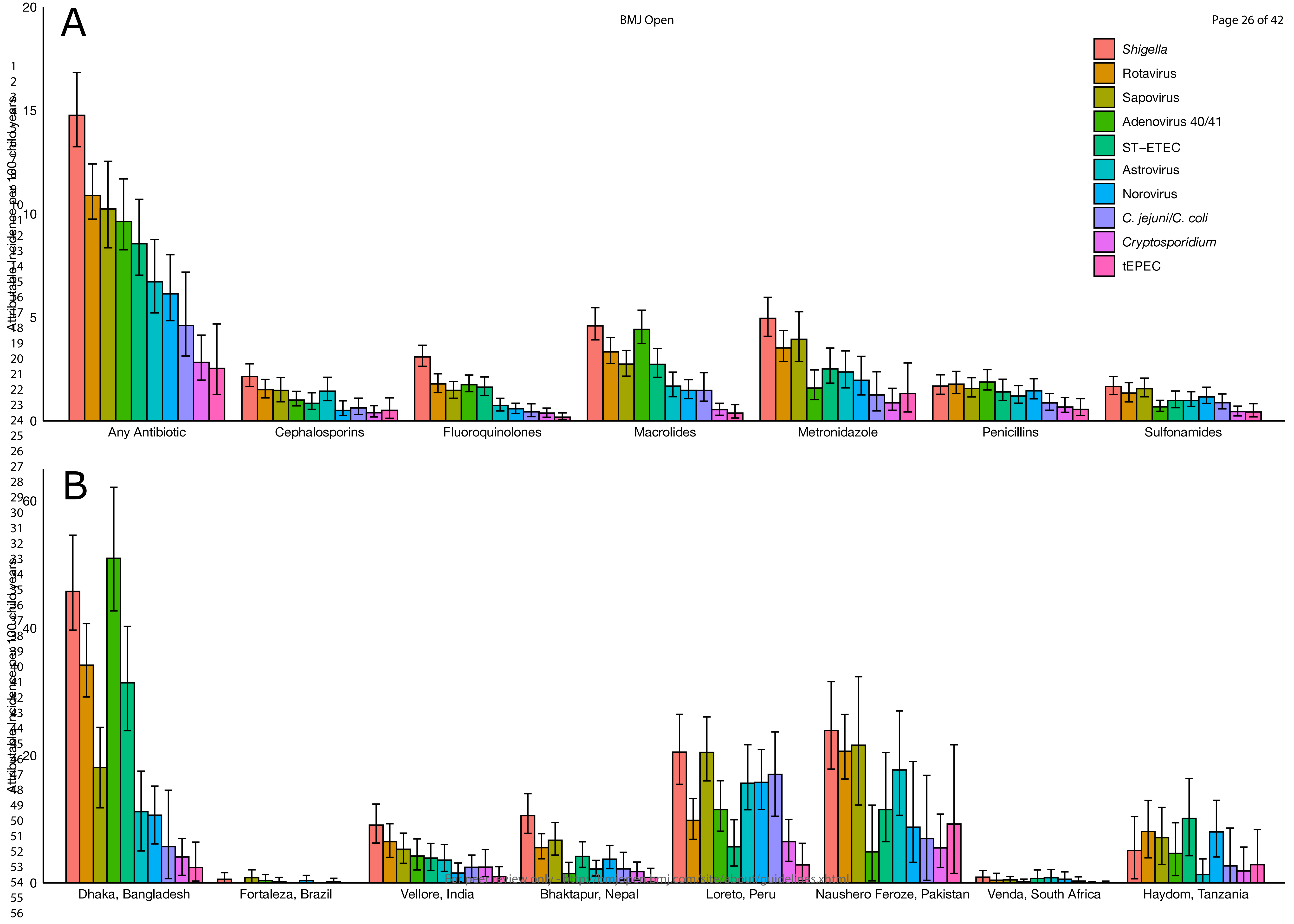
A
Shigella
Rotavirus
Sapovirus
Adenovirus 40/41
ST−ETEC
Astrovirus
Norovirus
C. jejuni/C. coli
Cryptosporidium
tEPEC
Attributable Incidence per 100 child years
20
15
10
5
0
Any Antibiotic
Cephalosporins
Fluoroquinolones
Macrolides
Metronidazole
Penicillins
Sulfonamides
B
Attributable Incidence per 100 child years
60
40
20
0
Dhaka, Bangladesh
Fortaleza, Brazil
Vellore, India
Bhaktapur, Nepal
Loreto, Peru
Naushero Feroze, Pakistan
Venda, South Africa
Haydom, Tanzania

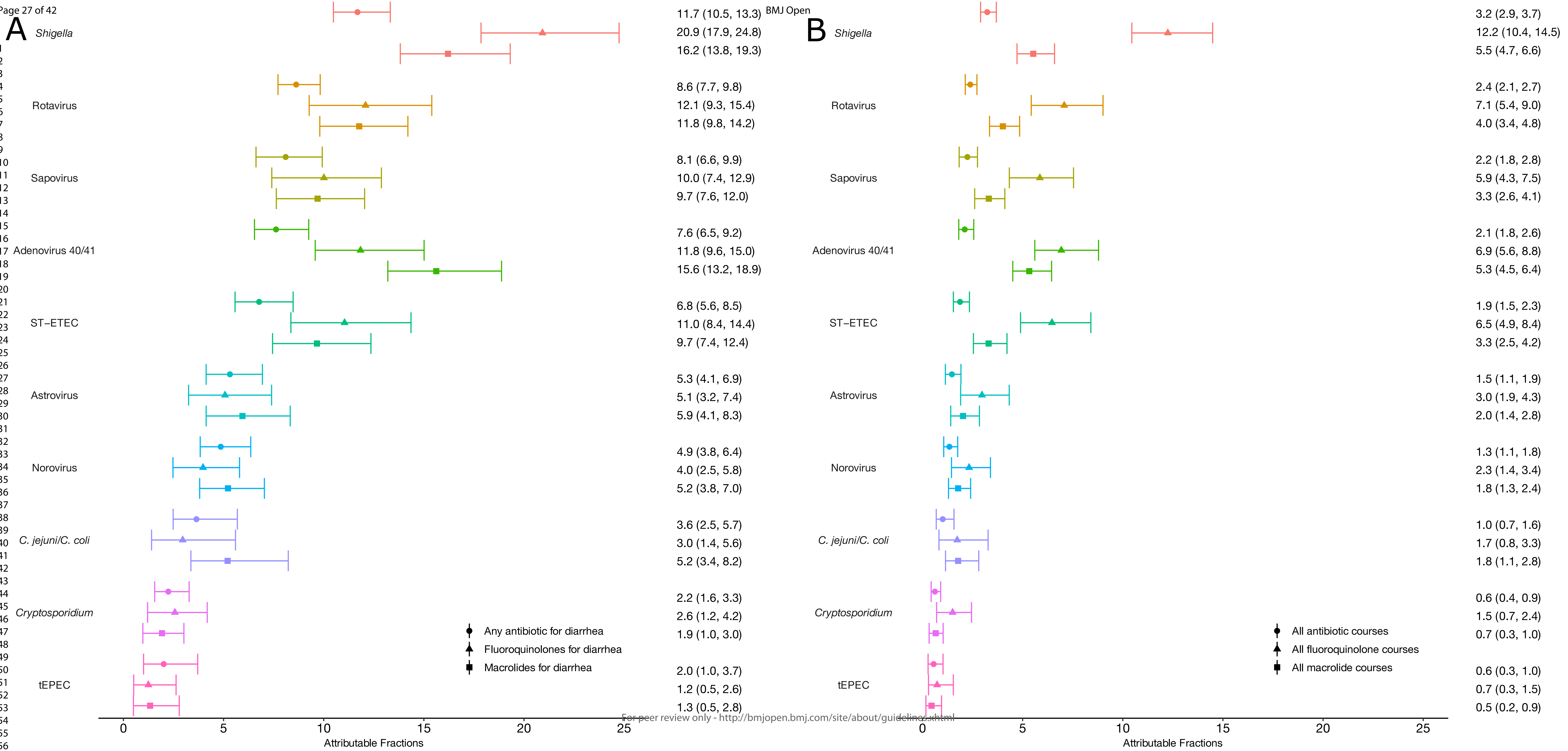
A
Shigella
11.7 (10.5, 13.3)
20.9 (17.9, 24.8)
16.2 (13.8, 19.3)
Rotavirus
8.6 (7.7, 9.8)
12.1 (9.3, 15.4)
11.8 (9.8, 14.2)
Sapovirus
8.1 (6.6, 9.9)
10.0 (7.4, 12.9)
9.7 (7.6, 12.0)
Adenovirus 40/41
7.6 (6.5, 9.2)
11.8 (9.6, 15.0)
15.6 (13.2, 18.9)
ST–ETEC
6.8 (5.6, 8.5)
11.0 (8.4, 14.4)
9.7 (7.4, 12.4)
Astrovirus
5.3 (4.1, 6.9)
5.1 (3.2, 7.4)
5.9 (4.1, 8.3)
Norovirus
4.9 (3.8, 6.4)
4.0 (2.5, 5.8)
5.2 (3.8, 7.0)
C. jejuni/C. coli
3.6 (2.5, 5.7)
3.0 (1.4, 5.6)
5.2 (3.4, 8.2)
Cryptosporidium
2.2 (1.6, 3.3)
2.6 (1.2, 4.2)
1.9 (1.0, 3.0)
tEPEC
2.0 (1.0, 3.7)
1.2 (0.5, 2.6)
1.3 (0.5, 2.8)
Any antibiotic for diarrhea
Fluoroquinolones for diarrhea
Macrolides for diarrhea
0
5
10
15
20
25
Attributable Fractions
B
Shigella
3.2 (2.9, 3.7)
12.2 (10.4, 14.5)
5.5 (4.7, 6.6)
Rotavirus
2.4 (2.1, 2.7)
7.1 (5.4, 9.0)
4.0 (3.4, 4.8)
Sapovirus
2.2 (1.8, 2.8)
5.9 (4.3, 7.5)
3.3 (2.6, 4.1)
Adenovirus 40/41
2.1 (1.8, 2.6)
6.9 (5.6, 8.8)
5.3 (4.5, 6.4)
ST–ETEC
1.9 (1.5, 2.3)
6.5 (4.9, 8.4)
3.3 (2.5, 4.2)
Astrovirus
1.5 (1.1, 1.9)
3.0 (1.9, 4.3)
2.0 (1.4, 2.8)
Norovirus
1.3 (1.1, 1.8)
2.3 (1.4, 3.4)
1.8 (1.3, 2.4)
C. jejuni/C. coli
1.0 (0.7, 1.6)
1.7 (0.8, 3.3)
1.8 (1.1, 2.8)
Cryptosporidium
0.6 (0.4, 0.9)
1.5 (0.7, 2.4)
0.7 (0.3, 1.0)
tEPEC
0.6 (0.3, 1.0)
0.7 (0.3, 1.5)
0.5 (0.2, 0.9)
All antibiotic courses
All fluoroquinolone courses
All macrolide courses
0
5
10
15
20
25
Attributable Fractions

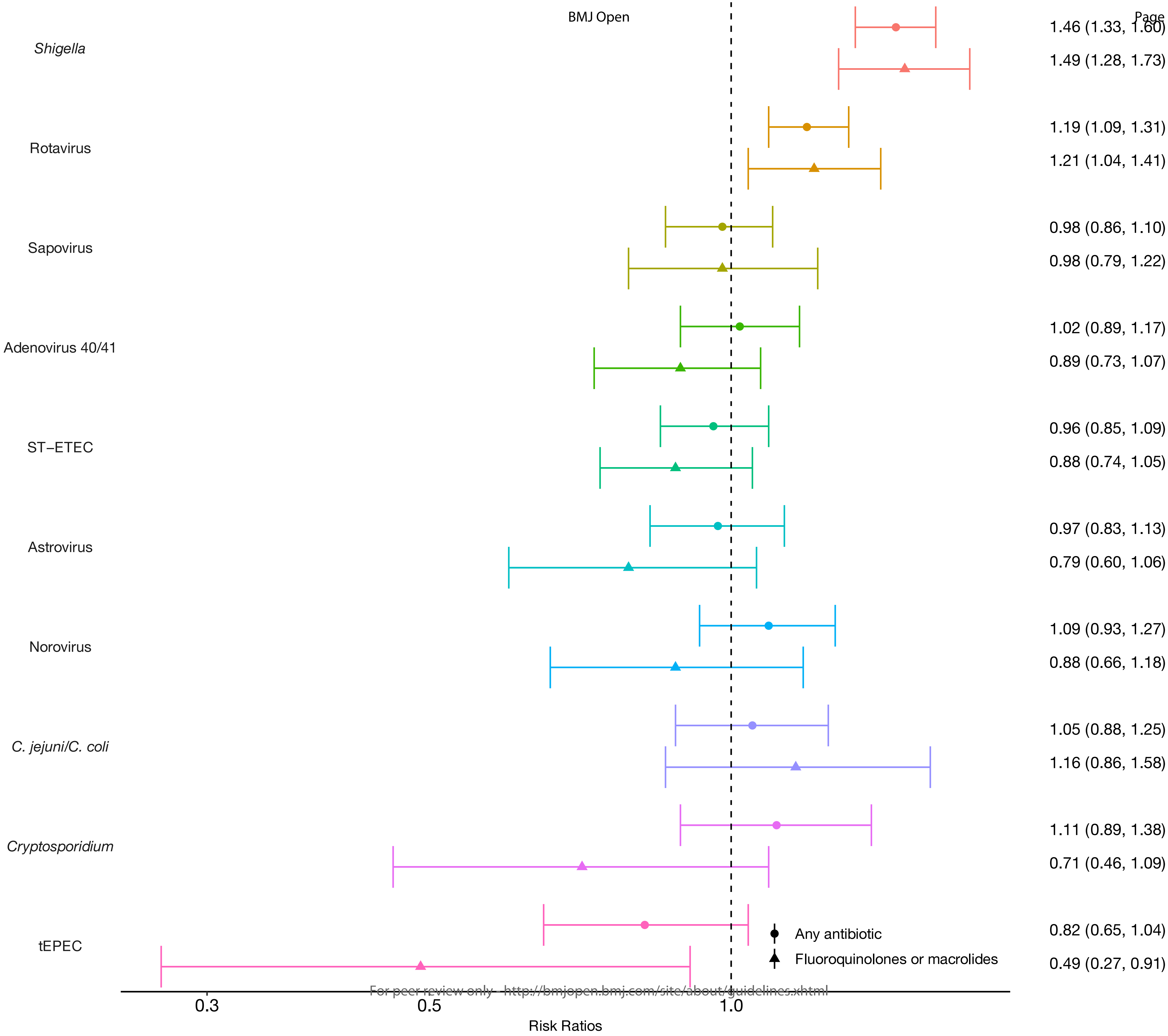

For peer review only - http://bmjopen.bmj.com/site/about/guidelines.xhtml

Supplementary Online Content

Antibiotic use attributable to etiology-specific diarrhea in children under two years of age in low-resource settings

Stephanie A Brennhofer, James A Platts-Mills, Joseph A Lewnard, Jie Liu, Eric R Houpt, Elizabeth T Rogawski McQuade

For peer review only

For peer review only - http://bmjopen.bmj.com/site/about/guidelines.xhtml

Table S1. Attributable incidence of pathogen-specific antibiotic courses for diarrhea by antibiotic drug class among 1715 children in the MAL-ED cohort.

	Any antibiotic	Cephalosporins	Fluoroquinolones	Macrolides	Metronidazole	Penicillins	Sulfonamides
Shigella	14.77 (13.25, 16.84)	2.14 (1.66, 2.76)	3.09 (2.64, 3.66)	4.59 (3.92, 5.47)	4.96 (4.09, 5.97)	1.69 (1.29, 2.23)	1.66 (1.27, 2.15)
Rotavirus	10.90 (9.75, 12.42)	1.51 (1.11, 2.01)	1.79 (1.37, 2.28)	3.33 (2.78, 4.02)	3.53 (2.86, 4.37)	1.78 (1.32, 2.40)	1.35 (0.92, 1.85)
Sapovirus	10.24 (8.37, 12.55)	1.47 (0.93, 2.10)	1.48 (1.09, 1.90)	2.75 (2.16, 3.41)	3.95 (2.87, 5.28)	1.57 (1.16, 2.09)	1.56 (1.17, 2.07)
Adenovirus 40/41	9.63 (8.27, 11.69)	1.01 (0.73, 1.43)	1.75 (1.42, 2.22)	4.43 (3.74, 5.35)	1.59 (1.03, 2.47)	1.88 (1.49, 2.48)	0.68 (0.46, 1.00)
ST-ETEC	8.56 (7.04, 10.71)	0.85 (0.56, 1.36)	1.63 (1.24, 2.12)	2.74 (2.11, 3.50)	2.51 (1.83, 3.53)	1.40 (0.99, 2.01)	0.99 (0.64, 1.44)
Astrovirus	6.72 (5.22, 8.77)	1.44 (0.98, 2.11)	0.75 (0.48, 1.09)	1.68 (1.17, 2.36)	2.36 (1.60, 3.38)	1.20 (0.86, 1.71)	0.99 (0.70, 1.41)
Norovirus	6.14 (4.85, 8.03)	0.51 (0.26, 0.97)	0.59 (0.37, 0.86)	1.48 (1.08, 1.99)	1.97 (1.26, 3.12)	1.45 (1.07, 2.04)	1.16 (0.85, 1.63)
C. jejuni/C. coli	4.61 (3.14, 7.19)	0.63 (0.32, 1.10)	0.44 (0.21, 0.83)	1.47 (0.95, 2.33)	1.25 (0.48, 2.37)	0.87 (0.52, 1.33)	0.88 (0.58, 1.31)
Cryptosporidium	2.83 (1.97, 4.15)	0.39 (0.19, 0.73)	0.38 (0.18, 0.62)	0.55 (0.27, 0.86)	0.87 (0.52, 1.58)	0.67 (0.42, 1.13)	0.45 (0.24, 0.71)
tEPEC	2.54 (1.27, 4.69)	0.51 (0.13, 1.11)	0.18 (0.08, 0.39)	0.38 (0.14, 0.79)	1.32 (0.43, 2.80)	0.56 (0.26, 1.08)	0.43 (0.20, 0.83)

Data are attributable incidence rates per 100 child years with 95% confidence intervals (CIs). These data also reported in Figure 1A. Abbreviations: *C. jejuni/C. coli = Campylobacter jejuni/Campylobacter coli*. ST-ETEC= heat-stable enterotoxigenic *Escherichia coli*. tEPEC = typical enteropathogenic *Escherichia coli*.

For peer review only

Table S2. Pathogen-specific attributable fractions of antibiotic courses for diarrhea by antibiotic drug class among 1715 children in the MAL-ED cohort.

	Any Antibiotic	Cephalosporins	Fluoroquinolones	Macrolides	Metronidazole	Penicillins	Sulfonamides
Shigella	11.7 (10.5, 13.3)	8.9 (6.9, 11.5)	20.9 (17.9, 24.7)	16.2 (13.8, 19.3)	10.3 (8.5, 12.4)	7.0 (5.4, 9.3)	9.8 (7.5, 12.7)
Rotavirus	8.6 (7.7, 9.8)	6.3 (4.6, 8.4)	12.1 (9.3, 15.4)	11.8 (9.8, 14.2)	7.3 (5.9, 9.0)	7.4 (5.5, 10.0)	8.0 (5.4, 10.9)
Sapovirus	8.1 (6.6, 9.9)	6.2 (3.9, 8.8)	10.0 (7.4, 12.9)	9.7 (7.6, 12.0)	8.2 (5.9, 10.9)	6.5 (4.8, 8.7)	9.2 (6.9, 12.2)
Adenovirus 40/41	7.6 (6.5, 9.3)	4.2 (3.0, 6.0)	11.8 (9.6, 15.0)	15.6 (13.2, 18.9)	3.3 (2.1, 5.1)	7.8 (6.2, 10.3)	4.0 (2.7, 5.9)
ST-ETEC	6.8 (5.6, 8.5)	3.6 (2.3, 5.7)	11.0 (8.4, 14.4)	9.7 (7.4, 12.4)	5.2 (3.8, 7.3)	5.8 (4.1, 8.4)	5.8 (3.8, 8.5)
Astrovirus	5.3 (4.1, 6.9)	6.0 (4.1, 8.8)	5.1 (3.3, 7.4)	5.9 (4.1, 8.3)	4.9 (3.3, 7.0)	5.0 (3.6, 7.1)	5.9 (4.2, 8.3)
Norovirus	4.9 (3.8, 6.4)	2.1 (1.1, 4.1)	4.0 (2.5, 5.8)	5.2 (3.8, 7.0)	4.1 (2.6, 6.5)	6.1 (4.4, 8.5)	6.8 (5.0, 9.6)
C. jejuni/C. coli	3.6 (2.5, 5.7)	2.6 (1.3, 4.6)	3.0 (1.4, 5.6)	5.2 (3.4, 8.2)	2.6 (1.0, 4.9)	3.6 (2.2, 5.5)	5.2 (3.4, 7.7)
Cryptosporidium	2.2 (1.6, 3.3)	1.6 (0.8, 3.1)	2.6 (1.2, 4.2)	1.9 (1.0, 3.0)	1.8 (1.1, 3.3)	2.8 (1.7, 4.7)	2.6 (1.4, 4.2)
tEPEC	2.0 (1.0, 3.7)	2.1 (0.5, 4.6)	1.2 (0.5, 2.6)	1.3 (0.5, 2.8)	2.7 (0.9, 5.8)	2.3 (1.1, 4.5)	2.6 (1.2, 4.9)

Data are attributable fraction percentages with 95% confidence intervals (CIs). These data also reported in Figure 2A. Abbreviations: *C. jejuni/C. coli* = *Campylobacter jejuni/Campylobacter coli*. ST-ETEC= heat-stable enterotoxigenic *Escherichia coli*. tEPEC = typical enteropathogenic *Escherichia coli*.

For peer review only

For peer review only - http://bmjopen.bmj.com/site/about/guidelines.xhtml

Table S3. Pathogen-specific attributable fractions of antibiotic courses for diarrhea by site among 1715 children in the MAL-ED cohort.

	Dhaka, Bangladesh	Fortaleza, Brazil	Vellore, India	Bhaktapur, Nepal	Loreto, Peru	Naushero Feroze, Pakistan	Venda, South Africa	Tanzania
Shigella	21.4 (18.6, 25.6)	10.6 (0.0, 29.6)	17.0 (11.8, 23.3)	15.0 (11.1, 19.9)	11.6 (8.7, 14.9)	6.4 (4.8, 8.5)	6.9 (0.0, 15.0)	7.9 (1.0, 16.0)
Rotavirus	16.0 (13.7, 19.1)	0.0 (0.0, 0.0)	12.2 (7.6, 17.4)	7.9 (5.4, 11.0)	5.5 (3.9, 7.5)	5.5 (4.4, 7.1)	3.3 (0.0, 11.6)	12.4 (6.1, 19.9)
Sapovirus	8.5 (5.5, 11.4)	15.1 (0.0, 37.7)	9.9 (5.8, 14.7)	9.6 (6.2, 13.5)	11.6 (9.0, 14.7)	5.8 (3.3, 8.7)	3.6 (0.2, 7.8)	10.9 (4.4, 18.2)
Adenovirus 40/41	23.9 (20.0, 29.1)	6.8 (0.0, 24.3)	7.9 (3.8, 13.0)	2.1 (0.1, 4.6)	6.5 (4.6, 9.0)	1.3 (0.1, 3.3)	1.6 (0.0, 4.6)	7.1 (1.7, 14.5)
ST-ETEC	14.7 (11.2, 18.9)	3.7 (0.0, 15.8)	7.3 (3.7, 11.7)	5.9 (3.5, 9.2)	3.2 (1.5, 5.6)	3.1 (1.7, 5.5)	5.4 (0.0, 15.6)	15.6 (6.6, 25.2)
Astrovirus	5.2 (2.4, 8.2)	0.0 (0.0, 0.0)	6.7 (3.4, 11.3)	3.1 (1.5, 5.0)	8.8 (6.5, 12.2)	4.8 (2.8, 7.2)	6.2 (0.6, 16.1)	2.0 (0.0, 5.8)
Norovirus	5.0 (2.9, 7.1)	6.5 (0.0, 21.8)	2.9 (0.4, 5.9)	5.3 (3.2, 8.4)	8.9 (6.5, 11.8)	2.3 (0.9, 5.1)	4.5 (0.0, 13.2)	12.3 (6.3, 19.9)
C. jejuni/C. coli	2.7 (0.3, 6.8)	0.0 (0.0, 0.0)	4.6 (1.6, 8.2)	3.1 (0.6, 6.9)	9.6 (5.9, 13.4)	1.9 (0.1, 4.5)	2.2 (0.0, 7.3)	4.1 (0.0, 13.3)
Cryptosporidium	1.9 (0.6, 3.3)	3.7 (0.0, 13.5)	4.7 (1.9, 9.8)	2.5 (0.6, 4.7)	3.7 (1.9, 5.6)	1.5 (0.6, 2.9)	0.1 (0.0, 1.2)	2.9 (0.0, 8.7)
tEPEC	1.1 (0.1, 3.0)	0.0 (0.0, 1.5)	1.8 (0.2, 4.8)	1.2 (0.2, 3.3)	1.6 (0.2, 3.5)	2.5 (0.4, 5.8)	0.0 (0.0, 2.1)	4.4 (0.0, 12.9)

Data are attributable fraction percentages with 95% confidence intervals (CIs). Abbreviations: *C. jejuni/C. coli* = *Campylobacter jejuni/Campylobacter coli*. ST-ETEC= heat-stable enterotoxigenic *Escherichia coli*. tEPEC = typical enteropathogenic *Escherichia coli*.

For peer review only

Table S4. Pathogen-specific attributable fractions of all antibiotic courses by site among 1715 children in the MAL-ED cohort.

	Dhaka, Bangladesh	Fortaleza, Brazil	Vellore, India	Bhaktapur, Nepal	Loreto, Peru	Naushero Feroze, Pakistan	Venda, South Africa	Haydom, Tanzania
Shigella	5.2 (4.5, 6.2)	0.9 (0.0, 2.4)	2.4 (1.6, 3.2)	4.5 (3.3, 6.0)	3.9 (2.9, 5.0)	2.4 (1.8, 3.2)	0.8 (0.0, 1.8)	1.4 (0.2, 2.9)
Rotavirus	3.9 (3.3, 4.6)	0.0 (0.0, 0.0)	1.7 (1.1, 2.4)	2.4 (1.6, 3.3)	1.9 (1.3, 2.5)	2.1 (1.6, 2.6)	0.4 (0.0, 1.4)	2.3 (1.1, 3.7)
Sapovirus	2.1 (1.3, 2.8)	1.2 (0.0, 3.0)	1.4 (0.8, 2.0)	2.9 (1.9, 4.1)	3.9 (3.0, 5.0)	2.2 (1.2, 3.2)	0.4 (0.0, 1.0)	2.0 (0.8, 3.3)
Adenovirus 40/41	5.8 (4.9, 7.1)	0.5 (0.0, 2.0)	1.1 (0.5, 1.8)	0.6 (0.0, 1.4)	2.2 (1.5, 3.1)	0.5 (0.0, 1.2)	0.2 (0.0, 0.6)	1.3 (0.3, 2.7)
ST-ETEC	3.6 (2.7, 4.6)	0.3 (0.0, 1.3)	1.0 (0.5, 1.6)	1.8 (1.0, 2.8)	1.1 (0.5, 1.9)	1.2 (0.6, 2.0)	0.7 (0.0, 1.9)	2.9 (1.2, 4.6)
Astrovirus	1.3 (0.6, 2.0)	0.0 (0.0, 0.0)	0.9 (0.5, 1.6)	0.9 (0.5, 1.5)	3.0 (2.2, 4.1)	1.8 (1.1, 2.7)	0.8 (0.1, 2.0)	0.4 (0.0, 1.1)
Norovirus	1.2 (0.7, 1.7)	0.5 (0.0, 1.8)	0.4 (0.1, 0.8)	1.6 (1.0, 2.5)	3.0 (2.2, 4.0)	0.9 (0.3, 1.9)	0.5 (0.0, 1.6)	2.3 (1.2, 3.7)
C. jejuni/C. coli	0.6 (0.1, 1.7)	0.0 (0.0, 0.0)	0.6 (0.2, 1.1)	0.9 (0.2, 2.1)	3.2 (2.0, 4.5)	0.7 (0.0, 1.7)	0.3 (0.0, 0.9)	0.8 (0.0, 2.4)
Cryptosporidium	0.5 (0.1, 0.8)	0.3 (0.0, 1.1)	0.6 (0.3, 1.4)	0.8 (0.2, 1.4)	1.2 (0.6, 1.9)	0.5 (0.2, 1.1)	0.0 (0.0, 0.2)	0.5 (0.0, 1.6)
tEPEC	0.3 (0.0, 0.7)	0.0 (0.0, 0.1)	0.3 (0.0, 0.7)	0.4 (0.1, 1.0)	0.5 (0.1, 1.2)	0.9 (0.1, 2.2)	0.0 (0.0, 0.3)	0.8 (0.0, 2.4)

Data are attributable fraction percentages with 95% confidence intervals (CIs). Abbreviations: *C. jejuni/C. coli* = *Campylobacter jejuni/Campylobacter coli*. ST-ETEC= heat-stable enterotoxigenic *Escherichia coli*. tEPEC = typical enteropathogenic *Escherichia coli*.

For peer review only

Table S5. Attributable incidence of pathogen-specific antibiotic courses for diarrhea by site among 1715 children in the MAL-ED cohort.

	Dhaka, Bangladesh	Fortaleza, Brazil	Vellore, India	Bhaktapur, Nepal	Loreto, Peru	Naushero Feroze, Pakistan	Venda, South Africa	Haydom, Tanzania
Shigella	45.79 (39.70, 54.61)	0.58 (0.00, 1.62)	9.08 (6.27, 12.4)	10.57 (7.79, 14.01)	20.54 (15.49, 26.48)	23.93 (17.88, 31.62)	0.90 (0.00, 1.96)	5.12 (0.65, 10.42)
Rotavirus	34.20 (29.21, 40.74)	0.00 (0.00, 0.00)	6.48 (4.02, 9.29)	5.53 (3.79, 7.73)	9.83 (6.85, 13.26)	20.68 (16.33, 26.47)	0.43 (0.00, 1.51)	8.08 (3.94, 12.95)
Sapovirus	18.10 (11.79, 24.44)	0.82 (0.00, 2.06)	5.29 (3.09, 7.82)	6.71 (4.38, 9.49)	20.50 (16.03, 26.07)	21.64 (12.24, 32.38)	0.47 (0.03, 1.02)	7.10 (2.88, 11.86)
Adenovirus 40/41	50.99 (42.72, 62.14)	0.37 (0.00, 1.33)	4.23 (2.04, 6.91)	1.47 (0.10, 3.24)	11.52 (8.13, 16.04)	4.87 (0.32, 12.21)	0.20 (0.00, 0.60)	4.64 (1.14, 9.44)
ST-ETEC	31.42 (23.92, 40.30)	0.20 (0.00, 0.86)	3.90 (1.98, 6.21)	4.18 (2.45, 6.46)	5.67 (2.65, 9.92)	11.52 (6.48, 20.50)	0.70 (0.00, 2.04)	10.14 (4.27, 16.42)
Astrovirus	11.18 (5.02, 17.56)	0.00 (0.00, 0.00)	3.59 (1.81, 6.02)	2.20 (1.08, 3.55)	15.67 (11.49, 21.70)	17.74 (10.62, 27.00)	0.82 (0.07, 2.11)	1.29 (0.00, 3.77)
Norovirus	10.64 (6.13, 15.20)	0.36 (0.00, 1.19)	1.56 (0.22, 3.12)	3.74 (2.23, 5.89)	15.80 (11.53, 20.94)	8.76 (3.24, 19.05)	0.59 (0.00, 1.73)	8.01 (4.08, 12.98)
C. jejuni/C. coli	5.70 (0.68, 14.56)	0.00 (0.00, 0.00)	2.46 (0.83, 4.38)	2.20 (0.45, 4.82)	17.06 (10.47, 23.71)	6.96 (0.41, 16.90)	0.29 (0.00, 0.96)	2.67 (0.00, 8.62)
Cryptosporidium	4.08 (1.19, 7.00)	0.20 (0.00, 0.74)	2.48 (1.02, 5.24)	1.77 (0.41, 3.29)	6.47 (3.39, 9.97)	5.50 (2.43, 10.81)	0.01 (0.00, 0.16)	1.87 (0.00, 5.64)
tEPEC	2.43 (0.29, 6.41)	0.00 (0.00, 0.08)	0.99 (0.08, 2.56)	0.88 (0.14, 2.30)	2.82 (0.37, 6.23)	9.26 (1.49, 21.69)	0.00 (0.00, 0.28)	2.86 (0.00, 8.39)

Data are attributable incidence rates per 100 child years with 95% confidence intervals (CIs). These data also reported in Figure 1B. Abbreviations: *C. jejuni/C. coli* = *Campylobacter jejuni/Campylobacter coli*. ST-ETEC= heat-stable enterotoxigenic *Escherichia coli*. tEPEC = typical enteropathogenic *Escherichia coli*.

For peer review only - http://bmjopen.bmj.com/site/about/guidelines.xhtml

Figure S1. Attributable incidence of pathogen-specific antibiotic courses for diarrhea in the first and second year of life among 1715 children in the MAL-ED cohort. Error bars show 95% CI. *C. jejuni/C. coli* = *Campylobacter jejuni/Campylobacter coli*. ST-ETEC= heat-stable enterotoxigenic *Escherichia coli*. tEPEC = typical enteropathogenic *Escherichia coli*.

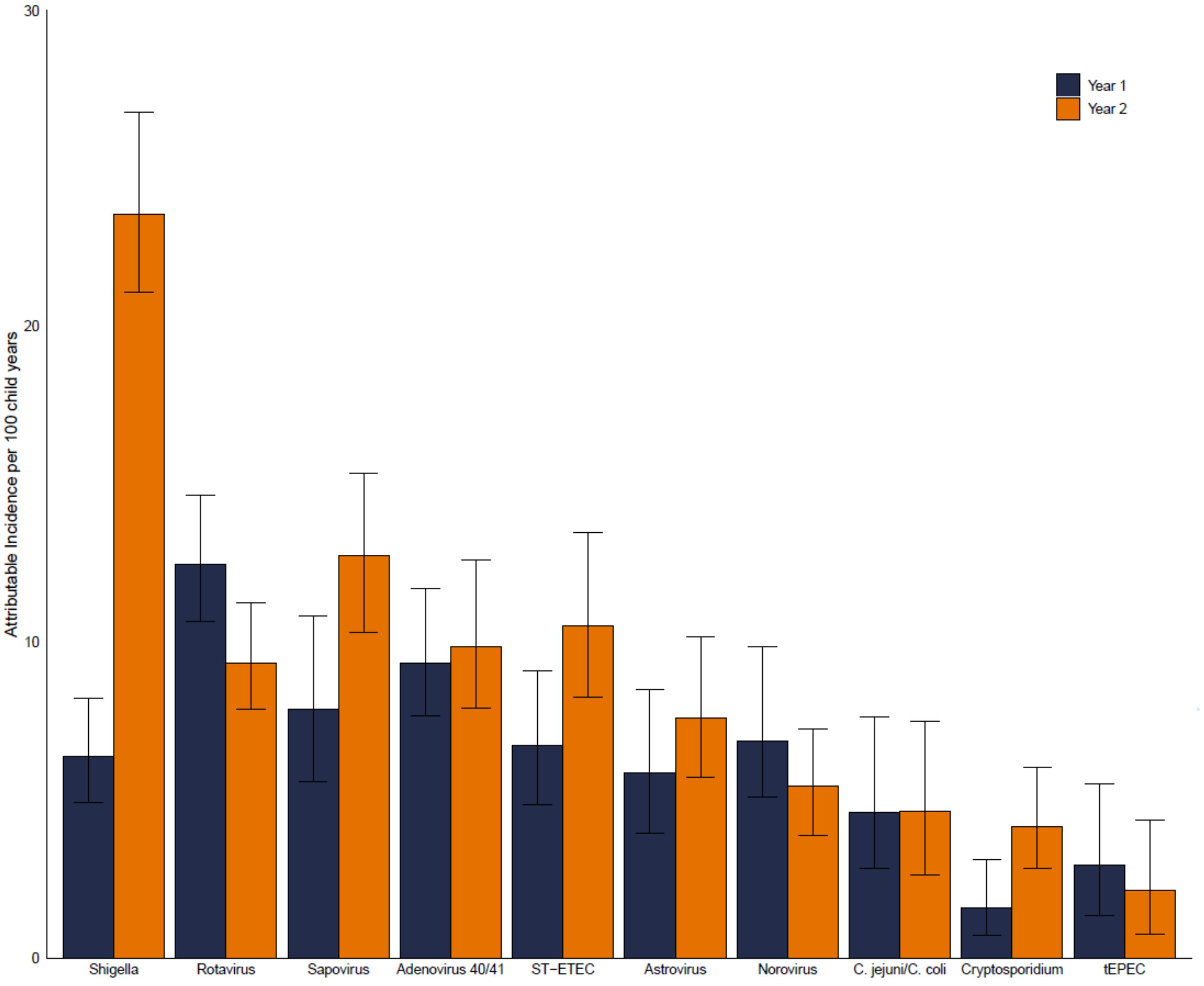

Table S6. Attributable incidence of pathogen-specific antibiotic courses for diarrhea in the first and second year of life among 1715 children in the MAL-ED cohort.

	Year 1 (0-11 months)	Year 2 (12-23 months)
Shigella	6.39 (4.91, 8.21)	23.55 (21.08, 26.79)
Rotavirus	12.48 (10.67, 14.65)	9.33 (7.87, 11.24)
Sapovirus	7.89 (5.58, 10.84)	12.75 (10.32, 15.36)
Adenovirus 40/41	9.35 (7.67, 11.71)	9.87 (7.92, 12.61)
ST-ETEC	6.74 (4.87, 9.10)	10.52 (8.26, 13.49)
Astrovirus	5.87 (3.94, 8.49)	7.61 (5.72, 10.17)
Norovirus	6.86 (5.09, 9.86)	5.43 (3.88, 7.25)
C. jejuni/C. coli	4.62 (2.85, 7.62)	4.65 (2.63, 7.49)
Cryptosporidium	1.60 (0.72, 3.10)	4.15 (2.82, 6.03)
tEPEC	2.95 (1.35, 5.52)	2.13 (0.77, 4.38)

Data are attributable incidence rates per 100 child years with 95% confidence intervals (CIs). These data also reported in Figure S1. Abbreviations: *C. jejuni/C. coli* = *Campylobacter jejuni/Campylobacter coli*. ST-ETEC= heat-stable enterotoxigenic *Escherichia coli*. tEPEC = typical enteropathogenic *Escherichia coli*.

For peer review only - http://bmjopen.bmj.com/site/about/guidelines.xhtml

Table S7. Pathogen-specific attributable fractions of all antibiotic courses by antibiotic drug class among 1715 children in the MAL-ED cohort.

	Any Antibiotic	Cephalosporins	Fluoroquinolones	Macrolides	Metronidazole	Penicillins	Sulfonamides
Shigella	3.2 (2.9, 3.7)	2.0 (1.6, 2.6)	12.2 (10.5, 14.5)	5.5 (4.7, 6.6)	6.7 (5.5, 8.0)	0.9 (0.7, 1.2)	3.6 (2.8, 4.7)
Rotavirus	2.4 (2.1, 2.7)	1.4 (1.1, 1.9)	7.1 (5.4, 9.0)	4.0 (3.3, 4.8)	4.7 (3.8, 5.9)	1.0 (0.7, 1.3)	3.0 (2.0, 4.1)
Sapovirus	2.2 (1.8, 2.7)	1.4 (0.9, 2.0)	5.9 (4.3, 7.5)	3.3 (2.6, 4.1)	5.3 (3.9, 7.1)	0.9 (0.6, 1.1)	3.4 (2.6, 4.5)
Adenovirus 40/41	2.1 (1.8, 2.6)	1.0 (0.7, 1.4)	6.9 (5.6, 8.8)	5.3 (4.5, 6.4)	2.1 (1.4, 3.3)	1.0 (0.8, 1.4)	1.5 (1.0, 2.2)
ST-ETEC	1.9 (1.5, 2.3)	0.8 (0.5, 1.3)	6.5 (4.9, 8.4)	3.3 (2.5, 4.2)	3.4 (2.5, 4.7)	0.8 (0.5, 1.1)	2.2 (1.4, 3.2)
Astrovirus	1.5 (1.1, 1.9)	1.4 (0.9, 2.0)	3.0 (1.9, 4.3)	2.0 (1.4, 2.8)	3.2 (2.1, 4.5)	0.7 (0.5, 0.9)	2.2 (1.5, 3.1)
Norovirus	1.3 (1.1, 1.8)	0.5 (0.2, 0.9)	2.3 (1.4, 3.4)	1.8 (1.3, 2.4)	2.6 (1.7, 4.2)	0.8 (0.6, 1.1)	2.5 (1.9, 3.6)
C. jejuni/C. coli	1.0 (0.7, 1.6)	0.6 (0.3, 1.0)	1.7 (0.8, 3.3)	1.8 (1.1, 2.8)	1.7 (0.6, 3.2)	0.5 (0.3, 0.7)	1.9 (1.3, 2.9)
Cryptosporidium	0.6 (0.4, 0.9)	0.4 (0.2, 0.7)	1.5 (0.7, 2.4)	0.7 (0.3, 1.0)	1.2 (0.7, 2.1)	0.4 (0.2, 0.6)	1.0 (0.5, 1.6)
tEPEC	0.6 (0.3, 1.0)	0.5 (0.1, 1.1)	0.7 (0.3, 1.5)	0.5 (0.2, 1.0)	1.8 (0.6, 3.8)	0.3 (0.1, 0.6)	0.9 (0.4, 1.8)

Data are attributable fraction percentages with 95% confidence intervals (CIs). These data also reported in Figure 2B. Abbreviations: *C. jejuni/C. coli* = *Campylobacter jejuni/Campylobacter coli*. ST-ETEC= heat-stable enterotoxigenic *Escherichia coli*. tEPEC = typical enteropathogenic *Escherichia coli*.

For peer review only

For peer review only - http://bmjopen.bmj.com/site/about/guidelines.xhtml

Table S8. Pathogen-specific attributable fractions of antibiotic courses for dysentery and watery diarrhea among 1,715 children in the MAL-ED cohort

	**Attributable fraction of antibiotic courses for dysentery**	**Attributable fraction of antibiotic courses for watery diarrhea**	**Proportion of all attributable antibiotic courses that were for dysentery**
Shigella	27.5% (23.4, 33.1)	10.3% (9.2, 11.8)	18.7% (16.2, 21.4)
Rotavirus	3.8% (2.3, 5.8)	9.1% (8.1, 10.4)	3.5% (2.1, 5.1)
Sapovirus	5.9% (3.8, 8.6)	8.3% (6.8, 10.2)	5.8% (3.9, 7.9)
Adenovirus 40/41	7.4% (5.6, 10.2)	7.6% (6.5, 9.3)	7.8% (5.9, 9.9)
ST-ETEC	5.7% (3.2, 8.4)	6.9% (5.7, 8.7)	6.7% (4.1, 9.1)
Astrovirus	2.6% (1.5, 4.3)	5.6% (4.3, 7.3)	3.9% (2.5, 5.8)
Norovirus	4.0% (2.3, 6.3)	4.9% (3.8, 6.5)	6.6% (4.0, 9.6)
C. jejuni/C. coli	8.5% (5.8, 12.2)	3.2% (2.1, 5.4)	18.6% (12.7, 25.5)
Cryptosporidium	1.3% (0.5, 3.0)	2.3% (1.6, 3.4)	4.7% (1.7, 9.0)
tEPEC	1.5% (0.5, 3.5)	2.1% (1.0, 3.8)	6.0% (2.2, 11.8)

Data are attributable fraction percentages with 95% confidence intervals (CIs). [a] Positive value indicates the pathogen was responsible for a larger proportion of antibiotic-treated dysentery diarrheal episodes compared to antibiotic-treated watery diarrhea episodes. Abbreviations: *C. jejuni/C. coli* = *Campylobacter jejuni/Campylobacter coli*. ST-ETEC= heat-stable enterotoxigenic *Escherichia coli*. tEPEC = typical enteropathogenic *Escherichia coli*.

For peer review only

For peer review only - http://bmjopen.bmj.com/site/about/guidelines.xhtml

Table S9 Risk ratios for antibiotic treatment comparing episode-specific attributable fractions for each pathogen by site among 1715 children in the MAL-ED cohort.

	Dhaka, Bangladesh	Vellore, India	Bhaktapur, Nepal	Loreto, Peru	Naushero Feroze, Pakistan	Haydom, Tanzania
Shigella	1.40 (1.22, 1.61)	1.55 (1.03, 2.33)	2.30 (1.70, 3.11)	1.35 (1.08, 1.68)	1.38 (1.17, 1.62)	0.77 (0.34, 1.72)
Rotavirus	1.10 (0.95, 1.27)	1.67 (1.13, 2.46)	0.70 (0.46, 1.05)	1.37 (1.06, 1.79)	1.48 (1.31, 1.68)	1.04 (0.64, 1.69)
Sapovirus	1.04 (0.83, 1.29)	0.93 (0.53, 1.62)	0.76 (0.49, 1.18)	0.86 (0.68, 1.09)	1.02 (0.84, 1.25)	1.23 (0.77, 1.98)
Adenovirus 40/41	1.01 (0.87, 1.18)	1.85 (0.84, 4.07)	1.07 (0.35, 3.21)	0.73 (0.49, 1.10)	..	1.65 (0.84, 3.26)
ST-ETEC	0.87 (0.74, 1.03)	0.84 (0.48, 1.46)	0.74 (0.48, 1.13)	0.91 (0.61, 1.36)	1.32 (1.01, 1.71)	0.87 (0.56, 1.37)
Astrovirus	1.00 (0.74, 1.34)	1.08 (0.57, 2.02)	0.54 (0.26, 1.11)	0.94 (0.70, 1.26)	1.08 (0.88, 1.34)	0.49 (0.10, 2.37)
Norovirus	1.40 (1.08, 1.80)	0.66 (0.20, 2.14)	0.58 (0.33, 1.04)	1.12 (0.83, 1.50)	1.36 (1.01, 1.83)	1.13 (0.67, 1.88)
C. jejuni/C. coli	0.66 (0.43, 1.02)	0.75 (0.33, 1.69)	..	1.43 (1.15, 1.78)	0.87 (0.58, 1.32)	..
Cryptosporidium	1.03 (0.71, 1.48)	2.11 (1.18, 3.79)	1.31 (0.64, 2.66)	0.72 (0.46, 1.15)	1.19 (0.83, 1.71)	3.18 (1.36, 7.43)
tEPEC	0.44 (0.22, 0.89)	..	0.41 (0.12, 1.38)	0.99 (0.54, 1.81)	0.97 (0.74, 1.28)	1.87 (0.98, 3.57)

Data are risk ratios (RR) with 95% confidence intervals (CIs). The model is adjusted for: all pathogens, age, sex, and the water, assets, maternal education, income (WAMI) index. Site data from Brazil and South Africa were removed as there were not enough diarrheal episodes to model the data. Select pathogen data by site is missing in cases where there were no treated diarrheal episodes. Abbreviations: *C. jejuni/C. coli* = *Campylobacter jejuni/Campylobacter coli*. ST-ETEC= heat-stable enterotoxigenic *Escherichia coli*. tEPEC = typical enteropathogenic *Escherichia coli*.

For peer review only

Table S10. Mediation analysis assessing whether diarrhea severity and dysentery mediated the relationship between Shigella diarrhea and antibiotic treatment among 1715 children in the MAL-ED cohort.

	Shigella (Mediated by diarrhea severity)		*Shigella* (Mediated by dysentery)	
	Any antibiotic	Fluoroquinolones or macrolides	Any antibiotic	Fluoroquinolones or macrolides
Total Effect Rate Ratio	1.30 (1.21, 1.40)	1.39 (1.21, 1.58)	1.30 (1.21, 1.40)	1.39 (1.21, 1.58)
Pure Natural Direct Effect Rate Ratio	1.29 (1.18, 1.40)	1.33 (1.14, 1.55)	1.25 (1.14, 1.37)	1.23 (1.05, 1.44)
Total Natural Indirect Effect Rate Ratio	1.01 (0.96, 1.06)	1.04 (0.95, 1.14)	1.04 (0.99, 1.10)	1.13 (1.02, 1.25)
Proportion Mediated	0.05 (0.00, 0.27)	0.14 (0.00, 0.47)	0.18 (0.00, 0.43)	0.40 (0.08, 0.82)

Data are risk ratios (RR) with 95% confidence intervals (CIs).

For peer review only

For peer review only - http://bmjopen.bmj.com/site/about/guidelines.xhtml

STROBE Statement—Checklist of items that should be included in reports of ***cohort studies***

	Item No	Recommendation	Page No
**Title and abstract**	1	(*a*) Indicate the study's design with a commonly used term in the title or the abstract	2
		(*b*) Provide in the abstract an informative and balanced summary of what was done and what was found	2-3
**Introduction**			
Background/rationale	2	Explain the scientific background and rationale for the investigation being reported	5-6
Objectives	3	State specific objectives, including any prespecified hypotheses	6
**Methods**			
Study design	4	Present key elements of study design early in the paper	7
Setting	5	Describe the setting, locations, and relevant dates, including periods of recruitment, exposure, follow-up, and data collection	7
Participants	6	(*a*) Give the eligibility criteria, and the sources and methods of selection of participants. Describe methods of follow-up	7
		(*b*) For matched studies, give matching criteria and number of exposed and unexposed	8-10
Variables	7	Clearly define all outcomes, exposures, predictors, potential confounders, and effect modifiers. Give diagnostic criteria, if applicable	8-10
Data sources/ measurement	8*	For each variable of interest, give sources of data and details of methods of assessment (measurement). Describe comparability of assessment methods if there is more than one group	8-10
Bias	9	Describe any efforts to address potential sources of bias	N/A; secondary data analysis; parent study cited
Study size	10	Explain how the study size was arrived at	N/A; secondary data analysis; parent study cited
Quantitative variables	11	Explain how quantitative variables were handled in the analyses. If applicable, describe which groupings were chosen and why	8-10
Statistical methods	12	(*a*) Describe all statistical methods, including those used to control for confounding	8-10
		(*b*) Describe any methods used to examine subgroups and interactions	N/A
		(*c*) Explain how missing data were addressed	N/A
		(*d*) If applicable, explain how loss to follow-up was addressed	N/A; secondary data analysis; parent study cited
		(*e*) Describe any sensitivity analyses	N/A

For peer review only - http://bmjopen.bmj.com/site/about/guidelines.xhtml

**Results**			
Participants	13*	(a) Report numbers of individuals at each stage of study—eg numbers potentially eligible, examined for eligibility, confirmed eligible, included in the study, completing follow-up, and analysed	10
		(b) Give reasons for non-participation at each stage	N/A; secondary data analysis; parent study cited
		(c) Consider use of a flow diagram	N/A; secondary data analysis; parent study cited
Descriptive data	14*	(a) Give characteristics of study participants (eg demographic, clinical, social) and information on exposures and potential confounders	23; Table 1
		(b) Indicate number of participants with missing data for each variable of interest	N/A
		(c) Summarise follow-up time (eg, average and total amount)	10
Outcome data	15*	Report numbers of outcome events or summary measures over time	23 (Table 1)
Main results	16	(*a*) Give unadjusted estimates and, if applicable, confounder-adjusted estimates and their precision (eg, 95% confidence interval). Make clear which confounders were adjusted for and why they were included	13-14; 24 (Table 2). Figs 1-3
		(*b*) Report category boundaries when continuous variables were categorized	N/A
		(*c*) If relevant, consider translating estimates of relative risk into absolute risk for a meaningful time period	N/A
Other analyses	17	Report other analyses done—eg analyses of subgroups and interactions, and sensitivity analyses	11-14; Supplemental material (S1-S10; Fig S1)
**Discussion**			
Key results	18	Summarise key results with reference to study objectives	14-16
Limitations	19	Discuss limitations of the study, taking into account sources of potential bias or imprecision. Discuss both direction and magnitude of any potential bias	16-17
Interpretation	20	Give a cautious overall interpretation of results considering objectives, limitations, multiplicity of analyses, results from similar studies, and other relevant evidence	15-17
Generalisability	21	Discuss the generalisability (external validity) of the study results	17
**Other information**			
Funding	22	Give the source of funding and the role of the funders for the present study and, if applicable, for the original study on which the present article is based	18-19

*Give information separately for exposed and unexposed groups.

For peer review only

Note: An Explanation and Elaboration article discusses each checklist item and gives methodological background and published examples of transparent reporting. The STROBE checklist is best used in conjunction with this article (freely available on the Web sites of PLoS Medicine at http://www.plosmedicine.org/, Annals of Internal Medicine at http://www.annals.org/, and Epidemiology at http://www.epidem.com/). Information on the STROBE Initiative is available at http://www.strobe-statement.org.

For peer review only

For peer review only - http://bmjopen.bmj.com/site/about/guidelines.xhtml
